# Tackling childbirth-related intrusive memories with a single-session behavioural intervention involving a visuospatial task: protocol for a single-blind, waitlist-controlled randomised trial

**DOI:** 10.1136/bmjopen-2023-073874

**Published:** 2023-05-29

**Authors:** Déborah Fort, Camille Deforges, Nadine Messerli-Bürgy, Tanja Michael, David Baud, Joan Lalor, Ulrike Rimmele, Antje Horsch

**Affiliations:** 1 Institute of Higher Education and Research in Healthcare, Faculty of Biology and Medicine, University of Lausanne, Lausanne, Switzerland; 2 Family and Development Research Center (FADO), Faculty of Social and Political Sciences, University of Lausanne, Lausanne, Switzerland; 3 Division of Clinical Psychology and Psychotherapy, Department of Psychology, Saarland University, Saarbrücken, Germany; 4 Materno-Fetal and Obstetrics Research Unit, Department Woman-Mother-Child, Lausanne University Hospital, Lausanne, Switzerland; 5 School of Nursing and Midwifery, Trinity College Dublin, Dublin 2, Ireland; 6 Emotion and Memory Laboratory, Faculty of Education Sciences and Psychology, University of Geneva, Geneva, Switzerland; 7 Swiss Center for Affective Sciences (CISA), University of Geneva, Geneva, Switzerland; 8 Neonatology Service, Department Woman-Mother-Child, Lausanne University Hospital, Lausanne, Switzerland

**Keywords:** postpartum period, adult psychiatry, clinical trial, life change events, postpartum women

## Abstract

**Introduction:**

Approximately 12.3% of mothers experience childbirth-related post-traumatic stress symptoms (CB-PTSS). However, evidence-based interventions to treat CB-PTSS are lacking. Intrusive memories (IM), a key CB-PTSS, are distressing and can trigger other PTSS by reliving the traumatic event. Emerging evidence shows that a behavioural intervention involving a visuospatial task (BI-VT) can reduce the number of IM and PTSS, supposedly by interfering with the reconsolidation of the trauma memory. This study aims to test the efficacy of a single-session BI-VT targeting IM to reduce the number of childbirth-related (CB-)IM and PTSS, in comparison to a waitlist control group (WCG).

**Methods and analysis:**

In this multicentre, single-blind, randomised controlled trial being undertaken at one regional and one university hospital in Switzerland, 60 participants will be allocated to the Immediate Intervention Group (IIG), receiving the immediate intervention on day 15, and 60 participants to the WCG receiving the delayed intervention on day 30. All participants will report their CB-IM during the 2 weeks preimmediate and postimmediate intervention in diaries. The IIG will additionally report their CB-IM over weeks 5 and 6 postimmediate intervention. Self-report questionnaires will assess CB-PTSS at 2 weeks preimmediate and postimmediate intervention in both groups, and at 6 weeks postimmediate intervention in the IIG. A feedback questionnaire will evaluate the intervention acceptability. The primary outcome will be group differences in the number of CB-IM between the 2 weeks preimmediate and postimmediate intervention. Secondary outcomes will be CB-PTSS at 2 and 6 weeks postimmediate intervention, the number of CB-IM at weeks 5 and 6 postimmediate intervention, and intervention acceptability.

**Ethics and dissemination:**

Ethical approval was granted by the Human Research Ethics Committee of the Canton of Vaud (study number 202200652). Participants will provide an informed consent before study participation. Results will be presented in peer-reviewed journals and at conferences.

**Trial registration number:**

NCT05381155.

**Strengths and limitations of this study**

- This study aims to contribute to the development of an innovative, single-session and easily accessible intervention to reduce childbirth-related post-traumatic stress symptoms (CB-PTSS).
- A pilot study has already shown high efficacy and acceptability of this single-session intervention.
- CB-PTSS will be assessed with a validated questionnaire specifically developed for the perinatal context.
- Participants will be blinded to group allocation, but the research team will not.
- The waitlist control group is not an active comparator.

## Introduction

### Childbirth-related post-traumatic stress symptoms and intrusive memories

Approximately 12.3% of mothers develop childbirth-related post-traumatic stress symptoms (CB-PTSS) and 4.7% of mothers meet the diagnostic criteria for childbirth-related post-traumatic stress disorder (CB-PTSD).^1^ PTSD is a psychiatric disorder, highly comorbid with anxiety and depression,^2^ with distressing symptoms including intrusions, avoidance of trauma-related cues, negative alterations in cognitions and mood and alterations in arousal and reactivity.^3^ Intrusive memories (IM), a core symptom of PTSD, are recurrent and involuntary recollections of the trauma memory.^4^ IM are vivid, multisensory, predominantly visual, fragments of the trauma and are highly distressing.^5^ CB-PTSS negatively affect various facets of family life, such as the mother–infant relationship, breastfeeding and child development.^6^ Despite their high prevalence, there is currently only limited evidence concerning the effectiveness of interventions to reduce CB-PTSS.^7^ Cognitive-behavioural therapy is currently one of the most common evidence-based interventions for PTSD, but 40%–50% of patients are unresponsive to it.^8^ Furthermore, access to such interventions is limited due to costs arising from numerous sessions with highly trained clinicians and lack of such therapists. Thus, it is essential to develop brief, easily accessible and resource-efficient psychological interventions adapted to the perinatal context.

For such interventions, IM are targets of particular interest since they involve re-experiencing distressing moments of the trauma and may trigger or maintain other PTSS.^9^ For example, high frequency of IM is associated with more difficulties in concentration.^10^ New interventions targeting IM could therefore reduce not only intrusions but also other (CB-)PTSS, such as avoidance symptoms,^11^ and may thus be an innovative solution to support traumatised individuals.

### Interventions assumed to tackle IM through interference with memory reconsolidation

Each new event initiates memory consolidation, a time-dependent process during which memories are malleable until their stabilisation into long-term memory within hours.^12 13^ Acute emotional experiences (eg, traumatic events) trigger a physiological stress response that modulates memory consolidation, resulting in particularly strong and lasting memories.^14^ Furthermore, in healthy volunteers, physiological stress reactivity during a traumatic film appears to increase the number of IM in the following days.^15^ This suggests that emotional arousal linked to traumatic events impacts memory formation, by enhancing the consolidation of distressing memories,^14^ and finally increase IM.

Although emerging evidence supports the efficacy of interventions assumed to interfere with trauma memory consolidation to prevent the development of PTSS,^16^ it is not always possible to intervene within the hours following a traumatic event.^17^ A growing body of literature supports the assumption that memories are not fixed forever after consolidation, but that they can become malleable again when reactivated, regardless of the time passed since the traumatic event.^18 19^ Reactivating a memory makes it malleable again for a few hours, until its restabilisation into long-term memory, corresponding to the reconsolidation process.^20^ Consequently, memory reconsolidation is hypothesised to be a new window of opportunity to tackle IM^21^ and reduce PTSS.

Interventions assumed to interfere with memory (re)consolidation have used various interference strategies, such as pharmacological agents, psychological therapy techniques or behavioural interventions involving a visuospatial task (BI-VT).^16^ Such visuospatial tasks (eg, Tetris gameplay), applied after assumed memory reactivation, possibly interfere with the reconsolidation of traumatic images by taxing the same resources needed by the visuospatial working memory to restabilise them.^21 22^ These tasks appear as the most appropriate in the perinatal context (eg, no drug contraindications, accessible to any trained clinicians and minimum exposure to the trauma memory). So far, lab studies support that a visuospatial task delivered after the reactivation of a traumatic film memory leads to a reduction of the number of IM in healthy volunteers.^23 24^ Further, a few translational studies reported encouraging results regarding the efficacy of BI-VT on the number of IM and PTSS in clinical samples.^25 26^ For example, one BI-VT was applied to participants suffering from complex PTSD in an open-label single case series (n = 20).^25^ After repeated sessions, during which participants produced writing narratives of a self-selected IM and then played Tetris for 25 min, a mean reduction of 64% of the targeted IM frequency was observed, whereof 16 out of the 20 participants reported an IM frequency reduction of at least 50%. In another study, repeated sessions of a BI-VT including Tetris gameplay also resulted in a decrease of the number of IM of refugees in a small single case series (n = 4).^26^

### Developing an evidence-based single-session BI-VT adapted to the perinatal context

A single-session BI-VT was recently adapted to the perinatal context in a pre–post pilot study (n = 18).^27^ During the intervention, participants briefly narrated their childbirth on the maternity ward where they had given birth, as evidence suggests that being in a similar physical context as the one in which the traumatic event occurred improves the chances of successful memory reactivation.^28^ Narration of their childbirth, ensuring a reliable source of the original memory,^29^ lasted 10 min for a sufficient exposure to the reminder cues supposed to engage memory reconsolidation of strong and old memories,^30^ such as trauma-related memories. Participants then played Tetris during 20 min in a neutral room. This single-session BI-VT led to a median reduction of 82% of the number of childbirth-related (CB-)IM.^27^ Participants also reported an average reduction of 57% of CB-PTSS severity at 1 month postintervention and rated the intervention as highly acceptable. Characteristics (distress, nowness and sensorial modalities) of CB-IM were also described in this study, but a larger sample size is needed to better understand the nature of CB-IM and their responsiveness to such intervention.

These preliminary results suggest that a single-session BI-VT is suitable for the perinatal context. Building on this pilot study, a randomised controlled trial (RCT) will then allow to control for other potential confounding factors, such as the time between the trauma and the intervention.^31^ Since, to our knowledge, no equivalent single-session intervention aimed at reducing CB-IM exists, a comparative treatment control group was not applicable. Therefore, to test the efficacy of a BI-VT to reduce CB-PTSS and to allow all help-seeking participants to benefit from the likely positive effects of this BI-VT, this RCT will include a waitlist control group (WCG) as a comparator. As waitlists can create a nocebo condition, which might bias participants’ self-reported symptoms^32^ and artificially increase the estimated effects of the experimental intervention,^33^ participants will be blind to group allocation in an effort to limit these biases. Even though this RCT including a WCG will not allow to study the specific effect of the mechanisms of this BI-VT on CB-PTSS in comparison to other interventions, it will bring more evidence to define this BI-VT as potentially *efficacious* to reduce CB-PTSS.^31^ Given the lack of evidence supporting current interventions to treat established CB-PTSS,^34^ developing a single-session, low cost, easily accessible and evidence-based intervention would be of great clinical value and might reduce long-term difficulties of mothers after traumatic childbirth.

One challenge for the translational research on memory reconsolidation is to identify if each supposed memory process is taking place and at which point.^35^ No markers of these memory processes exist so far, but emotional arousal may be used to infer if participants have been sufficiently engaged in the narration of their traumatic memory to trigger memory reactivation. Previous findings revealed that a decrease in emotional arousal after playing Tetris is associated to an increased responsiveness to a similar BI-VT.^25^ Therefore, we will include emotional and physiological measures of arousal (eg, subjective distress and heart rate variability (HRV)^36 37^) to explore conditions enabling a successful implementation of a BI-VT in the perinatal context. In the future, such an assessment of physiological/emotional arousal could guide a personalised adaptation of this type of intervention.^38^

### Aims of the present study

**Figure 1:**
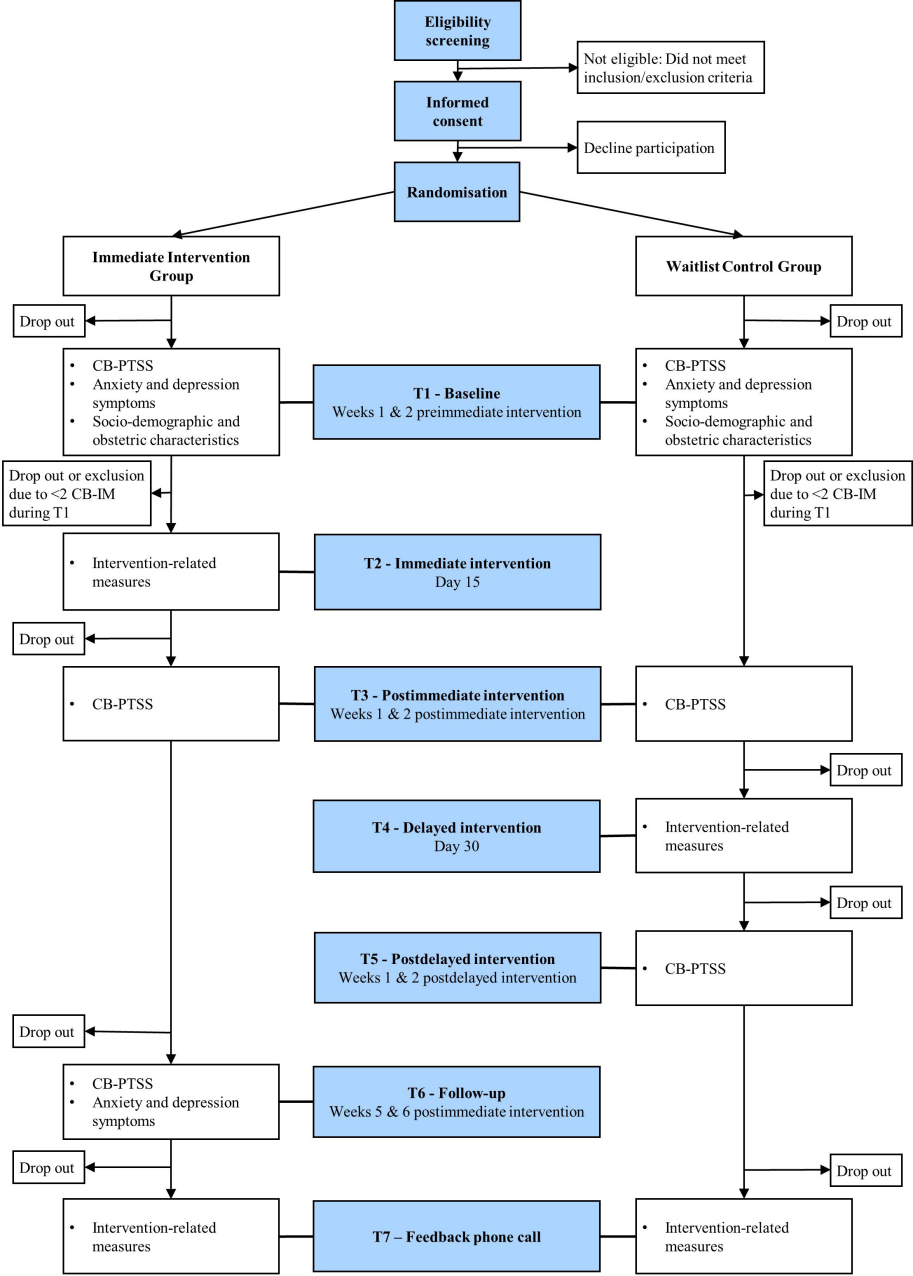
Study flowchart. CB-IM, childbirth-related intrusive memories; CB-PTSS, childbirth-related post-traumatic stress symptoms.

This study aims to test the efficacy of a single-session BI-VT in reducing the number of CB-IM and CB-PTSS, in comparison to a waitlist. This intervention is assumed to reactivate the childbirth memory and interfere with its reconsolidation, respectively through a brief childbirth narration and Tetris gameplay, leading to a decrease in CB-IM and CB-PTSS. The primary outcome will be the difference in the number of CB-IM between baseline (2 weeks preimmediate intervention) and the 2 weeks postimmediate intervention in the Immediate Intervention Group (IIG) compared with the WCG (see figure 1 for the study flowchart). Secondary outcomes will include group differences in CB-PTSS between baseline and at 2 weeks postimmediate intervention, as well as the acceptability of the intervention assessed in both groups. The following secondary outcomes will concern the IIG: the change in CB-PTSS between baseline and follow-up (at 6 weeks postimmediate intervention), and the change in the number of CB-IM between baseline and follow-up (weeks 5 and 6 postimmediate intervention). In addition, this study will explore, in both groups, the course of the nature (distress, nowness and sensorial modality) and content of CB-IM before and after the intervention, as well as associations between participants’ HRV and subjective distress course during the intervention, and the intervention efficacy. In the IIG, we will additionally explore the course of postnatal depression and anxiety symptoms at 6 weeks postimmediate intervention. Finally, this study will explore the change in the number of CB-IM and CB-PTSS between the 2 weeks predelayed and postdelayed intervention in the WCG.

## Methods and analysis

### Study design

The present study will be a single-blind, waitlist-controlled RCT. The trial will include two arms: the IIG receiving the immediate intervention on day 15, and the WCG receiving the same intervention on day 30 (delayed intervention).

### Population, recruitment, group allocation and blinding

Mothers with a minimum age of 18 years, who gave birth at least 6 weeks prior to the screening in one of the two study centres (one university hospital, being the main study centre, and one regional hospital from Switzerland), and suffer from CB-IM will be eligible to participate. To avoid a floor effect regarding CB-IM reduction, we will only include mothers reporting at least four CB-IM over the 2 weeks prior to the screening. Exclusion criteria will be insufficient French-speaking level to complete the assessments, established intellectual disability or psychotic illness, an ongoing psychological treatment in relation to childbirth, propranolol medication (due to suspected effects on memory reconsolidation^39^), alcohol and/or illicit drug abuse, being pregnant, perinatal loss of a child born during the index traumatic childbirth (ie, the childbirth triggering the CB-IM targeted during the intervention) or a life-threatening illness of the mother or infant(s). Moreover, mothers not able to distinguish potential IM related to another traumatic event from CB-IM linked to the index traumatic childbirth will be excluded. Importantly, data related to any participants that reported less than two CB-IM at baseline will be excluded from relevant analyses to avoid a floor effect, or if they stated having a concurrent psychological treatment in relation to childbirth during study participation.

Adverts will be displayed in nurseries and practices of midwives, gynaecologists and paediatricians. Study information will also be shared on social media. In addition, study centres will send a text message to invite potentially eligible mothers who gave birth in their hospitals to participate in the study.

During a phone call, the study coordinator (DF) will screen interested mothers. The presence and number of CB-IM over the past 2 weeks will be assessed with the item B1 of the Clinician-administered PTSD scale for Diagnostic and Statistical Manual of Mental Disorders, Fifth Edition, (DSM-5),^40^ and the T-ACE will be used to assess alcohol consumption.^41^ Eligible mothers who signed the consent form will be randomly allocated to the IIG or WCG with a 1:1 ratio. To ensure concealment, a scientific collaborator not involved in data collection created a block randomisation list stratified by centre, which will be automatically generated by the Research Electronic Data Capture (REDCap) software. To limit waitlist condition-related bias on participants’ self-reports,^32 33^ participants will be blind to group allocation. To that matter, participants will only be informed that both groups will complete the same assessments and receive the intervention, but in a different order. For an overview of study procedures for both groups, see figure 1. To improve completion of study assessments, the study coordinator will follow participants’ progression during the whole study and participants will receive reminders to complete questionnaires.

### Study procedures

#### T1: baseline

All participants will receive questionnaires regarding sociodemographic and obstetric information, postnatal depression and anxiety symptoms on the first day of the baseline. They will report their CB-IM in diaries during the 2 weeks preimmediate intervention. They will also report their CB-PTSS in a self-report questionnaire during week 2.

#### T2: immediate intervention

The IIG will receive the intervention on day 15. For a description of intervention procedures, see the Intervention section.

#### T3: postimmediate intervention

Both groups will report their CB-IM during the 2 weeks postimmediate intervention. To ensure blinding of WCG, this group will complete a diary covering T1 and T3. Both groups will report their CB-PTSS in a self-report questionnaire during week 2 postimmediate intervention.

#### T4: delayed intervention

The WCG will receive the delayed intervention on day 30. The delayed intervention follows the exact same procedures as the immediate intervention.

#### T5: postdelayed intervention

The WCG will report their CB-IM in a diary during the 2 weeks postdelayed intervention and their CB-PTSS in a self-report questionnaire during week 2 postdelayed intervention.

#### T6: follow-up

The IIG will report their CB-IM in a diary during weeks 5 and 6 postimmediate intervention. They will also complete self-report questionnaire assessing postnatal depression and anxiety symptoms, as well as CB-PTSS during week 6 postimmediate intervention. The WCG will not complete follow-up assessments to ensure a fair distribution of burden between groups

#### T7: feedback phone call

Both groups will complete a feedback questionnaire by phone.

### Intervention

The study coordinator, a trained psychologist, will meet the participant in a neutral room, inside a non-medical administrative building, explain intervention procedures, and attach an ECG device to their chest. To assess HRV baseline, the participant will count aloud during 2 min (Count aloud task A). The study coordinator and the participant will then go into a gynaecological examination room of the maternity ward to realise the first part of the intervention:

1. Childbirth narrative task (assumed memory reactivation phase): the participant will be asked to briefly narrate their childbirth orally during 10 min. The study coordinator will first ask participants to narrate the overall course of their childbirth during 5–7 min (Narrative task A). Then, the participant will be asked to narrate the moment associated to the most frequent or distressing CB-IM reported in the diary, for another 3–5 min (Narrative task B). If the participant perceived her distress to be overwhelming, she will have the option to go into another room not located in the maternity or to write about her childbirth instead of narrating it orally.

The participant and the study coordinator will then return to the neutral room to start the second part of the intervention, at least 10 min later, in order to allow the assumed memory labilisation^42^ and to avoid further memory reactivation during Tetris gameplay:

2. Tetris gameplay (assumed memory reconsolidation disruption phase): the study coordinator will ask the participant to play Tetris on a handheld gaming device (Nintendo 3DS), in ‘Marathon’ mode, with both sound and 3D switched off. The participant will be instructed to focus both on the block currently falling and on the upcoming blocks (shown in a preview box). To promote mental rotation, participants will be encouraged to use their imagination to visualise how to place each block to complete as many horizontal lines as possible.^24^ The participant will have a 3 min practice trial before playing Tetris for 20 min uninterrupted. The study coordinator will stay in the room to ensure that the participant will stay focused on Tetris during the 20 min-long gameplay (eg, instead of looking at her phone).

After Tetris gameplay, the participant will count aloud for 2 min to measure HRV recovery (Count aloud task B). Finally, the participant will be asked not to play Tetris or to research the therapeutic use of Tetris until she terminated the study.

**Figure 2:**
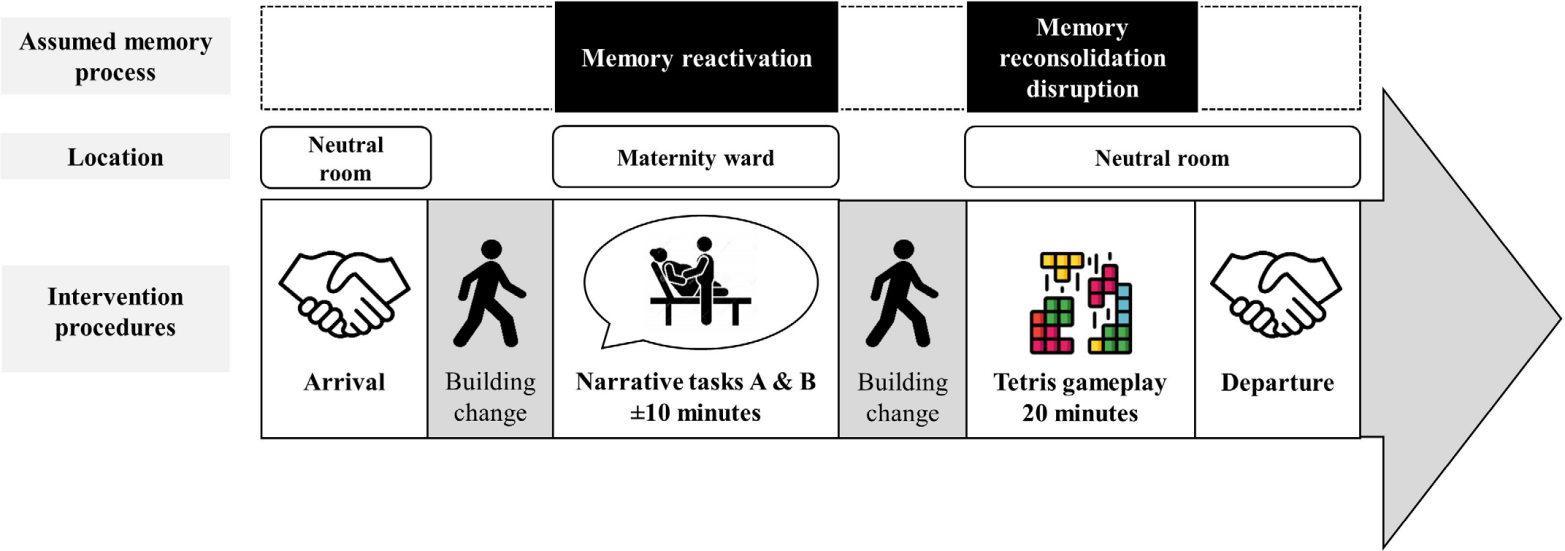
Intervention procedures. Adapted from Deforges *et al*,^27^ with permission.

An overview of the intervention procedures is shown in figure 2.

### Primary outcome

The primary outcome will be the difference in the number of CB-IM between baseline and the 2 weeks postimmediate intervention across groups.

### Secondary outcomes

Secondary outcomes will be:

1. The change in CB-PTSS between: (a) baseline and at 2 weeks postimmediate intervention across groups, (b) baseline and follow-up in the IIG.
2. The change in the number of CB-IM between baseline and follow-up in the IIG.
3. The acceptability of the intervention in both groups.

### Exploratory outcomes

This study will investigate the following exploratory outcomes in both groups:

1. The course of the distress, nowness, sensorial modalities, and content related to CB-IM before and after the intervention.
2. The association of HRV and subjective distress assessed during the different phases of the intervention, with the intervention efficacy (number of CB-IM and CB-PTSS postintervention).

In addition, exploratory outcomes will include the following in the IIG:

1. The change in postnatal depression, and anxiety symptoms between baseline and follow-up.
2. The change in the number of CB-IM between the 2 weeks postimmediate intervention and follow-up.
3. The change in CB-PTSS between 2 weeks postimmediate intervention and follow-up.

We will also explore, in the WCG, the change in the number of CB-IM and CB-PTSS between the 2 weeks predelayed and postdelayed intervention.

### Data collection

Table 1 provides an overview of study outcomes and related timepoints.

**Table 1:** Overview of study outcomes and timepoints

Variables	Instruments	Study period						
		T1 Baseline	T2 Immediate intervention	T3 Postimmediate intervention	T4 Delayed intervention	T5 Postdelayed intervention	T6 Follow-up	T7 Feedback phone call
CB-IM	Diaries	IIG+WCG		IIG+WCG		WCG	IIG	
Content of CB-IM		IIG+WCG		IIG+WCG		WCG	IIG	
Distress of CB-IM		IIG+WCG		IIG+WCG		WCG	IIG	
Nowness of CB-IM		IIG+WCG		IIG+WCG		WCG	IIG	
Sensorial modalities of CB-IM		IIG+WCG		IIG+WCG		WCG	IIG	
CB-PTSS	City Birth Trauma Scale^44^	IIG+WCG		IIG+WCG		WCG	IIG	
Anxiety	Hospital Anxiety and Depression scale–Anxiety subscale^46^	IIG+WCG					IIG	
Postnatal depression	Edinburgh Postnatal Depression Scale^48^	IIG+WCG					IIG	
Heart rate variability	Firstbeat Bodyguard 2 ECG device		IIG		WCG			
Subjective distress	Visual Analogue Scales		IIG		WCG			
Intervention acceptability	Feedback questionnaire III							IIG+WCG
Sociodemographic variables	Sociodemographic questionnaire	IIG+WCG						
Obstetric and medical data	Medical records and self-report questionnaire	IIG+WCG						

CB-IM, childbirth-related intrusive memories; CB-PTSS, childbirth-related post-traumatic stress symptoms; ECG, Electrocardiogram; IIG, Immediate Intervention Group; T1, weeks 1 and 2 preimmediate intervention; T2, day 15; T3, weeks 1 and 2 postimmediate intervention; T4, day 30; T5, weeks 1 and 2 postdelayed intervention; T6, weeks 5 and 6 postimmediate intervention; WCG, waitlist control group.

## Measures

### Childbirth-related post-traumatic stress symptoms

#### Diaries

For each CB-IM, participants will be asked to write in a diary the time it occurred, its content and to report, from 0 = *not at all* to 10 = *extremely*: (1) how distressing it was, (2) to what extent they had the impression that the memory was happening here and now (*nowness*^43^) and (3) its sensorial modalities. CB-IM will be defined to participants as ‘*involuntary memories of the labour or childbirth that come to mind without warning. They can be vivid and emotional, and are often like a mental image, such as a photograph or film. They can involve all the senses (…). They can be very short, elusive and fragmented’.*^27^ Participants will use electronic diaries hosted in REDCap, or a paper version (eg, if not able to use the electronic ones), adapted from previous work.^27^

#### City Birth Trauma Scale (CBTS)

This questionnaire assesses CB-PTSS during the past week, and diagnostic criteria of PTSD according to the DSM-5.^44^ Twenty-two items, using a 4-point Likert scale, assess the four CB-PTSS clusters (*re-experiencing*, *avoidance*, *negative cognitions and mood*, *hyperarousal*). Original instructions of the CBTS will be adapted for participants who had multiple childbirth(s) to complete the questionnaire regarding the index traumatic childbirth. Good psychometric properties were found for the French version of the CBTS.^45^

### Anxiety and depression symptoms

#### Anxiety subscale of the Hospital Anxiety and Depression Scale (HADS-A)

This subscale assesses anxiety symptoms and consists of seven items using 4-point Likert scales.^46^ Good psychometric properties are reported for the French version of the HADS.^47^

#### Edinburgh Postnatal Depression Scale (EPDS)

This 10-item questionnaire measures postnatal depression symptoms during the past week with 4-point Likert scales.^48^ The French version of the EPDS has good psychometric properties.^49^

### Intervention-related measures

#### Intervention acceptability

Participants will answer the Feedback questionnaire III assessing intervention acceptability, usefulness, and expectations regarding its effects. These questions, based on previous work,^27^ have been designed to best match the intervention characteristics.

#### Heart rate variability

Participants’ HRV will be recorded using a Firstbeat Bodyguard 2 (Firstbeat Technologies, Jyväskylä, Finland) ECG device during the whole intervention procedures. Count aloud tasks A and B will measure HRV baseline and recovery, in order to compare participants HRV during Narrative tasks A and B, and Tetris gameplay.

#### Subjective distress

Visual analogue scales will be presented to participants at 11 timepoints during the intervention to assess their subjective distress level from 0 = *not stressed and/or anxious at all* to 10 = *extremely stressed and/or anxious*.

### Sociodemographic and obstetric characteristics

#### Sociodemographic variables

A sociodemographic questionnaire will assess participants’ age, nationality, marital status, education level and trauma history.

#### Obstetric and medical data

Obstetric and medical data will be extracted from participants’ medical record, such as childbirth mode, gestational age and Apgar scores. Birth-related information will also be collected at baseline, through a self-report questionnaire (eg, presence of a relative, perceived threat for self or infant).

### Other variables

This study includes other outcomes, aiming to specify the effects of the intervention, presented in table 2.

**Table 2:** Other trial outcomes, including instruments and time points

Variables	Instruments	Timepoints	Description
Cognitive flexibility	Cognitive flexibility scale^50^	T1	Cognitive flexibility defines, inter alia, the capacity to adapt to the situation and being flexible.^50^
Cue specificity	Feedback questionnaires I and III	T2/T4, T7	These questionnaires will assess the similarity between childbirth narration and the original childbirth memory.
Context specificity	Feedback questionnaires II and III	T3/T5, T7	Questions will assess the similarity between the intervention context and the original traumatic context.
Diary compliance	Diaries	T1, T3, T5, T6	Diary compliance will check the extent to which participants were able/remembered to report their CB-IM at the end of each diary.
HRV control variables	Questionnaire on HRV control variables	T1, T2/T4	This questionnaire will contain control variables regarding the analyses of the HRV (eg, weight, height, medicine use, cigarettes and alcohol consumption).
Video games use	Feedback questionnaires I and III	T2/T4, T7	Questions will assess participants’ video games use (eg, engagement in the game during the intervention, Tetris perceived difficulty, video games habits, and Tetris use postintervention).
Sleep	Sleep questionnaire	T3/T5	This questionnaire will evaluate the number of hours of sleep during the 24 hours following the intervention.
Concurrent psychological treatment	Feedback questionnaire III	T7	A question will check the absence of a concurrent psychological treatment in relation to the index childbirth during study participation.

CB-IM, childbirth-related intrusive memories; HRV, heart rate variability; T1, weeks 1 and 2 preimmediate intervention; T2, day 15; T3, weeks 1 and 2 postimmediate intervention; T4, day 30; T5, weeks 1 and 2 postdelayed intervention; T6, weeks 5 and 6 postimmediate intervention; T7, feedback phone call.

### Sample size calculation

A sample size calculation was conducted for our primary and secondary outcomes based on the results obtained from the pilot study,^27^ assessing the efficacy of the same intervention using a single-group pre–post design, and from laboratory studies using RCT designs to assess the efficacy of a similar BI-VT.^23 24^ A sample size of 90 participants (45 allocated in each group) is estimated to be necessary to detect the effect of the intervention on our primary and secondary outcomes with an 80% power (α = 0.05). Based on our experience with this study population,^11 27^ a conservative estimate of a 30% drop-out and exclusion was assumed. We therefore plan to recruit 120 participants in total.

### Data management and statistical analysis

Participants’ entries in electronic diaries and questionnaires will be directly stored in the REDCap software. The research staff will enter manually the rest of the data collected during the screening, the intervention and paper diaries in REDCap. All data will be hosted on a high security server of the Lausanne University Hospital performing automatic continuous backups. All primary outcome data and a random 5% of the rest of the data will be double-checked. Independent and blind coders will inspect diary entries to detect any non-compliance with instructions and count the number of CB-IM. A blind statistician will perform the analyses. To ensure blinding of the coders and the statisticians, they will receive a dataset not indicating which allocation group corresponds to the IIG or WCG. Only the study team will have access to the full coded trial dataset.

For primary outcomes, group differences regarding change of the number of CB-IM between baseline and the 2 weeks postimmediate intervention will be analysed using repeated-measures analyses (intention-to-treat analyses). For the secondary outcomes, repeated-measures analyses will be performed to analyse group differences concerning CB-PTSS between baseline and at 2 weeks postimmediate intervention. Logistic regression analyses will also compare the proportion of participants meeting the diagnostic criteria for CB-PTSD across time and groups. Furthermore, repeated measures analyses will serve to analyse change in the number of CB-IM and CB-PTSS between baseline and follow-up in the IIG. Group differences regarding the proportion of participants meeting the diagnostic criteria for CB-PTSD will be analysed with logistic regression analyses. The mean score of the intervention acceptability will be calculated across both groups. Additional post-hoc analyses conducted will be described as such in publications. Missing data will be dealt with using multiple imputation procedures, if appropriate.

### Safety

Any serious adverse event occurring during data collection will be documented and reported to the competent ethics committee.

### Patient and public involvement

Participants will evaluate the intervention acceptability and will be able to give feedback on the intervention. Each participant will receive a personalised debriefing session. Study results will be sent to participants and shared with the public via social media and public events.

## Ethics and dissemination

Ethical approval of this protocol (dated 1 December 2022) was granted by the Human Research Ethics Committee of the Canton de Vaud (study number 2022-00652). The study sponsor is the Bureau du Promoteur de Recherche of the Lausanne University Hospital. Participants will provide an informed consent prior to study participation (see online supplemental material). The study will be monitored by an independent and certified data monitoring body. An initiation, intermediate and a closing visit will be realised on site by the independent study monitor. A scientific steering committee is consulted regularly for scientific decisions regarding the conduct of the study. This protocol was registered at ClinicalTrials.gov (NCT05381155) prior to data collection, in May 2022. Results of this study will be submitted for publication to peer-reviewed journals and presented in relevant conferences to ensure dissemination in the scientific community. Furthermore, data will be shared in an anonymised form, at the time of the publication of the related outcomes, in an open access register.

10.1136/bmjopen-2023-073874.supp1 Supplementary data

## Supplementary Material

Reviewer comments

Author's manuscript
