# Supplementary material for: Tackling childbirth-related intrusive memories with a single-session behavioural intervention involving a visuospatial task: protocol for a single-blind, waitlist-controlled randomised trial
Source: BMJ Open. 2023 May 29;13(5):e073874. doi: 10.1136/bmjopen-2023-073874 (PMC10230990; doi:10.1136/bmjopen-2023-073874)
Supplement: Supplementary data [file bmjopen-2023-073874supp001.pdf]

Unil | Université de Lausanne
Institut universitaire de formation et de recherche en soins FBM
CHUV
FNS SNF
Fonds national suisse
Schweizerischer Nationalfonds
Fondo nazionale svizzero
Swiss National Science Foundation

Feuille d'information

Étude ASTRAL : Lutter contre les souvenirs intrusifs après un accouchement difficile

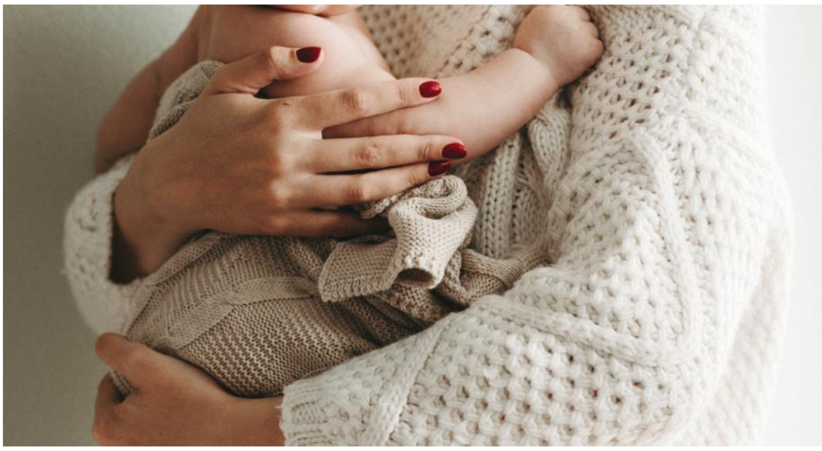

Chère Madame,

Merci beaucoup pour votre intérêt pour notre projet de recherche ! Comme expliqué au téléphone, nous aimerions vous proposer de participer à notre projet de recherche. Ce projet est développé au sein du groupe de recherche périnatale de Lausanne, qui est rattaché au CHUV et à l'Université de Lausanne. Ce feuillet d'information décrit le projet de recherche de façon détaillée. Avant de décider d'y participer, il est important que vous compreniez le but de cette recherche, ainsi que ses enjeux et ce que cela implique pour vous. C'est pourquoi nous vous prions de parcourir attentivement ce document. Votre participation est entièrement libre et toutes les données collectées dans le cadre de ce projet sont soumises à des règles strictes en matière de protection des données. Une fois l'étude terminée, nous vous en communiquerons les résultats si vous le souhaitez.

Pour vous proposer d'ores et déjà un aperçu du projet, voici les points clés à retenir. Vous trouverez à la suite des informations complémentaires plus détaillées.

Pourquoi menons-nous ce projet de recherche ?

Il peut arriver qu'un accouchement soit vécu difficilement, et entraîne des « flashbacks » ou « souvenirs intrusifs ». Il s'agit de souvenirs récurrents et involontaires de la naissance, qui sont parfois source de détresse et peuvent diminuer le bien-être maternel. Avec cette étude, nous souhaiterions savoir si une activité simple sur console, précédée par une brève évocation du vécu de l'accouchement, réduit efficacement le nombre de souvenirs intrusifs liées à cet évènement. Notez que l'évocation de l'accouchement dure environ 10 minutes et qu'elle est superficielle : il ne s'agit en aucun cas de revivre la naissance ou d'entrer dans les détails, et vous êtes libre de ce que vous souhaitez partager.

Que dois-je faire si j'accepte de participer ?

- Si vous acceptez de participer à notre projet, vous devrez signer ce formulaire, venir réaliser l'activité au CHUV et compléter divers questionnaires aux périodes suivantes :
    1. **Avant le rendez-vous**, vous remplirez un journal de bord dans lequel vous reporterez quotidiennement vos souvenirs intrusifs. Le journal de bord est à compléter sur votre smartphone grâce à l'application sécurisée MyCap. Une version papier du journal de bord est également disponible, mais nous vous encourageons à utiliser la version électronique

Fort D, et al. BMJ Open 2023; 13:e073874. doi: 10.1136/bmjopen-2023-073874

en raison de sa practicité. Vous recevrez également par mail une série de questionnaires, qui prennent environ 15 min à remplir.

2. **Lors d'un rendez-vous** d'environ 1h30 au CHUV, vous réaliserez l'activité et répondrez à de brefs questionnaires. Votre fréquence cardiaque sera également relevée à l'aide d'un simple capteur.
3. **Après le rendez-vous**, vous reporterez à nouveau quotidiennement vos souvenirs intrusifs dans un journal de bord, et vous répondrez à quelques questionnaires envoyés par mail, prenant environ 10 min.
4. L'étude se clôture par un bilan, au téléphone, avec un bref questionnaire et un débriefing sur votre participation. Nous proposerons également à certaines participantes de réaliser, au même moment, un entretien au sujet du vécu de leur participation à l'étude.

- Les participantes sont réparties au hasard dans deux groupes qui réaliseront tous les deux l'activité à visée thérapeutique et complèront les journaux de bord sur une durée de 6 semaines. Les deux groupes se différencient concernant l'ordre dans lequel ils remplissent leurs journaux de bord et réalisent l'activité.
- Les informations récoltées dans les journaux de bord permettront de préparer l'activité à visée thérapeutique, de façon à l'adapter au mieux à votre situation et à vos besoins. Elles permettront également de mesurer les bénéfices de l'activité, en les comparant aux souvenirs intrusifs reportés après l'activité.
- La participation à l'étude dure environ 2 mois. Cette durée comprend la complétion des journaux et des questionnaires, le rendez-vous au CHUV et l'entretien téléphonique de fin d'étude.

Quels sont les bénéfices et les risques liés à la participation au projet ?

Bénéfices pour les participants

- La participation à ce projet pourrait potentiellement vous apporter un bénéfice direct, en diminuant le nombre de vos souvenirs intrusifs liés à votre accouchemenent.
- En faisant avancer la recherche scientifique, vous contribuez également au développement d'une technique thérapeutique qui pourrait aider les autres femmes ayant des souvenirs intrusifs à la suite de leur accouchement.

Risques et contraintes

- À notre connaissance, cette étude ne comporte aucun risque particulier pour les participantes. Le rendez-vous au CHUV peut entraîner un inconfort passager du fait de la brève évocation du vécu de l'accouchement.
- Lors de l'appel téléphonique de fin d'étude, si l'activité n'a pas eu d'effet positif pour vous ou que votre vécu de l'accouchement vous fait toujours souffrir, la coordinatrice de l'étude pourra vous indiquer à quel·le·s professionnel·le·s de la périnatalité vous pouvez vous adresser et vous apporter davantage d'information sur les effets psychiques que peuvent entraîner une naissance traumatique.

En apposant votre signature à la fin du document, vous certifiez en avoir compris tout le contenu et consentir librement à prendre part au projet.

Fort D, *et al. BMJ Open* 2023; 13:e073874. doi: 10.1136/bmjopen-2023-073874

Unil | UNIL | Université de Lausanne Institut universitaire de formation et de recherche en soins FBM — CHUV — FNSNF Fonds national suisse Schweizerischer Nationalfonds Fondo nazionale svizzero Swiss National Science Foundation

Feuille d'information

Information détaillée

1. Objectif du projet et sélection des participantes

Dans cette feuille d'information, notre projet de recherche est aussi simplement désigné par le terme *étude*. Si vous acceptez d'y prendre part, vous êtes *une participante à l'étude*.

Il peut arriver qu'un accouchement soit vécu difficilement, et entraîne des « flashbacks » ou « souvenirs intrusifs ». Il s'agit de souvenirs récurrents et involontaires de la naissance, qui sont parfois source de détresse et peuvent diminuer le bien-être maternel. Avec cette étude, nous souhaiterions savoir si une activité simple sur console, précédée par une brève évocation du vécu de l'accouchement, réduit efficacement le nombre de souvenirs intrusifs liées à cet évènement. Notez que l'évocation de l'accouchement dure environ 10 minutes et qu'elle est superficielle : il ne s'agit en aucun cas de revivre la naissance ou de rentrer dans les détails, et vous êtes libre de ce que vous souhaitez partager.

La participation est ouverte aux femmes ayant accouché au CHUV dès la 6ème semaine postpartum et qui nous ont donné leur consentement écrit. Afin de pouvoir observer une potentielle amélioration à la suite de la réalisation de l'activité, nous incluons dans l'étude les femmes rapportant des souvenirs intrusifs réguliers liés à leur accouchement. Si vous n'êtes pas sûre du nombre de vos souvenirs intrusifs, nous vous proposerons de remplir un premier journal de bord afin d'évaluer leur nombre. Si vous n'avez pas rapportez de souvenirs intrusifs réguliers à la fin de celui-ci, nous vous proposerons tout de même de réaliser l'activité bien que vos données ne pourront pas être utilisées dans le cadre de notre étude.

La participation à l'étude concerne les femmes ayant un niveau de français suffisant pour compléter les différentes étapes de l'étude, qui ne prennent pas de médicament contenant du propranolol et qui ne souffrent pas, elles et leur(s) enfant(s), d'une maladie sévère. Les femmes étant dans l'incapacité de discerner leurs souvenirs intrusifs liés à leur accouchement de souvenirs intrusifs liés à d'autres événements, ou ayant des antécédents d'abus d'alcool ou de drogues ne peuvent pas prendre part à cette étude. En effet, nous pensons que ces expériences doivent être étudiées de façon séparée. Pour les mêmes raisons, les femmes suivant un traitement psychologique en lien avec leur accouchement au moment du recrutement ne sont pas invitées à participer à l'étude.

2. Informations générales sur le projet

Cette étude est monocentrique car elle est conduite dans un seul centre de recherche, à Lausanne. Cette étude mobilisera environ 90 femmes au total. Votre participation à l'étude sera d'environ 2 mois. L'activité, qui dure environ 1h30, se déroulera au CHUV avec une psychologue. Si vous participez à l'étude, vous serez affectée au hasard dans un des deux groupes qui réaliseront tout deux l'activité à visée thérapeutique. Ces groupes se différencient uniquement concernant l'ordre de réalisation des journaux de bord et de l'activité (le déroulé des étapes est décrit en détail au point 3.). Afin de préparer le rendez-vous au CHUV, il sera demandé aux participantes de remplir un journal de bord dans lequel elles rapporteront leurs souvenirs intrusifs liés à leur accouchement, avant puis après l'activité.

Cette étude est réalisée dans le respect des prescriptions de la législation suisse. Nous suivons en outre l'ensemble des directives reconnues au niveau international. La commission d'éthique compétente a examiné et autorisé l'étude. Vous trouverez également un descriptif de l'étude sur le site Internet de l'Office fédéral de la santé publique (www.kofam.ch) (SNCTP000004935) et sur ClinicalTrials.gov.

3. Déroulement pour les participantes

- Si vous acceptez de participer, vous signerez le formulaire de consentement à la fin de ce document. Vous devrez ensuite réaliser les étapes rapportées ci-dessous :

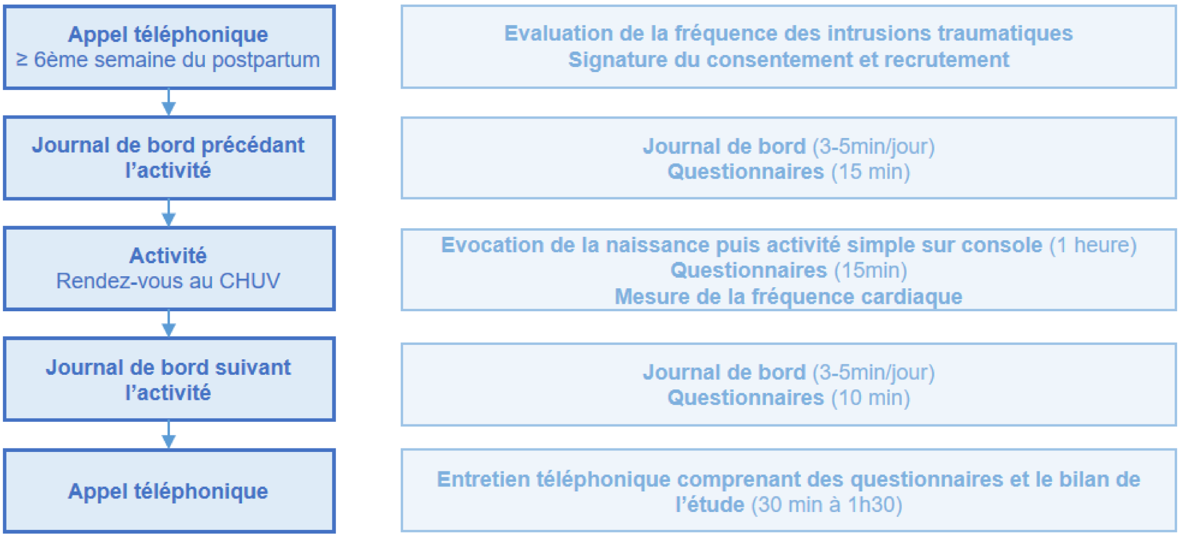

- Au minimum 6 semaines après votre accouchement, vous recevrez un lien vers des questionnaires en ligne (10 minutes). Nous vous demanderons ensuite de reporter dans un journal de bord vos souvenirs intrusifs (3 à 5 minutes/jour). Nous vous enverrons des questionnaires en ligne à différents moments du journal (5 minutes).
- À la fin du premier journal, vous aurez rendez-vous au CHUV. Il commencera par une brève évocation de votre accouchement, pendant 10 minutes. Ensuite, vous ferez une activité simple sur console, durant 20 minutes. Celle-ci n'a pas de rapport avec votre accouchement, elle ne nécessite aucune compétence particulière. Vous devrez également répondre à de brefs questionnaires (5 minutes), et votre fréquence cardiaque sera mesurée lors du rendez-vous grâce à un dispositif non-invasif.
- À la suite du rendez-vous, vous remplirez de nouveau un journal de bord et répondrez à un bref questionnaire (5 minutes).
- Un mois après le rendez-vous, vous recevrez un questionnaire en ligne (5 minutes).
- À la fin du dernier journal, suite à un bref questionnaire, nous ferons le bilan de l'étude et de l'évolution de vos souvenirs intrusifs, lors d'un dernier appel téléphonique durant environ 30 minutes. Nous proposerons également à certaines participantes de réaliser un entretien, lors de cet appel, au sujet du vécu de leur participation à l'étude et des potentiels bénéfices qu'elles ont observés à la suite du rendez-vous. Cet entretien prend environ 1 heure et a pour objectif de permettre à l'équipe de recherche de mieux comprendre votre ressenti de l'étude. Son contenu audio sera enregistré sur un serveur sécurisé puis supprimé une fois que les données seront retranscrites et codées. Si la participation à cet entretien vous intéresse, vous pouvez nous le signaler en cochant la case correspondante à la fin de ce feuillet. Si vous ne souhaitez pas y prendre part, cela n'affectera pas le reste de votre participation à l'étude.

4. Bénéfices pour les participantes

Si vous participez à l'étude, cela pourrait potentiellement diminuer le nombre de vos souvenirs intrusifs en lien avec votre accouchement. En effet, plusieurs études précédentes ont suggéré que cette activité que nous testons pourrait les réduire, immédiatement après un événement difficile ou même plusieurs mois voire années après celui-ci. Si l'activité est sans effet ou que votre vécu de l'accouchement vous fait encore souffrir, la coordinatrice de l'étude pourra vous indiquer à quel·le·s professionnel·le·s de la périnatalité vous pouvez vous adresser, et vous apporter davantage d'information sur les conséquences psychiques qu'une naissance traumatique peut avoir. Par ailleurs, les résultats de l'étude pourraient se révéler importants par la suite, pour d'autres femmes ayant également des souvenirs intrusifs suite à leur accouchement.

Fort D, *et al. BMJ Open* 2023; 13:e073874. doi: 10.1136/bmjopen-2023-073874

5. Caractère facultatif de la participation et obligations

Votre participation est entièrement libre. Si vous choisissez de ne pas participer ou si vous choisissez de participer et revenez sur votre décision pendant le déroulement de l'étude, vous n'aurez pas à vous justifier. Cela ne changera rien à votre prise en charge médicale habituelle. Vous pouvez à tout moment poser des questions sur l'étude à la Prof. Antje Horsch ou à Mme Déborah Fort (cf. coordonnées à la fin du document).

En tant que participante à l'étude, vous serez tenue de suivre les instructions liées à la procédure de l'étude et de remplir les journaux de bord et les questionnaires, ainsi que de vous présenter lors du rendez-vous. En cas d'augmentation de la fréquence de vos souvenirs intrusifs ou si vous débutez un traitement ou une thérapie en lien avec votre vécu de l'accouchement, nous vous prierons d'en informer la Prof. Antje Horsch ou Mme Déborah Fort.

6. Risques et contraintes pour les participantes

À notre connaissance, cette étude ne comporte aucun risque particulier pour les participantes. Le rendez-vous au CHUV peut entraîner un inconfort passager du fait de la brève évocation de l'accouchement.

7. Alternatives

Si vous décidez de ne pas prendre part à l'étude, il sera possible de vous adresser à un·e psychothérapeute connaissant les troubles psychiques liés aux expériences difficiles. Si vous refusez de participer à cette étude, ou décidez d'interrompre votre participation, cela n'aura aucune conséquence sur votre suivi médical.

8. Résultats

L'étude permet d'obtenir différents résultats :

1. Des résultats individuels qui vous concernent directement (p. ex. comment vos souvenirs intrusifs ont évolué suite à l'activité),
2. Les résultats globaux de l'étude (p. ex. comment les souvenirs intrusifs de l'ensemble des participantes ont évolué suite à l'activité).

L'investigatrice vous avisera, pendant l'étude, de toute nouvelle découverte importante vous concernant. Vous serez informée oralement et par écrit ; vous pourrez par la suite à nouveau décider si vous souhaitez poursuivre votre participation à l'étude. Si vous ne souhaitez pas être mise au courant de ces éventualités, merci de l'indiquer aux personnes responsables de l'étude.

L'investigatrice peut vous faire parvenir, à l'issue de l'étude, une synthèse des résultats globaux si vous le souhaitez.

9. Confidentialité des données

9.1. Traitement et codage des données

Dans le cadre de cette étude, des données relatives à votre personne et à votre santé sont codées au moment du relevé. Le codage signifie que toutes les données permettant de vous identifier (nom, date de naissance, etc.) sont remplacées par un code. Il n'est pas possible de relier les données à votre personne sans le code, qui restent en permanence au sein des dossiers de l'équipe de recherche, stockées sur les serveurs sécurisés du CHUV.

Seul un nombre limité de personnes peut consulter vos données sous une forme non codée, et ce, exclusivement afin de pouvoir accomplir des tâches nécessaires au déroulement de l'étude. Ces personnes sont tenues au secret professionnel. En tant que participante, vous avez le droit de consulter vos données.

9.2. Protection des données

Toutes les directives relatives à la protection des données sont rigoureusement respectées.

Les données à l'origine des publications qui découleront de cette étude vont être publiées sur un site dédié ('Open Data') sous une forme codée (https://zenodo.org/). Dans le cadre d'un tel partage, vos données seront toujours codées de façon qu'il ne soit pas possible de remonter à votre identité. Cette démarche de partage des données permet la validation des résultats publiés (reproductibilité), de cumuler les données de différentes recherches et plus généralement l'utilisation des données par d'autres chercheurs. En cas de retrait de votre consentement à participer à ce projet, les données déjà partagées ne pourront pas être retirées. Vous devez être d'accord avec cela si vous participez à l'étude.

9.3. Droit de consultation dans le cadre d'inspections

L'étude peut faire l'objet d'inspections. Celles-ci peuvent être effectuées par la commission d'éthique compétente ou par le promoteur qui a initié l'étude. L'investigatrice doit alors communiquer vos données pour les besoins de ces inspections. Toutes les personnes impliquées sont tenues au plus strict secret professionnel.

10. Retrait du projet

Vous pouvez à tout moment vous retirer de l'étude si vous le souhaitez. Cependant, les données jusque-là pourront encore être analysés sous forme codée. En cas de retrait, vos données continuent de figurer sous forme codée dans les documents de l'étude. Vous devez donc être d'accord avec cela avant de donner votre consentement.

11. Dédommagement

Les dépenses liées aux frais de transport (transports publics ou tickets de parking) qui découlent directement de la participation à l'étude vous seront remboursées sous présentation des documents originaux. Votre participation n'aura aucune conséquence financière pour vous ou votre assurance maladie.

12. Responsabilité

Le CHUV qui a initié le projet de recherche et est chargé de sa réalisation, est responsable des dommages que vous pourriez subir en relation avec les activités de recherche. Les conditions et la procédure sont fixées par la loi. Bien que ces recherches ne présentent pas de risque prévisible, l'institution (le promoteur) est responsable, en vertu des dispositions légales, de tout dommage pouvant survenir dans le cadre de l'étude. Si vous subissiez un dommage du fait de votre participation à l'étude, il vous faudrait vous adresser à l'investigatrice mentionnée ci-avant.

13. Financement

L'étude est financée par le Fond National pour la recherche Suisse (SNF IZCOZ0_205434/1).

14. Interlocutrices

Vous pouvez à tout moment poser des questions au sujet de l'étude. En cas de doutes, de craintes ou d'urgences pendant ou après l'étude, vous pouvez vous adresser à l'une des interlocutrices suivantes :

Feuille d'information

Prof. Antje Horsch : Directrice de l'étude

Institut Universitaire de Formation et Recherche en Soins, Université de Lausanne et Département Femme-Mère-Enfant, CHUV.
Route de la Corniche 10
Biopôle 2
1010 Lausanne
antje.horsch@chuv.ch
079/556.07.50

Mme Déborah Fort : Doctorante et coordinatrice de l'étude

Institut Universitaire de Formation et Recherche en Soins, Université de Lausanne
Route de la Corniche 10
Biopôle 2
1010 Lausanne
deborah.fort@chuv.ch
079/556.73.37

Fort D, *et al. BMJ Open* 2023; 13:e073874. doi: 10.1136/bmjopen-2023-073874

Unil | Université de Lausanne Institut universitaire de formation et de recherche en soins FBM — CHUV — FNS SNF Fonds national suisse Schweizerischer Nationalfonds Fondo nazionale svizzero Swiss National Science Foundation

Feuille d'information

Déclaration de consentement

Déclaration de consentement écrite pour la participation à une étude clinique
Veuillez lire attentivement ce formulaire. N'hésitez pas à poser des questions lorsque vous ne comprenez pas quelque chose ou que vous souhaitez avoir des précisions. Votre consentement écrit est nécessaire pour participer au projet.

**Numéro BASEC du projet de recherche :**	2022-00652
**Titre (scientifique et usuel) :**	*tAckling intruSive Traumatic memoRies After chiLdbirth (ASTRAL): A single-blinded waitlist randomized controlled trial*<br>Etude ASTRAL : Lutter contre les souvenirs intrusifs après un accouchement difficile
**Institution responsable (promoteur et adresse complète) :**	Prof. Antje Horsch<br>Institut Universitaire de Formation et Recherche en Soins, Université de Lausanne et CHUV, Biopôle 2. Route de la Corniche 10 - 1010 Lausanne
**Lieu de réalisation :**	CHUV
**Responsables sur le site :**<br>Nom et prénom en caractères d'imprimerie :	Prof. Antje Horsch
**Participant / Participante :**<br>Nom et prénom en caractères d'imprimerie :<br>Date de naissance :	____________________<br>____________________

- Je déclare avoir été informée, par la responsable soussignée, oralement et par écrit, des objectifs et du déroulement de l'étude ainsi que des avantages et des inconvénients possibles et des risques éventuels.
- Je prends part à cette étude de façon volontaire et j'accepte le contenu de la feuille d'information qui m'a été remise. J'ai eu suffisamment de temps pour prendre ma décision.
- J'ai reçu les réponses aux questions que j'ai posées en relation avec ma participation à cette étude. Je conserve la feuille d'information et reçois une copie de ma déclaration de consentement écrite.
- J'ai été informée de l'existence possible d'autres traitements et thérapies.
- J'accepte que les spécialistes compétents du promoteur de ce projet, de la commission d'éthique compétente puissent consulter mes données non codées afin de procéder à des contrôles et des inspections, à condition toutefois que la confidentialité de ces données soit strictement assurée.
- Je serai informée des résultats ayant une incidence directe sur ma santé. Si je ne souhaite pas obtenir ces informations, je prends contact avec la directrice de l'étude.
- Je sais que j'ai à tout moment le droit de consulter mes propres données.
- Je sais que mes données personnelles peuvent être transmises à des fins de recherche dans le cadre de ce projet uniquement et sous une forme codée.
- Je suis d'accord pour que mes données dûment codées soient partagées à des fins de recherche en accès libre sur des bases de données biomédicales.
- J'ai compris que mes données codées seront partagées en open data et qu'en cas de retrait de mon consentement, celles-ci ne pourront pas être retirées.
- Je peux, à tout moment et sans avoir à me justifier, révoquer mon consentement à participer à l'étude, sans que cette décision n'ait de répercussions défavorables sur la suite de ma prise en charge. Les données qui ont été recueillies jusque-là seront cependant analysées dans le cadre de l'étude.
- Je suis informée que l'assurance-responsabilité civile du CHUV couvre les dommages éventuels imputables au projet.
- Je suis consciente que les obligations mentionnées dans la feuille d'information destinée aux participantes doivent être respectées pendant toute la durée de l'étude. La directrice de l'étude peut m'exclure à tout moment de l'étude dans l'intérêt de ma santé.
- Je suis d'accord pour être recontactée par les membres de l'équipe de recherche pour de futures études. Il reste entendu que je peux révoquer en tout temps cette autorisation. Si je ne suis pas d'accord, je peux toujours participer à l'étude.

Fort D, *et al. BMJ Open* 2023; 13:e073874. doi: 10.1136/bmjopen-2023-073874

Merci de cocher la case suivante si vous êtes intéressée à participer à l'entretien de fin d'étude :

- ☐ Je suis d'accord pour que l'appel audio effectué lors de l'entretien de fin d'étude soit enregistré afin de permettre à l'équipe de recherche de mieux comprendre mon ressenti sur l'étude.

Lieu, date<br>__________________________	Signature du participant / de la participante<br>____

Attestation de la personne investigatrice : Par la présente, j'atteste avoir expliqué au participant/à la participante la nature, l'importance et la portée de l'étude. Je déclare satisfaire à toutes les obligations en relation avec cette étude conformément au droit suisse en vigueur. Si je devais prendre connaissance, à quelque moment que ce soit durant la réalisation de l'étude, d'éléments susceptibles d'influer sur le consentement du participant/de la participante à prendre part au projet, je m'engage à l'en informer immédiatement.

Lieu, date<br>__________________________	Nom et prénom de la personne investigatrice en caractères d'imprimerie.<br>____<br>Signature de la personne investigatrice<br>____

Fort D, *et al. BMJ Open* 2023; 13:e073874. doi: 10.1136/bmjopen-2023-073874
