# Supplementary material for: Tackling childbirth-related intrusive memories with a single-session behavioural intervention involving a visuospatial task: protocol for a single-blind, waitlist-controlled randomised trial
Source: BMJ Open. 2023 May 29;13(5):e073874. doi: 10.1136/bmjopen-2023-073874 (PMC10230990; doi:10.1136/bmjopen-2023-073874)
Supplement: Author's manuscript [file bmjopen-2023-073874.draft_revisions.pdf]

BMJ Open is committed to open peer review. As part of this commitment we make the peer review history of every article we publish publicly available.

When an article is published we post the peer reviewers' comments and the authors' responses online. We also post the versions of the paper that were used during peer review. These are the versions that the peer review comments apply to.

The versions of the paper that follow are the versions that were submitted during the peer review process. They are not the versions of record or the final published versions. They should not be cited or distributed as the published version of this manuscript.

BMJ Open is an open access journal and the full, final, typeset and author-corrected version of record of the manuscript is available on our site with no access controls, subscription charges or pay-per-view fees (http://bmjopen.bmj.com).

If you have any questions on BMJ Open's open peer review process please email info.bmjopen@bmj.com

BMJ Open

Tackling childbirth-related intrusive memories with a single-session behavioural intervention including a visuospatial task: protocol of a single-blind waitlist randomised controlled trial

Journal:	*BMJ Open*
Manuscript ID	bmjopen-2023-073874
Article Type:	Protocol
Date Submitted by the Author:	21-Mar-2023
Complete List of Authors:	Fort, Déborah; University of Lausanne Faculty of Biology and Medicine, Institute of Higher Education and Research in Healthcare<br>Deforges, Camille; University of Lausanne Faculty of Biology and Medicine, Institute of Higher Education and Research in Healthcare<br>Messerli-Bürgy, Nadine; University of Lausanne Faculty of Social and Political Science, Family and Development research center (FADO)<br>Michael, Tanja; Saarland University, Division of Clinical Psychology and Psychotherapy, Department of Psychology<br>Baud, David; Lausanne University Hospital Department of Woman Mother and Child, Materno-Fetal and Obstetrics Research Unit<br>Lalor, Joan; University of Dublin Trinity College, School of Nursing and Midwifery<br>Rimmele, Ulrike; University of Geneva, Emotion and Memory Laboratory, Faculty of Education Sciences and Psychology; University of Geneva, Centre interfacultaire de gérontologie et d'études des vulnérabilités (CIGEV)<br>Horsch, Antje; University of Lausanne Faculty of Biology and Medicine, Institute of Higher Education and Research in Healthcare; Lausanne University Hospital Department of Woman Mother and Child, Neonatology Service
Keywords:	Postpartum Period, Adult psychiatry < PSYCHIATRY, Clinical Trial, Life Change Events, Postpartum Women < Postpartum Period

SCHOLARONE™
Manuscripts

CATEGORY

Protocol

TITLE

Tackling childbirth-related intrusive memories with a single-session behavioural intervention including a visuospatial task: protocol of a single-blind waitlist randomised controlled trial

AUTHORS

Déborah Fort, MSc[1]

Camille Deforges, PhD[1]

Nadine Messerli-Bürgy, PhD[2]

Tanja Michael, PhD[3]

David Baud, MD-PhD[4]

Joan Lalor, PhD[5]

Ulrike Rimmele, PhD[6,7,8,9]

Antje Horsch, PhD[1,10]

AFFILIATION

[1] Institute of Higher Education and Research in Healthcare, Faculty of Biology and Medicine, University of Lausanne, Lausanne, Switzerland

[2] Family and Development research center (FADO), Faculty of Social and Political Sciences, University of Lausanne, Lausanne, Switzerland

[3] Division of Clinical Psychology and Psychotherapy, Saarland University, Saarbrücken, Germany

[4] Materno-Fetal and Obstetrics Research Unit, Department Woman-Mother-Child, Lausanne University Hospital, Lausanne, Switzerland

[5] School of Nursing and Midwifery, Trinity College Dublin, Dublin 2, Ireland

For peer review only

[6] Emotion and Memory Laboratory, Faculty of Education Sciences and Psychology, University of Geneva, Geneva, Switzerland

[7] Centre interfacultaire de gérontologie et d'études des vulnérabilités (CIGEV), University of Geneva, Switzerland

[8] Centre interfacultaire en sciences affectives (CISA), University of Geneva, Switzerland

[9] Geneva University Neurocenter, Switzerland

[10] Neonatology Service, Department Woman-Mother-Child, Lausanne University Hospital, Lausanne, Switzerland

CORRESPONDANCE

Antje Horsch

Institute of Higher Education and Research in Healthcare, Faculty of Biology and Medicine, University of Lausanne, Route de la Corniche 10, CH-1010 Lausanne, Switzerland

E-mail: antje.horsch@chuv.ch

WORD COUNT: 3965

For peer review only

ABSTRACT

Introduction

Approximately 12.3% of mothers experience childbirth-related posttraumatic stress symptoms (CB-PTSS). However, evidence as to how to treat CB-PTSS is currently limited. Developing evidence-based interventions is therefore essential. Intrusive memories (IM), a key symptom of CB-PTSS, are distressing and can maintain or trigger other PTSS by reliving the traumatic event. Emerging evidence shows that a behavioural intervention involving a visuospatial task (BI-VT) can reduce the number of IM and severity of PTSS, supposedly by interfering with the reconsolidation of the trauma memory. This study aims to test the efficacy of a single-session BI-VT targeting IM to reduce the number of childbirth-related (CB-) IM and PTSS, in comparison to a waitlist control group (WCG).

Methods and analysis

In this multicenter single-blind randomised controlled trial (RCT), 45 participants will be allocated to the immediate intervention group (IIG), receiving the immediate intervention on day 15, and 45 participants to the WCG receiving the same intervention on day 30 (delayed intervention). All participants will report their CB-IM during the two weeks pre- and post-immediate intervention by using diaries. The IIG will additionally report their CB-IM over weeks five and six post- immediate intervention. Self-report questionnaires will assess CB-PTSS at two weeks pre- and post-immediate intervention in both groups, and at six weeks post-immediate intervention in the IIG. A feedback questionnaire will evaluate the intervention acceptability. The primary outcome will be group differences in the number of CB-IM between the two weeks pre- and post-immediate intervention. Secondary outcomes will, inter alia, include CB-PTSS at two and six weeks post-immediate intervention, changes of CB-IM at weeks five and six post-immediate intervention, and intervention acceptability.

Ethics and dissemination

Ethical approval was granted by the Human Research Ethics Committee of the Canton of Vaud (study number 2022-00652). Results will be presented in peer-reviewed journals and at relevant conferences.

Trial registration number NCT05381155

STRENGTHS AND LIMITATIONS OF THE STUDY

- This study aims to contribute to the development of an innovative, single-session, and easily accessible intervention to reduce CB-PTSS.

For peer review only

- A pilot study has already shown high efficacy and acceptability of this single-session intervention.
- CB-PTSS will be assessed with a validated questionnaire specifically developed for the perinatal context.
- Participants will be blind to group allocation, but the research team will not be.
- The waitlist control group is not an active comparator.

KEYWORDS Posttraumatic stress disorder, Childbirth, Behavioural therapy, Memory reconsolidation, Intrusive memories, Intrusions, Visuospatial task, Tetris.

For peer review only

INTRODUCTION

Childbirth-related post-traumatic stress disorder and intrusive memories

Approximately 12.3% of mothers develop childbirth-related post-traumatic stress symptoms (CB-PTSS) and 4.7% of mothers meet the diagnostic criteria for childbirth-related post-traumatic stress disorder (CB-PTSD) [1]. PTSD is a psychiatric disorder, highly comorbid with anxiety and depression [2], with distressing symptoms including intrusions, avoidance of trauma-related cues, negative alterations in cognitions and mood, and alterations in arousal and reactivity [3]. Intrusive memories (IM), a core symptom of PTSD, are recurrent and involuntary recollections of the trauma memory [4]. IM are vivid, multisensory, predominantly visual, fragments of the trauma, and are highly distressing [5]. CB-PTSS negatively affect various facets of family life, such as the mother-infant relationship, breastfeeding, and child development [6]. Despite their high prevalence, there is currently only limited evidence concerning the effectiveness of interventions to reduce CB-PTSS [7]. Cognitive-behavioural therapy is currently one of the most common evidence-based interventions for PTSD, but 40 to 50% of patients are unresponsive to it [8]. Furthermore, access to such interventions is limited due to costs arising from numerous sessions with highly trained clinicians and lack of such therapists. Thus, it is essential to develop brief, easily accessible, and resource-efficient psychological interventions adapted to the perinatal context.

For such interventions, IM are targets of particular interest since they involve re-experiencing distressing moments of the trauma and may trigger or maintain other PTSS [9]. For example, high frequency of IM is associated with more difficulties in concentration [10]. New interventions targeting IM could therefore reduce not only intrusions but also other (CB-)PTSS, such as avoidance symptoms [11], and may thus be an innovative solution to support traumatised individuals.

Interventions assumed to tackle intrusive memories through interference with memory reconsolidation

Each new event initiates memory consolidation, a time-dependent process during which memories are malleable until their stabilisation into long-term memory within hours [12,13]. Acute emotional experiences (e.g., traumatic events) trigger a physiological stress response that modulates memory consolidation, resulting in particularly strong and lasting memories [14]. Furthermore, in healthy volunteers, physiological stress reactivity during a traumatic film appears to increase the number of IM in the following days [15]. This suggests that emotional arousal linked to traumatic events impacts memory formation, by enhancing the consolidation of distressing memories [14], and finally increase IM.

For peer review only

Although emerging evidence supports the efficacy of interventions assumed to interfere with trauma memory consolidation to prevent the development of PTSS [16], it is not always possible to intervene within the hours following a traumatic event [17]. A growing body of literature supports the assumption that memories are not fixed forever after consolidation, but that they can become malleable again when reactivated, regardless of the time passed since the traumatic event [18,19]. Reactivating a memory makes it malleable again for a few hours, until its re-stabilisation into long-term memory, corresponding to the reconsolidation process [20]. Consequently, memory reconsolidation is hypothesised to be a new window of opportunity to tackle IM [21] and reduce PTSS.

Interventions assumed to interfere with memory (re)consolidation have used various interference strategies, such as pharmacological agents, psychological therapy techniques, or behavioural interventions involving a visuospatial task (BI-VT) [16]. Such visuospatial tasks (e.g., Tetris gameplay), applied after assumes memory reactivation, possibly interfere with the reconsolidation of traumatic images by taxing the same resources needed by the visuospatial working memory to re-stabilise them [21,22]. These tasks appear as the most appropriate in the perinatal context (e.g., no drug contraindications, accessible to any trained clinicians, and minimum exposure to the trauma memory). So far, lab studies support indeed found that a visuospatial task delivered after the reactivation of a traumatic film memory leads to a reduction of the number of IM in healthy volunteers [23,24]. Further, a few translational studies reported encouraging results regarding the efficacy of BI-VT on the number of IM and PTSS in clinical samples [25,26]. For example, one BI-VT was applied to participants suffering from complex PTSD in an open-label single case series (*n*=20) [25]. After repeated sessions, during which participants produced writing narratives of a self-selected IM and then played Tetris for 25 minutes, a mean reduction of 64% of the targeted IM frequency was observed, whereof 16 out of the 20 participants reported an IM frequency reduction of at least 50%. In another study, repeated sessions of a BI-VT including Tetris gameplay also resulted in a decrease of the number of IM of refugees in a small single case series (*n*=4) [26].

Developing an evidence-based single-session BI-VT adapted to the perinatal context

A single-session BI-VT was recently adapted to the perinatal context in a pre-post pilot study (*n*=18) [27]. During the intervention, participants briefly narrated their childbirth on the maternity ward where they had given birth, as evidence suggest that being in a similar physical context as the one in which the traumatic event occurred improves the chances of successful memory reactivation [28]. Narration of their childbirth, ensuring a reliable source of the original memory [29], lasted 10 minutes for a sufficient exposure to the reminder cues supposed to engage memory reconsolidation of strong and old memories [30], such as trauma-related memories. Participants then played Tetris during 20 minutes in a neutral room. This single-session BI-VT led to a median reduction of 82% of the number of childbirth-related IM (CB-IM) [27]. Participants also reported an average reduction of 57% of CB-PTSS severity at one-

For peer review only

month post-intervention and rated the intervention as highly acceptable. Characteristics (distress, nowness, and sensorial modalities) of CB-IM were also described in this study, but a larger sample size is needed to better understand the nature of CB-IM and their responsiveness to such intervention.

These preliminary results suggest that a single-session BI-VT is suitable for the perinatal context. Building on this pilot study, a RCT will then allow to control for other potential confounding factors, such as the time between the trauma and the intervention [31]. Since, to our knowledge, no equivalent single-session intervention aimed at reducing CB-IM exists, a comparative treatment control group was not applicable. Therefore, to test the efficacy of a BI-VT to reduce CB-PTSS and to allow all help-seeking participants to benefit from the likely positive effects of this BI-VT, this RCT will include a waitlist control group as a comparator. As waitlists can create a nocebo condition, which might bias participants' self-reported symptoms [32] and artificially increase the estimated effects of the experimental intervention [33], participants will be blind to group allocation in an effort to limit these biases. Even though this RCT including a waitlist control group will not allow to study the specific effect of the mechanisms of this BI-VT on CB-PTSS in comparison to other interventions, it will bring more evidence to define this BI-VT as potentially *efficacious* to reduce CB-PTSS [31]. Given the lack of evidence supporting current interventions to treat established CB-PTSS [34], developing a single-session, low cost, easily accessible, and evidence-based intervention would be of great clinical value and might reduce long-term difficulties of mothers after traumatic childbirth.

One challenge for the translational research on memory reconsolidation is to identify if each supposed memory process is taking place and at which point [35]. No markers of these memory processes exist so far, but emotional arousal may be used to infer if participants have been sufficiently engaged in the narration of their traumatic memory to trigger memory reactivation. Previous findings revealed that a decrease in emotional arousal after playing Tetris is associated to an increased responsiveness to a similar BI-VT [25]. Therefore, we will include emotional and physiological measures of arousal (e.g., subjective distress and heart rate variability (HRV) [36,37]) to explore conditions enabling a successful implementation of a BI-VT in the perinatal context. In the future, such an assessment of physiological/emotional arousal could guide a personalised adaptation of this type of intervention [38].

Aims of the present study

This study aims to test the efficacy of a single-session BI-VT in reducing the number of CB-IM and CB-PTSS, in comparison to a waitlist. This intervention is assumed to reactivate the childbirth memory and interfere with its reconsolidation, respectively through a brief childbirth narration and Tetris gameplay, leading to a decrease in CB-IM and CB-PTSS. The primary outcome will be the difference in the number of CB-IM between baseline (two weeks pre-immediate intervention) and the two weeks post-immediate intervention in the Immediate Intervention Group (IIG) compared to the Waitlist Control Group (WCG)

For peer review only

(see Figure 1. for the study flow). Secondary outcomes will include group differences in CB-PTSS between baseline and at two weeks post-immediate intervention, as well as the acceptability of the intervention assessed in both groups. The following secondary outcomes will concern the IIG: the change in CB-PTSS between baseline and follow-up (at six week post-immediate intervention), and the change in the number of CB-IM between baseline and follow-up (weeks five and six post-immediate intervention). In addition, this study will explore, in both groups, the course of the nature (distress, nowness, and sensorial modality) and content of CB-IM before and after the intervention, as well as associations between participants' HRV and subjective distress course during the intervention, and the intervention efficacy. In the IIG, we will additionally explore the course of postnatal depression and anxiety symptoms at six weeks post-immediate intervention. Finally, this study will explore the change in the number CB-IM and CB-PTSS between the two weeks pre- and post-delayed intervention in the WCG.

METHODS AND ANALYSIS

Study design

The present study will be a single-blind waitlist RCT. The trial will include two arms: the IIG receiving the immediate intervention on day 15, and the WCG receiving the same intervention on day 30 (delayed intervention).

Study population, recruitment, group allocation, and blinding

Mothers with a minimum age of 18 years, who gave birth at least six weeks prior to the screening in one of the study centres (one university hospital being the main study centre and one regional hospital), and suffer from CB-IM will be eligible to participate. To avoid a floor effect regarding CB-IM reduction, we will only include mothers reporting at least four CB-IM over the two weeks prior to the screening. Exclusion criteria will be: insufficient French-speaking level to complete the assessments, established intellectual disability or psychotic illness, an ongoing psychological treatment in relation to childbirth, propranolol medication (due to suspected effects on memory reconsolidation [39]), alcohol and/or illicit drug abuse, being pregnant, perinatal loss of a child born during the index traumatic childbirth (i.e. the childbirth triggering the CB-IM targeted during the intervention), or a life-threatening illness of the mother or infant(s). Moreover, mothers not able to distinguish potential IM related to another traumatic event from CB-IM linked to the index traumatic childbirth will be excluded. Importantly, data related to any participants that reported less than two CB-IM at baseline will be excluded from relevant analyses to avoid a floor effect, or if they stated having a concurrent psychological treatment in relation to childbirth during study participation.

For peer review only - http://bmjopen.bmj.com/site/about/guidelines.xhtml

Adverts will be displayed in nurseries and practices of midwives, gynaecologists, and paediatricians. Study information will also be shared on social media. In addition, study centres will send a text message to invite potentially eligible mothers who gave birth in their hospitals to participate in the study.

During a phone call, the study coordinator (DF) will screen interested mothers. The presence and number of CB-IM over the past two weeks will be assessed with the item B1 of the Clinician-administered PTSD scale for Diagnostic and Statistical Manual of Mental Disorders, Fifth Edition, (DSM-5) [40], and the T-ACE will be used to assess alcohol consumption [41]. Eligible mothers who signed the consent form will be randomly allocated to the IIG or WCG with a 1:1 ratio. To ensure concealment, a scientific collaborator not involved in data collection created a block randomisation list stratified by centre, which will be automatically generated by the Research Electronic Data Capture (REDCap) software. To limit waitlist condition-related bias on participants' self-reports [32,33], participants will be blind to group allocation. To that matter, participants will only be informed that both groups will complete the same assessments and receive the intervention, but in a different order. For an overview of study procedures for both groups, see Figure 1.

Study procedure

T1-Baseline

All participants will receive questionnaires regarding sociodemographic and obstetric information, postnatal depression and anxiety symptoms on the first day of the baseline. They will report their CB-IM in diaries during the two weeks pre-immediate intervention. They will also report their CB-PTSS in a self-report questionnaire during week 2.

T2-Immediate intervention

The IIG will receive the intervention on day 15. For a description of intervention procedures, see the intervention section.

T3-Post-immediate intervention

Both groups will report their CB-IM during the two weeks post-immediate intervention. To ensure blinding of WCG, this group will complete a diary covering T1 and T3. Both groups will report their CB-PTSS in a self-report questionnaire during week 2 post-immediate intervention.

T4-Delayed intervention

The WCG will receive the delayed intervention on day 30. The delayed intervention follows the exact same procedures as the immediate intervention.

For peer review only

T5-Post-delayed intervention

The WCG will report their CB-IM in a diary during the two weeks post-delayed intervention and their CB-PTSS in a self-report questionnaire during week 2 post-delayed intervention.

T6-Follow-up

The IIG will report their CB-IM in a diary during weeks five and six post-immediate intervention. They will also complete self-report questionnaire assessing postnatal depression and anxiety symptoms, as well as CB-PTSS during week 6 post-immediate intervention. The WCG will not complete follow-up assessments to ensure a fair distribution of burden between groups

T7-Feedback phone call

Both groups will complete a feedback questionnaire by phone.

Intervention

The study coordinator, a trained psychologist, will meet the participant in a neutral room, inside a non-medical administrative building, explain intervention procedures, and attach an electrocardiogram (ECG) device to their chest. To assess HRV baseline, the participant will count aloud during two minutes (Count aloud task A). The study coordinator and the participant will then go into a gynaecological examination room of the maternity ward to realise the first part of the intervention:

1. Childbirth narrative task (assumed memory reactivation phase): The participant will be asked to briefly narrate their childbirth orally during 10 minutes. The study coordinator will first ask participants to narrate the overall course of their childbirth during five to seven minutes (Narrative task A). Then, the participant will be asked to narrate the moment associated to the most frequent or distressing CB-IM reported in the diary, for another three to five minutes (Narrative task B). If the participant perceived her distress to be overwhelming, she will have the option to go into another room not located in the maternity or to write about her childbirth instead of narrating it orally.

The participant and the study coordinator will then return to the neutral room to start the second part of the intervention, at least ten minutes later, in order to allow the assumed memory labilisation [42] and to avoid further memory reactivation during Tetris gameplay:

2. Tetris gameplay (assumed memory reconsolidation disruption phase): The study coordinator will ask the participant to play Tetris on a handheld gaming device (Nintendo 3DS), in "Marathon" mode, with both sound and 3D switched off. The participant will be

For peer review only

instructed to focus both on the block currently falling and on the upcoming blocks (shown in a preview box). To promote mental rotation, participants will be encouraged to use their imagination to visualise how to place each block to complete as many horizontal lines as possible [24]. The participant will have a three-minute practice trial before playing Tetris for 20 minutes uninterrupted. The study coordinator will stay in the room to ensure that the participant will stay focused on Tetris during the 20 minutes-long gameplay (e.g., instead of looking at her phone).

After Tetris gameplay, the participant will count aloud for two minutes to measure HRV recovery (Count aloud task B). Finally, the participant will be asked not to play Tetris or to research the therapeutic use of Tetris until she terminated the study.

An overview of the intervention procedures is shown in Figure 2.

Primary outcome

The primary outcome will be the difference in the number of CB-IM between baseline and the two weeks post-immediate intervention across groups.

Secondary outcomes

Secondary outcomes include:

- The change in CB-PTSS between: a) baseline and at two weeks post-immediate intervention across groups, b) baseline and follow-up in the IIG.
- The change in the number of CB-IM between baseline and follow-up in the IIG.
- The acceptability of the intervention in both groups

Exploratory outcomes

This study will investigate the following exploratory outcomes in both groups:

- The course of the distress, nowness, sensorial modalities, and content related to CB-IM before and after the intervention.
- The association of HRV and subjective distress assessed during the different phases of the intervention, with the intervention efficacy (number of CB-IM and CB-PTSS post-intervention).

In addition, exploratory outcomes will include in the IIG:

- The change in postnatal depression, and anxiety symptoms between baseline and follow-up.

For peer review only

- The change in the number of CB-IM between the two weeks post-immediate intervention and follow-up.
- The change in CB-PTSS between two weeks post-immediate intervention and follow-up.

We will also explore, in the WCG, the change in the number of CB-IM and CB-PTSS between the two weeks pre- and post-delayed intervention.

Data collection

Table 1 provides an overview of study outcomes and related time points.

For peer review only

Table 1. Overview of study outcomes and time points.

Variables	Instruments	Study period						
		**T1** Baseline	**T2** Immediate intervention	**T3** Post-immediate intervention	**T4** Delayed intervention	**T5** Post-delayed intervention	**T6** Follow-up	**T7** Feedback phone call
CB-IM	Diaries	IIG + WCG		IIG + WCG		WCG	IIG	
Content of CB-IM		IIG + WCG		IIG + WCG		WCG	IIG	
Distress of CB-IM		IIG + WCG		IIG + WCG		WCG	IIG	
Nowness of CB-IM		IIG + WCG		IIG + WCG		WCG	IIG	
Sensorial modalities of CB-IM		IIG + WCG		IIG + WCG		WCG	IIG	
CB-PTSS	City Birth Trauma Scale [43]	IIG + WCG		IIG + WCG		WCG	IIG	
Anxiety	Hospital Anxiety and Depression scale – Anxiety subscale [44]	IIG + WCG					IIG	
Postnatal depression	Edinburgh Postnatal Depression Scale [45]	IIG + WCG					IIG	
Heart rate variability	Firstbeat Bodyguard 2 ECG device		IIG		WCG			
Subjective distress	Visual Analogue Scales		IIG		WCG			
Intervention acceptability	Feedback questionnaire III							IIG + WCG
Sociodemographic variables	Sociodemographic questionnaire	IIG + WCG						
Obstetric and medical data	Medical records & self-report questionnaire	IIG + WCG						

Note: IIG, immediate intervention group; WCG, waitlist control group; CB-IM, childbirth-related intrusive memories; CB-PTSS, Childbirth-related posttraumatic stress symptoms; ECG, Electrocardiogram; T1; Weeks 1 & 2 pre-immediate intervention; T2, Day 15; T3, Weeks 1 & 2 post-immediate intervention; T4, Day 30; T5, Weeks 1 & 2 post-delayed intervention; T6, Weeks 5 & 6 post-immediate intervention.

For peer review only

Measures

1. Childbirth-related posttraumatic stress symptoms

1.1. Diaries

For each CB-IM, participants will be asked to write in a diary the time it occurred, its content, and to report, from 0=*not at all* to 10=*extremely*, 1) how distressing it was, 2) to what extend they had the impression that the memory was happening here and now (*nowness* [46]), and 3) its sensorial modalities. CB-IM will be defined to participants as "*involuntary memories of the labour or childbirth that come to mind without warning. They can be vivid and emotional, and are often like a mental image, such as a photograph or film. They can involve all the senses (…). They can be very short, elusive and fragmented.*"[27]. Participants will use electronic diaries hosted in REDCap, or a paper version (e.g., if not able to use the electronic ones), adapted from previous work [27].

1.2. City Birth Trauma Scale (CBTS)

This questionnaire assesses CB-PTSS during the past week, and diagnostic criteria of PTSD according to the DSM-5 [43]. Twenty-two items, using a 4-point Likert scale, assess the four CB-PTSS clusters (*re-experiencing*, *avoidance*, *negative cognitions and mood*, *hyperarousal*). Original instructions of the CBTS will be adapted for participants who had multiple childbirth(s) to complete the questionnaire regarding the index traumatic childbirth. Good psychometric properties were found for the French version of the CBTS [47].

2. Anxiety and depression symptoms

2.1. Anxiety subscale of the Hospital Anxiety and Depression Scale (HADS-A)

This subscale assess anxiety symptoms and consists of seven items using 4-point Likert scales [44]. Good psychometric properties are reported for the French version of the HADS [48].

2.2. Edinburgh Postnatal Depression Scale (EPDS)

This 10-item questionnaire measures postnatal depression symptoms during the past week with 4-point Likert scales [45]. The French version of the EPDS has good psychometric properties [49].

3. Intervention-related measures

3.1. Intervention acceptability

For peer review only

Participants will answer the Feedback Questionnaire III assessing intervention acceptability, usefulness, and expectations regarding its effects. These questions, based on previous work [27], have been designed to best match the intervention characteristics.

3.2. Heart rate variability

Participants' HRV will be recorded using a Firstbeat Bodyguard 2 (Firstbeat Technologies Ltd., Jyväskylä, Finland) ECG device during the whole intervention procedures. Count aloud tasks A & B will measure HRV baseline and recovery, in order to compare participants HRV during Narrative tasks A & B, and Tetris gameplay.

3.3. Subjective distress

Visual analogue scales will be presented to participants at 11 time points during the intervention to assess their subjective distress level from 0=*not stressed and/or anxious at all* to 10=*extremely stressed and/or anxious*.

4. Sociodemographic and obstetric characteristics

*4.1. Sociodemographic variable*s

A sociodemographic questionnaire will assess participants' age, nationality, marital status, and education level.

4.2. Obstetric and medical data

Obstetric and medical data will be extracted from participants' medical record, such as childbirth mode, gestational age, and Apgar scores. Birth-related information will also be collected at baseline, through a self-report questionnaire (e.g., presence of a relative, perceived threat for self or infant).

5. Other variables

This study includes other outcomes, aiming to specify the effects of the intervention, presented in Table 2.

For peer review only

Table 2. Other outcomes.

Variables	Instruments	Time points	Description
Cognitive flexibility	Cognitive flexibility scale [50]	T1	Cognitive flexibility defines, inter alia, the capacity to adapt to the situation and being flexible [50].
Cue specificity	Feedback questionnaires I & III	T2/T4, T7	These questionnaires will assess the similarity between childbirth narration and the original childbirth memory.
Context specificity	Feedback questionnaires II & III	T3/T5, T7	Questions will assess the similarity between the intervention context and the original traumatic context.
Diary compliance	Diaries	T1, T3, T5, T6	Diary compliance will check the extent to which participants were able/remembered to report their CB-IM at the end of each diary.
HRV control variables	Questionnaire on HRV control variables	T1, T2/T4	This questionnaire will contain control variables regarding the analyses of the HRV (e.g., weight, height, medicine use, cigarettes and alcohol consumption).
Video games use	Feedback questionnaires I & III	T2/T4, T7	Questions will assess participants' video games use (e.g., engagement in the game during the intervention, Tetris perceived difficulty, video games habits, and Tetris use post-intervention).
Sleep	Sleep questionnaire	T3/T5	This questionnaire will evaluate the number of hours of sleep during the 24 hours following the intervention.
Concurrent psychological treatment	Feedback questionnaire III	T7	A question will check the absence of a concurrent psychological treatment in relation to the index childbirth during study participation.

Note: CB-IM, childbirth-related intrusive memories; HRV, heart rate variability. T1; Weeks 1 & 2 pre-immediate intervention; T2, Day 15; T3, Weeks 1 & 2 post-immediate intervention; T4, Day 30; T5, Weeks 1 & 2 post-delayed intervention; T6, Weeks 5 & 6 post-immediate intervention; T7, Feedback phone call.

Sample size calculation

A sample size calculation was conducted for our primary and secondary outcomes based on the results obtained from the pilot study [27], assessing the efficacy of the same intervention using a single-group pre-post design, and from laboratory studies using RCT designs to assess the efficacy of a similar BI-VT [23,24]. A sample size of 90 participants (45 allocated in each group) is estimated to be necessary to detect the effect of the intervention on our primary and secondary outcomes with an 80% power (α = 0.05). Based on our experience with this study population [11,27], a conservative estimate of a 30% drop-out and exclusion was assumed. We therefore plan to recruit 120 participants in total.

Data management and statistical analysis

Participants' entries in electronic diaries and questionnaires will be directly stored in the REDCap software. The research staff will enter manually the rest of the data collected during the screening, the

For peer review only

intervention, and paper diaries in REDCap. All data will be hosted on a high security server of the Lausanne University Hospital performing automatic continuous backups. All primary outcome data and a random 5% of the rest of the data will be double-checked. Independent and blind coders will inspect diary entries to detect any non-compliance with instructions and count the number of CB-IM. A blind statistician will perform the analyses. Only the study team will have access to the full coded trial dataset.

For primary outcomes, group differences regarding change of the number of CB-IM between baseline and the two weeks post-immediate intervention will be analysed using repeated-measures analyses (intention-to-treat analyses). For the secondary outcomes, repeated-measures analyses will be performed to analyse group differences concerning CB-PTSS between baseline and at two weeks post-immediate intervention. Logistic regression analyses will also compare the proportion of participants meeting the diagnostic criteria for CB-PTSD across time and groups. Furthermore, repeated measures analyses will serve to analyse change in the number of CB-IM and CB-PTSS between baseline and follow-up in the IIG. Group differences regarding the proportion of participants meeting the diagnostic criteria for CB-PTSD will be analysed with logistic regression analyses. The mean score of the intervention acceptability will be calculated across both groups. Additional post-hoc analyses conducted will be described as such in publications. Missing data will be dealt with using multiple imputation procedures, if appropriate.

ETHICS AND DISSEMINATION

Ethical approval of this protocol was granted by the Human Research Ethics Committee of the Canton de Vaud (study number 2022-00652). The study will be monitored by an independent and certified data monitoring body. A scientific steering committee is consulted regularly for scientific decisions regarding the conduct of the study. This protocol was registered in ClinicalTrials.gov prior to data collection (NCT05381155). Results of this study will be submitted for publication to peer-reviewed journals and presented in relevant conferences to ensure dissemination in the scientific community. Furthermore, data will be shared in an anonymised form, at the time of the publication of the related outcomes, in an open access register.

PATIENT AND PUBLIC INVOLVEMENT

Participants will evaluate the intervention acceptability and will be able to give feedback on the intervention. Each participant will receive a personalised debriefing session. Study results will be sent to participants and shared with the public via social media and public events.

SAFETY

Any serious adverse event occurring during data collection will be documented and reported to the competent ethics committee.

For peer review only

AUTHORS' CONTRIBUTION

This protocol was designed by DF, CD and AH. DF, CD and AH drafted the current manuscript. Each author (DF, CD, NMB, TM, DB, JL, UR and AH) contributed to the revision and implementation of study procedures. All authors critically revised the manuscript and approved the final version of the manuscript.

FUNDING STATEMENT

This study is supported by a project grant from the Swiss National Science Foundation (SNF IZCOZ0_205434/1).

COMPETING INTERESTS STATEMENT

None declared.

REFERENCES

1. Heyne CS, Kazmierczak M, Souday R, et al. Prevalence and risk factors of birth-related posttraumatic stress among parents: A comparative systematic review and meta-analysis. *Clin Psychol Rev* 2022;94:102157. doi: 10.1016/j.cpr.2022.102157 [published Online First: 2022/05/19]
2. Morland L, Goebert D, Onoye J, et al. Posttraumatic stress disorder and pregnancy health: preliminary update and implications. *Psychosomatics* 2007;48(4):304-8. doi: 10.1176/appi.psy.48.4.304 [published Online First: 2007/06/30]
3. American Psychiatric Association. Diagnostic and statistical manual of mental disorders (5th ed.). Washington, DC: American Psychiatric Publishing 2013.
4. Ehlers A, Clark DM. A cognitive model of posttraumatic stress disorder. *Behav Res Ther* 2000;38(4):319-45. doi: 10.1016/s0005-7967(99)00123-0
5. Ehlers A, Hackmann A, Michael T. Intrusive re-experiencing in post-traumatic stress disorder: phenomenology, theory, and therapy. *Memory* 2004;12(4):403-15. doi: 10.1080/09658210444000025 [published Online First: 2004/10/19]
6. Cook N, Ayers S, Horsch A. Maternal posttraumatic stress disorder during the perinatal period and child outcomes: A systematic review. *J Affect Disord* 2018;225:18-31. doi: 10.1016/j.jad.2017.07.045 [published Online First: 2017/08/05]
7. Furuta M, Horsch A, Ng ESW, et al. Effectiveness of Trauma-Focused Psychological Therapies for Treating Post-traumatic Stress Disorder Symptoms in Women Following Childbirth: A Systematic Review and Meta-Analysis. *Front Psychiatry* 2018;9:591-91. doi: 10.3389/fpsyt.2018.00591
8. Loerinc AG, Meuret AE, Twohig MP, et al. Response rates for CBT for anxiety disorders: Need for standardized criteria. *Clin Psychol Rev* 2015;42:72-82. doi: 10.1016/j.cpr.2015.08.004 [published Online First: 2015/09/01]
9. Iyadurai L, Visser RM, Lau-Zhu A, et al. Intrusive memories of trauma: A target for research bridging cognitive science and its clinical application. *Clin Psychol Rev* 2019;69:67-82. doi: 10.1016/j.cpr.2018.08.005 [published Online First: 2018/10/09]
10. Holmes EA, Ghaderi A, Eriksson E, et al. 'I Can't Concentrate': A Feasibility Study with Young Refugees in Sweden on Developing Science-Driven Interventions for Intrusive Memories Related to Trauma. *Behav Cogn Psychother* 2017;45(2):97-109. doi: 10.1017/s135246581600062x [published Online First: 2017/02/24]

For peer review only

11. Horsch A, Vial Y, Favrod C, et al. Reducing intrusive traumatic memories after emergency caesarean section: A proof-of-principle randomized controlled study. *Behav Res Ther* 2017;94:36-47. doi: 10.1016/j.brat.2017.03.018 [published Online First: 2017/04/30]
12. Nadel L, Hupbach A, Gomez R, et al. Memory formation, consolidation and transformation. *Neurosci Biobehav Rev* 2012;36(7):1640-5. doi: 10.1016/j.neubiorev.2012.03.001 [published Online First: 2012/04/03]
13. McGaugh JL. Memory--a century of consolidation. *Science* 2000;287(5451):248-51. doi: 10.1126/science.287.5451.248 [published Online First: 2000/01/15]
14. McGaugh JL. The amygdala modulates the consolidation of memories of emotionally arousing experiences. *Annu Rev Neurosci* 2004;27:1-28. doi: 10.1146/annurev.neuro.27.070203.144157 [published Online First: 2004/06/26]
15. Schultebraucks K, Rombold-Bruehl F, Wingenfeld K, et al. Heightened biological stress response during exposure to a trauma film predicts an increase in intrusive memories. *J Abnorm Psychol* 2019;128(7):645-57. doi: 10.1037/abn0000440 [published Online First: 2019/07/10]
16. Astill Wright L, Horstmann L, Holmes EA, et al. Consolidation/reconsolidation therapies for the prevention and treatment of PTSD and re-experiencing: a systematic review and meta-analysis. *Transl Psychiatry* 2021;11(1):453. doi: 10.1038/s41398-021-01570-w [published Online First: 2021/09/05]
17. Phelps EA, Hofmann SG. Memory editing from science fiction to clinical practice. *Nature* 2019;572(7767):43-50. doi: 10.1038/s41586-019-1433-7 [published Online First: 2019/08/02]
18. Agren T. Human reconsolidation: a reactivation and update. *Brain Res Bull* 2014;105:70-82. doi: 10.1016/j.brainresbull.2013.12.010 [published Online First: 2014/01/09]
19. Misanin JR, Miller RR, Lewis DJ. Retrograde amnesia produced by electroconvulsive shock after reactivation of a consolidated memory trace. *Science* 1968;160(3827):554-5. doi: 10.1126/science.160.3827.554 [published Online First: 1968/05/03]
20. Lee JLC, Nader K, Schiller D. An Update on Memory Reconsolidation Updating. *Trends Cogn Sci* 2017;21(7):531-45. doi: 10.1016/j.tics.2017.04.006 [published Online First: 2017/05/13]
21. Visser RM, Lau-Zhu A, Henson RN, et al. Multiple memory systems, multiple time points: how science can inform treatment to control the expression of unwanted emotional memories. *Philos Trans R Soc Lond B Biol Sci* 2018;373(1742) doi: 10.1098/rstb.2017.0209 [published Online First: 2018/01/21]
22. Holmes EA, James EL, Coode-Bate T, et al. Can playing the computer game "Tetris" reduce the build-up of flashbacks for trauma? A proposal from cognitive science. *PLoS One* 2009;4(1):e4153. doi: 10.1371/journal.pone.0004153 [published Online First: 2009/01/08]
23. Kessler H, Schmidt AC, James EL, et al. Visuospatial computer game play after memory reminder delivered three days after a traumatic film reduces the number of intrusive memories of the experimental trauma. *J Behav Ther Exp Psychiatry* 2020;67:101454. doi: 10.1016/j.jbtep.2019.01.006 [published Online First: 2019/05/01]
24. James EL, Bonsall MB, Hoppitt L, et al. Computer Game Play Reduces Intrusive Memories of Experimental Trauma via Reconsolidation-Update Mechanisms. *Psychol Sci* 2015;26(8):1201-15. doi: 10.1177/0956797615583071 [published Online First: 2015/07/03]
25. Kessler H, Holmes EA, Blackwell SE, et al. Reducing intrusive memories of trauma using a visuospatial interference intervention with inpatients with posttraumatic stress disorder (PTSD). *J Consult Clin Psychol* 2018;86(12):1076-90. doi: 10.1037/ccp0000340 [published Online First: 2018/12/07]
26. Kanstrup M, Kontio E, Geranmayeh A, et al. A single case series using visuospatial task interference to reduce the number of visual intrusive memories of trauma with refugees. *Clin Psychol Psychother* 2021;28(1):109-23. doi: 10.1002/cpp.2489 [published Online First: 2020/06/12]
27. Deforges C, Fort D, Stuijfzand S, et al. Reducing childbirth-related intrusive memories and PTSD symptoms via a single-session behavioural intervention including a visuospatial task: A proof-of-principle study. *J Affect Disord* 2022;303:64-73. doi: 10.1016/j.jad.2022.01.108 [published Online First: 2022/02/03]
28. Hupbach A, Hardt O, Gomez R, et al. The dynamics of memory: context-dependent updating. *Learn Mem* 2008;15(8):574-9. doi: 10.1101/lm.1022308 [published Online First: 2008/08/08]

For peer review only - http://bmjopen.bmj.com/site/about/guidelines.xhtml

29. Debiec J, Diaz-Mataix L, Bush DE, et al. The selectivity of aversive memory reconsolidation and extinction processes depends on the initial encoding of the Pavlovian association. *Learn Mem* 2013;20(12):695-9. doi: 10.1101/lm.031609.113 [published Online First: 2013/11/21]
30. Suzuki A, Josselyn SA, Frankland PW, et al. Memory reconsolidation and extinction have distinct temporal and biochemical signatures. *J Neurosci* 2004;24(20):4787-95. doi: 10.1523/JNEUROSCI.5491-03.2004 [published Online First: 2004/05/21]
31. Chambless DL, Hollon SD. Defining empirically supported therapies. *J Consult Clin Psychol* 1998;66(1):7-18. doi: 10.1037//0022-006x.66.1.7 [published Online First: 1998/03/07]
32. Furukawa TA, Noma H, Caldwell DM, et al. Waiting list may be a nocebo condition in psychotherapy trials: a contribution from network meta-analysis. *Acta Psychiatr Scand* 2014;130(3):181-92. doi: 10.1111/acps.12275 [published Online First: 2014/04/05]
33. Faltinsen E, Todorovac A, Staxen Bruun L, et al. Control interventions in randomised trials among people with mental health disorders. *Cochrane Database Syst Rev* 2022;4(4):MR000050. doi: 10.1002/14651858.MR000050.pub2 [published Online First: 2022/04/05]
34. de Bruijn L, Stramrood CA, Lambregtse-van den Berg MP, et al. Treatment of posttraumatic stress disorder following childbirth. *J Psychosom Obstet Gynaecol* 2020;41(1):5-14. doi: 10.1080/0167482X.2019.1593961 [published Online First: 2019/06/06]
35. Vaverkova Z, Milton AL, Merlo E. Retrieval-Dependent Mechanisms Affecting Emotional Memory Persistence: Reconsolidation, Extinction, and the Space in Between. *Front Behav Neurosci* 2020;14:574358. doi: 10.3389/fnbeh.2020.574358 [published Online First: 2020/11/03]
36. Halligan SL, Michael T, Wilhelm FH, et al. Reduced heart rate responding to trauma reliving in trauma survivors with PTSD: correlates and consequences. *J Trauma Stress* 2006;19(5):721-34. doi: 10.1002/jts.20167 [published Online First: 2006/11/01]
37. Haag K, Hiller R, Peyk P, et al. A Longitudinal Examination of Heart-Rate and Heart Rate Variability as Risk Markers for Child Posttraumatic Stress Symptoms in an Acute Injury Sample. *J Abnorm Child Psychol* 2019;47(11):1811-20. doi: 10.1007/s10802-019-00553-2 [published Online First: 2019/05/11]
38. Kindt M, Elsey JWB. A paradigm shift in the treatment of emotional memory disorders: Lessons from basic science. *Brain Res Bull* 2022;192:168-74. doi: 10.1016/j.brainresbull.2022.11.019 [published Online First: 2022/11/29]
39. Giustino TF, Fitzgerald PJ, Maren S. Revisiting propranolol and PTSD: Memory erasure or extinction enhancement? *Neurobiol Learn Mem* 2016;130:26-33. doi: 10.1016/j.nlm.2016.01.009 [published Online First: 2016/01/26]
40. Blake DD, Weathers FW, Nagy LM, et al. The development of a Clinician-Administered PTSD Scale. *J Trauma Stress* 1995;8(1):75-90. doi: 10.1007/BF02105408 [published Online First: 1995/01/01]
41. Sokol RJ, Martier SS, Ager JW. The T-ACE questions: practical prenatal detection of risk-drinking. *Am J Obstet Gynecol* 1989;160(4):863-8; discussion 68-70. doi: 10.1016/0002-9378(89)90302-5 [published Online First: 1989/04/01]
42. Agren T, Engman J, Frick A, et al. Disruption of reconsolidation erases a fear memory trace in the human amygdala. *Science* 2012;337(6101):1550-2. doi: 10.1126/science.1223006 [published Online First: 2012/09/22]
43. Ayers S, Wright DB, Thornton A. Development of a Measure of Postpartum PTSD: The City Birth Trauma Scale. *Front Psychiatry* 2018;9:409. doi: 10.3389/fpsyt.2018.00409 [published Online First: 2018/10/04]
44. Zigmond AS, Snaith RP. The hospital anxiety and depression scale. *Acta Psychiatr Scand* 1983;67(6):361-70. doi: 10.1111/j.1600-0447.1983.tb09716.x [published Online First: 1983/06/01]
45. Cox JL, Holden JM, Sagovsky R. Detection of postnatal depression. Development of the 10-item Edinburgh Postnatal Depression Scale. *Br J Psychiatry* 1987;150:782-6. doi: 10.1192/bjp.150.6.782 [published Online First: 1987/06/01]
46. Michael T, Ehlers A, Halligan SL, et al. Unwanted memories of assault: what intrusion characteristics are associated with PTSD? *Behav Res Ther* 2005;43(5):613-28. doi: 10.1016/j.brat.2004.04.006 [published Online First: 2005/05/04]

For peer review only

47. Sandoz V, Hingray C, Stuijfzand S, et al. Measurement and conceptualization of maternal PTSD following childbirth: Psychometric properties of the City Birth Trauma Scale-French Version (City BiTS-F). *Psychol Trauma* 2022;14(4):696-704. doi: 10.1037/tra0001068 [published Online First: 2021/07/23]
48. Untas A, Aguirrezabal M, Chauveau P, et al. [Anxiety and depression in hemodialysis: validation of the Hospital Anxiety and Depression Scale (HADS)]. *Nephrol Ther* 2009;5(3):193-200. doi: 10.1016/j.nephro.2009.01.007 [published Online First: 2009/04/07]
49. Guedeney N, Fermanian J. Validation study of the French version of the Edinburgh Postnatal Depression Scale (EPDS): new results about use and psychometric properties. *European Psychiatry* 2020;13(2):83-89. doi: 10.1016/s0924-9338(98)80023-0
50. Martin MM, Rubin RB. A New Measure of Cognitive Flexibility. *Psychological Reports* 2016;76(2):623-26. doi: 10.2466/pr0.1995.76.2.623

For peer review only

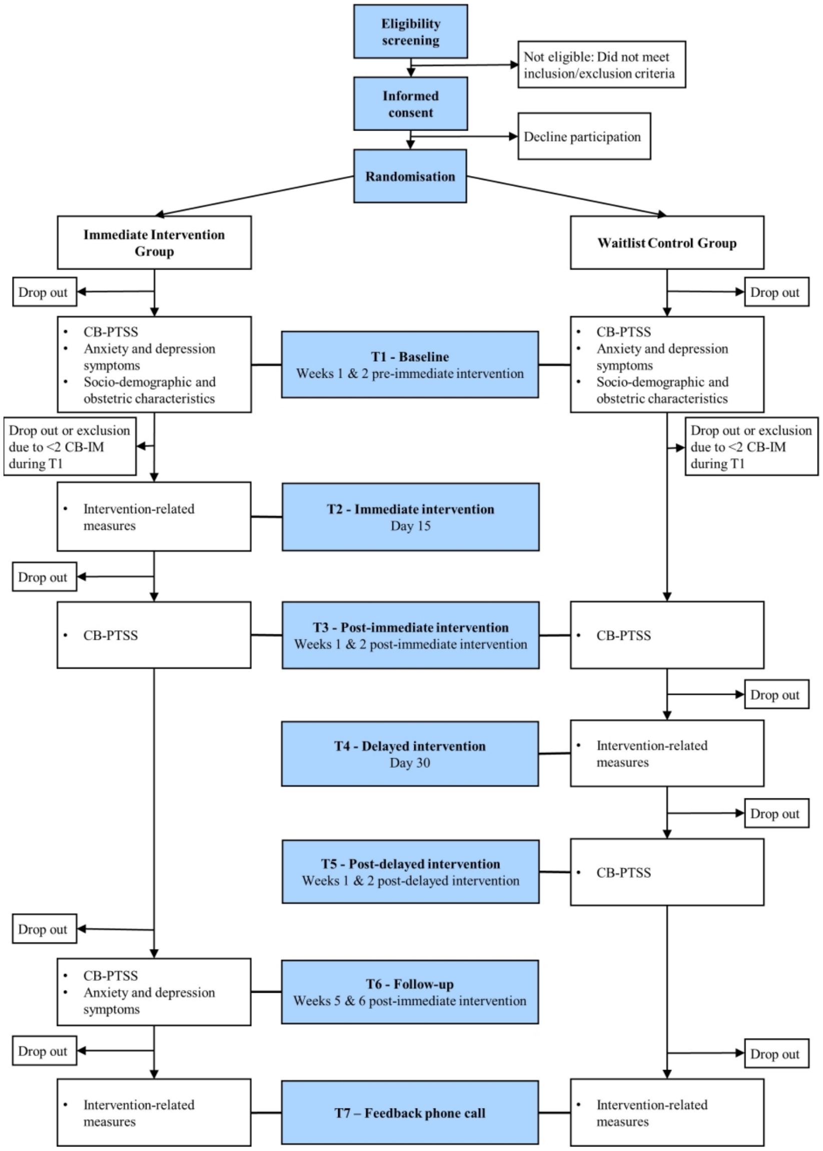

Figure 1. Flowchart of study procedures. Note: CB-PTSS, childbirth-related posttraumatic stress symptoms: CB-IM, childbirth-related intrusive memories.

190x275mm (300 x 300 DPI)

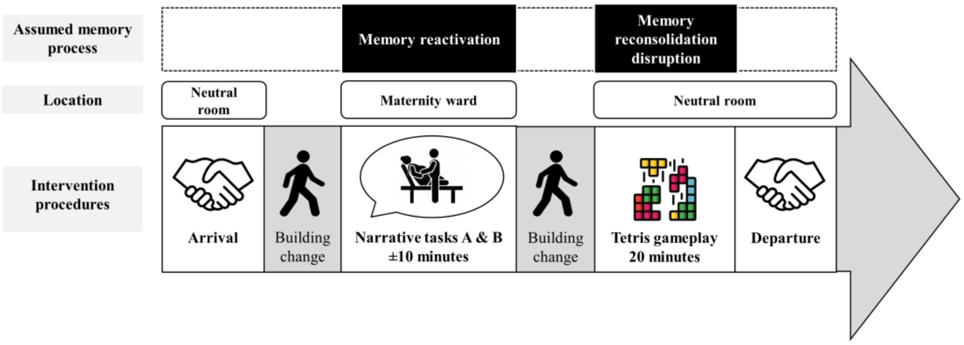

Figure 2. Intervention procedures. Adapted from Deforges et al. [27] with permission.

284x100mm (300 x 300 DPI)

SPIRIT
STANDARD PROTOCOL ITEMS: RECOMMENDATIONS FOR INTERVENTIONAL TRIALS

SPIRIT 2013 Checklist: Recommended items to address in a clinical trial protocol and related documents*

Section/item	Item No	Description	Addressed on page number
**Administrative information**			
Title	1	Descriptive title identifying the study design, population, interventions, and, if applicable, trial acronym	1
Trial registration	2a	Trial identifier and registry name. If not yet registered, name of intended registry	17
	2b	All items from the World Health Organization Trial Registration Data Set	17[1]
Protocol version	3	Date and version identifier	Issue Date: 1.12.2022<br>Protocol Amendment Number: 01
Funding	4	Sources and types of financial, material, and other support	18
Roles and responsibilities	5a	Names, affiliations, and roles of protocol contributors	1-2 & 18
	5b	Name and contact information for the trial sponsor	Bureau du Promoteur de Recherche<br>Avenue de Beaumont 21bis<br>1011 Lausanne<br>+41 21 314 39 05

[1] All information are accessible on ClinicalTrials.gov

For peer review only - http://bmjopen.bmj.com/site/about/guidelines.xhtml

	5c	Role of study sponsor and funders, if any, in study design; collection, management, analysis, and interpretation of data; writing of the report; and the decision to submit the report for publication, including whether they will have ultimate authority over any of these activities	Not applicable
	5d	Composition, roles, and responsibilities of the coordinating centre, steering committee, endpoint adjudication committee, data management team, and other individuals or groups overseeing the trial, if applicable (see Item 21a for data monitoring committee)	17
**Introduction**			
Background and rationale	6a	Description of research question and justification for undertaking the trial, including summary of relevant studies (published and unpublished) examining benefits and harms for each intervention	5-8
	6b	Explanation for choice of comparators	7
Objectives	7	Specific objectives or hypotheses	11-12
Trial design	8	Description of trial design including type of trial (eg, parallel group, crossover, factorial, single group), allocation ratio, and framework (eg, superiority, equivalence, noninferiority, exploratory)	8 & 9
**Methods: Participants, interventions, and outcomes**			
Study setting	9	Description of study settings (eg, community clinic, academic hospital) and list of countries where data will be collected. Reference to where list of study sites can be obtained	8[2]
Eligibility criteria	10	Inclusion and exclusion criteria for participants. If applicable, eligibility criteria for study centres and individuals who will perform the interventions (eg, surgeons, psychotherapists)	8-9 & 10

[2] The list of study sites can be obtained on ClinicalTrials.gov

For peer review only - http://bmjopen.bmj.com/site/about/guidelines.xhtml

Interventions	11a	Interventions for each group with sufficient detail to allow replication, including how and when they will be administered	9-11
	11b	Criteria for discontinuing or modifying allocated interventions for a given trial participant (eg, drug dose change in response to harms, participant request, or improving/worsening disease)	Not applicable
	11c	Strategies to improve adherence to intervention protocols, and any procedures for monitoring adherence (eg, drug tablet return, laboratory tests)	10-11 & 16[3]
	11d	Relevant concomitant care and interventions that are permitted or prohibited during the trial	8
Outcomes	12	Primary, secondary, and other outcomes, including the specific measurement variable (eg, systolic blood pressure), analysis metric (eg, change from baseline, final value, time to event), method of aggregation (eg, median, proportion), and time point for each outcome. Explanation of the clinical relevance of chosen efficacy and harm outcomes is strongly recommended	5, 11-12, & 14-15
Participant timeline	13	Time schedule of enrolment, interventions (including any run-ins and washouts), assessments, and visits for participants. A schematic diagram is highly recommended (see Figure)	9-10, 16 and Figure 1
Sample size	14	Estimated number of participants needed to achieve study objectives and how it was determined, including clinical and statistical assumptions supporting any sample size calculations	16
Recruitment	15	Strategies for achieving adequate participant enrolment to reach target sample size	9

Methods: Assignment of interventions (for controlled trials)

Allocation:

[3] Moreover, participants will receive reminders to complete questionnaires and the same person will follow participants' progression during the whole study.

For peer review only - http://bmjopen.bmj.com/site/about/guidelines.xhtml

Sequence generation	16a	Method of generating the allocation sequence (eg, computer-generated random numbers), and list of any factors for stratification. To reduce predictability of a random sequence, details of any planned restriction (eg, blocking) should be provided in a separate document that is unavailable to those who enrol participants or assign interventions	9
Allocation concealment mechanism	16b	Mechanism of implementing the allocation sequence (eg, central telephone; sequentially numbered, opaque, sealed envelopes), describing any steps to conceal the sequence until interventions are assigned	9
Implementation	16c	Who will generate the allocation sequence, who will enrol participants, and who will assign participants to interventions	9
Blinding (masking)	17a	Who will be blinded after assignment to interventions (eg, trial participants, care providers, outcome assessors, data analysts), and how	9 & 17[4]
	17b	If blinded, circumstances under which unblinding is permissible, and procedure for revealing a participant's allocated intervention during the trial	Not applicable

Methods: Data collection, management, and analysis

Data collection methods	18a	Plans for assessment and collection of outcome, baseline, and other trial data, including any related processes to promote data quality (eg, duplicate measurements, training of assessors) and a description of study instruments (eg, questionnaires, laboratory tests) along with their reliability and validity, if known. Reference to where data collection forms can be found, if not in the protocol	10, 14-15 & 16-17
	18b	Plans to promote participant retention and complete follow-up, including list of any outcome data to be collected for participants who discontinue or deviate from intervention protocols	The same person will follow participants's progression during the whole study. No outcome will be collected for participants who discontinue.

[4] To ensure blinding of the coders and the statisticians, they will receive a dataset not indicating which allocation group corresponds to the IIG or WCG.

For peer review only - http://bmjopen.bmj.com/site/about/guidelines.xhtml

Data management	19	Plans for data entry, coding, security, and storage, including any related processes to promote data quality (eg, double data entry; range checks for data values). Reference to where details of data management procedures can be found, if not in the protocol	16-17
Statistical methods	20a	Statistical methods for analysing primary and secondary outcomes. Reference to where other details of the statistical analysis plan can be found, if not in the protocol	16-17
	20b	Methods for any additional analyses (eg, subgroup and adjusted analyses)	16-17
	20c	Definition of analysis population relating to protocol non-adherence (eg, as randomised analysis), and any statistical methods to handle missing data (eg, multiple imputation)	16-17

Methods: Monitoring

For peer review only

Data monitoring	21a	Composition of data monitoring committee (DMC); summary of its role and reporting structure; statement of whether it is independent from the sponsor and competing interests; and reference to where further details about its charter can be found, if not in the protocol. Alternatively, an explanation of why a DMC is not needed	The monitoring will be performed by Fanny Muet of the Institut Universitaire de Formation et Recherche en Soins of the CHUV, in accordance with ICH GCP E6(R2). The monitor (independent of the research team) will carry out this activity according to a pre-established monitoring plan adapted to the risk of the clinical trial and according to the department's Standard Operating Procedures. The monitor will verify that the clinical trial is conducted and that the data are generated, documented and reported in accordance with the requirements of the protocol, the GCP and the applicable regulatory requirements. In concrete terms, an initiation visit, intermediate visits and a closing visit will be organized on site by the monitor in accordance with the monitoring plan.<br>The investigator-sponsor will ensure that the monitor has access to the trial data and source documents at all times and will respond to questions and requests for corrections during the monitoring visits.

	21b	Description of any interim analyses and stopping guidelines, including who will have access to these interim results and make the final decision to terminate the trial	Not applicable
Harms	22	Plans for collecting, assessing, reporting, and managing solicited and spontaneously reported adverse events and other unintended effects of trial interventions or trial conduct	17
Auditing	23	Frequency and procedures for auditing trial conduct, if any, and whether the process will be independent from investigators and the sponsor	An initiation visit, intermediate visits and a closing visit will be organized on site by the monitor in accordance with the monitoring plan.
**Ethics and dissemination**			
Research ethics approval	24	Plans for seeking research ethics committee/institutional review board (REC/IRB) approval	17
Protocol amendments	25	Plans for communicating important protocol modifications (eg, changes to eligibility criteria, outcomes, analyses) to relevant parties (eg, investigators, REC/IRBs, trial participants, trial registries, journals, regulators)	17
Consent or assent	26a	Who will obtain informed consent or assent from potential trial participants or authorised surrogates, and how (see Item 32)	9
	26b	Additional consent provisions for collection and use of participant data and biological specimens in ancillary studies, if applicable	Additional consent will be asked for the use of participants data in future studies
Confidentiality	27	How personal information about potential and enrolled participants will be collected, shared, and maintained in order to protect confidentiality before, during, and after the trial	17
Declaration of interests	28	Financial and other competing interests for principal investigators for the overall trial and each study site	18

For peer review only

Access to data	29	Statement of who will have access to the final trial dataset, and disclosure of contractual agreements that limit such access for investigators	16-17
Ancillary and post-trial care	30	Provisions, if any, for ancillary and post-trial care, and for compensation to those who suffer harm from trial participation	See study record on ClinicalTRials.gov
Dissemination policy	31a	Plans for investigators and sponsor to communicate trial results to participants, healthcare professionals, the public, and other relevant groups (eg, via publication, reporting in results databases, or other data sharing arrangements), including any publication restrictions	17
	31b	Authorship eligibility guidelines and any intended use of professional writers	Authorship eligibility was based on The International Committee of Medical Journal Editors Recommendations for the Conduct, Reporting, Editing, and Publication of Scholarly Work in Medical Journals
	31c	Plans, if any, for granting public access to the full protocol, participant-level dataset, and statistical code	17
**Appendices**			
Informed consent materials	32	Model consent form and other related documentation given to participants and authorised surrogates	The consent form was submitted and approved by the responsible ethics committee. It is available via the BASEC system.
Biological specimens	33	Plans for collection, laboratory evaluation, and storage of biological specimens for genetic or molecular analysis in the current trial and for future use in ancillary studies, if applicable	Not applicable

*It is strongly recommended that this checklist be read in conjunction with the SPIRIT 2013 Explanation & Elaboration for important clarification on the items. Amendments to the protocol should be tracked and dated. The SPIRIT checklist is copyrighted by the SPIRIT Group under the Creative Commons "Attribution-NonCommercial-NoDerivs 3.0 Unported" license.

For peer review only

BMJ Open

Tackling childbirth-related intrusive memories with a single-session behavioural intervention including a visuospatial task: protocol for a single-blind, waitlist-controlled randomised trial

Journal:	*BMJ Open*
Manuscript ID	bmjopen-2023-073874.R1
Article Type:	Protocol
Date Submitted by the Author:	11-May-2023
Complete List of Authors:	Fort, Déborah; University of Lausanne Faculty of Biology and Medicine, Institute of Higher Education and Research in Healthcare<br>Deforges, Camille; University of Lausanne Faculty of Biology and Medicine, Institute of Higher Education and Research in Healthcare<br>Messerli-Bürgy, Nadine; University of Lausanne Faculty of Social and Political Science, Family and Development research center (FADO)<br>Michael, Tanja; Saarland University, Division of Clinical Psychology and Psychotherapy, Department of Psychology<br>Baud, David; Lausanne University Hospital Department of Woman Mother and Child, Materno-Fetal and Obstetrics Research Unit<br>Lalor, Joan; University of Dublin Trinity College, School of Nursing and Midwifery<br>Rimmele, Ulrike; University of Geneva, Emotion and Memory Laboratory, Faculty of Education Sciences and Psychology; University of Geneva, Centre interfacultaire en sciences affectives (CISA)<br>Horsch, Antje; University of Lausanne Faculty of Biology and Medicine, Institute of Higher Education and Research in Healthcare; Lausanne University Hospital Department of Woman Mother and Child, Neonatology Service
**Primary Subject Heading**:	Mental health
Secondary Subject Heading:	Obstetrics and gynaecology
Keywords:	Postpartum Period, Adult psychiatry < PSYCHIATRY, Clinical Trial, Life Change Events, Postpartum Women < Postpartum Period

SCHOLARONE™
Manuscripts

Tackling childbirth-related intrusive memories with a single-session behavioural intervention including a visuospatial task: protocol for a single-blind, waitlist-controlled randomised trial

AUTHORS:

Déborah Fort, MSc[1]

Camille Deforges, PhD[1]

Nadine Messerli-Bürgy, PhD[2]

Tanja Michael, PhD[3]

David Baud, MD-PhD[4]

Joan Lalor, PhD[5]

Ulrike Rimmele, PhD[6,7,8,9]

Antje Horsch, PhD[1,10]

AFFILIATIONS:

1 Institute of Higher Education and Research in Healthcare, Faculty of Biology and Medicine, University of Lausanne, Lausanne, Switzerland

2 Family and Development research center (FADO), Faculty of Social and Political Sciences, University of Lausanne, Lausanne, Switzerland

3 Division of Clinical Psychology and Psychotherapy, Saarland University, Saarbrücken, Germany

4 Materno-Fetal and Obstetrics Research Unit, Department Woman-Mother-Child, Lausanne University Hospital, Lausanne, Switzerland

5 School of Nursing and Midwifery, Trinity College Dublin, Dublin 2, Ireland

6 Emotion and Memory Laboratory, Faculty of Education Sciences and Psychology, University of Geneva, Geneva, Switzerland

For peer review only

[7] Centre interfacultaire de gérontologie et d'études des vulnérabilités (CIGEV), University of Geneva, Switzerland

[8] Centre interfacultaire en sciences affectives (CISA), University of Geneva, Switzerland

[9] Geneva University Neurocenter, Switzerland

[10] Neonatology Service, Department Woman-Mother-Child, Lausanne University Hospital, Lausanne, Switzerland

Correspondence to:

Antje Horsch

Institute of Higher Education and Research in Healthcare, Faculty of Biology and Medicine, University of Lausanne, Route de la Corniche 10, CH-1010 Lausanne, Switzerland

E-mail: antje.horsch@chuv.ch

WORD COUNT: 4077

For peer review only

ABSTRACT

Introduction

Approximately 12.3% of mothers experience childbirth-related posttraumatic stress symptoms (CB-PTSS). However, evidence-based interventions to treat CB-PTSS are lacking. Intrusive memories (IM), a key symptom of CB-PTSS, are distressing and can trigger other PTSS by reliving the traumatic event. Emerging evidence shows that a behavioural intervention involving a visuospatial task (BI-VT) can reduce the number of IM and PTSS, supposedly by interfering with the reconsolidation of the trauma memory. This study aims to test the efficacy of a single-session BI-VT targeting IM to reduce the number of childbirth-related (CB-) IM and PTSS, in comparison to a waitlist control group (WCG).

Methods and analysis

In this multicenter, single-blind, randomised controlled trial (RCT) being undertaken at one regional and one university hospital in Switzerland, 60 participants will be allocated to the immediate intervention group (IIG), receiving the immediate intervention on day 15, and 60 participants to the WCG receiving the delayed intervention on day 30. All participants will report their CB-IM during the two weeks pre- and post-immediate intervention in diaries. The IIG will additionally report their CB-IM over weeks five and six post-immediate intervention. Self-report questionnaires will assess CB-PTSS at two weeks pre- and post-immediate intervention in both groups, and at six weeks post-immediate intervention in the IIG. A feedback questionnaire will evaluate the intervention acceptability. The primary outcome will be group differences in the number of CB-IM between the two weeks pre- and post-immediate intervention. Secondary outcomes will be CB-PTSS at two and six weeks post-immediate intervention, the number of CB-IM at weeks five and six post-immediate intervention, and intervention acceptability.

Ethics and dissemination

Ethical approval was granted by the Human Research Ethics Committee of the Canton of Vaud (study number 2022-00652). Participants will provide an informed consent before study participation. Results will be presented in peer-reviewed journals and at conferences.

Trial registration ClinicalTrials.gov, NCT05381155.

KEYWORDS Posttraumatic stress disorder, Childbirth, Behavioural therapy, Memory reconsolidation, Intrusive memories, Intrusions, Visuospatial task, Tetris.

For peer review only

STRENGTHS AND LIMITATIONS OF THIS STUDY

- This study aims to contribute to the development of an innovative, single-session, and easily accessible intervention to reduce childbirth-related posttraumatic stress symptoms (CB-PTSS).
- A pilot study has already shown high efficacy and acceptability of this single-session intervention.
- CB-PTSS will be assessed with a validated questionnaire specifically developed for the perinatal context.
- Participants will be blinded to group allocation, but the research team will not.
- The waitlist control group is not an active comparator.

For peer review only

INTRODUCTION

Childbirth-related post-traumatic stress disorder and intrusive memories

Approximately 12.3% of mothers develop childbirth-related post-traumatic stress symptoms (CB-PTSS) and 4.7% of mothers meet the diagnostic criteria for childbirth-related post-traumatic stress disorder (CB-PTSD) [1]. PTSD is a psychiatric disorder, highly comorbid with anxiety and depression [2], with distressing symptoms including intrusions, avoidance of trauma-related cues, negative alterations in cognitions and mood, and alterations in arousal and reactivity [3]. Intrusive memories (IM), a core symptom of PTSD, are recurrent and involuntary recollections of the trauma memory [4]. IM are vivid, multisensory, predominantly visual, fragments of the trauma, and are highly distressing [5]. CB-PTSS negatively affect various facets of family life, such as the mother-infant relationship, breastfeeding, and child development [6]. Despite their high prevalence, there is currently only limited evidence concerning the effectiveness of interventions to reduce CB-PTSS [7]. Cognitive-behavioural therapy is currently one of the most common evidence-based interventions for PTSD, but 40 to 50% of patients are unresponsive to it [8]. Furthermore, access to such interventions is limited due to costs arising from numerous sessions with highly trained clinicians and lack of such therapists. Thus, it is essential to develop brief, easily accessible, and resource-efficient psychological interventions adapted to the perinatal context.

For such interventions, IM are targets of particular interest since they involve re-experiencing distressing moments of the trauma and may trigger or maintain other PTSS [9]. For example, high frequency of IM is associated with more difficulties in concentration [10]. New interventions targeting IM could therefore reduce not only intrusions but also other (CB-)PTSS, such as avoidance symptoms [11], and may thus be an innovative solution to support traumatised individuals.

Interventions assumed to tackle intrusive memories through interference with memory reconsolidation

Each new event initiates memory consolidation, a time-dependent process during which memories are malleable until their stabilisation into long-term memory within hours [12,13]. Acute emotional experiences (e.g., traumatic events) trigger a physiological stress response that modulates memory consolidation, resulting in particularly strong and lasting memories [14]. Furthermore, in healthy volunteers, physiological stress reactivity during a traumatic film appears to increase the number of IM in the following days [15]. This suggests that emotional arousal linked to traumatic events impacts memory formation, by enhancing the consolidation of distressing memories [14], and finally increase IM.

For peer review only

Although emerging evidence supports the efficacy of interventions assumed to interfere with trauma memory consolidation to prevent the development of PTSS [16], it is not always possible to intervene within the hours following a traumatic event [17]. A growing body of literature supports the assumption that memories are not fixed forever after consolidation, but that they can become malleable again when reactivated, regardless of the time passed since the traumatic event [18,19]. Reactivating a memory makes it malleable again for a few hours, until its re-stabilisation into long-term memory, corresponding to the reconsolidation process [20]. Consequently, memory reconsolidation is hypothesised to be a new window of opportunity to tackle IM [21] and reduce PTSS.

Interventions assumed to interfere with memory (re)consolidation have used various interference strategies, such as pharmacological agents, psychological therapy techniques, or behavioural interventions involving a visuospatial task (BI-VT) [16]. Such visuospatial tasks (e.g., Tetris gameplay), applied after assumes memory reactivation, possibly interfere with the reconsolidation of traumatic images by taxing the same resources needed by the visuospatial working memory to re-stabilise them [21,22]. These tasks appear as the most appropriate in the perinatal context (e.g., no drug contraindications, accessible to any trained clinicians, and minimum exposure to the trauma memory). So far, lab studies support that a visuospatial task delivered after the reactivation of a traumatic film memory leads to a reduction of the number of IM in healthy volunteers [23,24]. Further, a few translational studies reported encouraging results regarding the efficacy of BI-VT on the number of IM and PTSS in clinical samples [25,26]. For example, one BI-VT was applied to participants suffering from complex PTSD in an open-label single case series (n=20) [25]. After repeated sessions, during which participants produced writing narratives of a self-selected IM and then played Tetris for 25 minutes, a mean reduction of 64% of the targeted IM frequency was observed, whereof 16 out of the 20 participants reported an IM frequency reduction of at least 50%. In another study, repeated sessions of a BI-VT including Tetris gameplay also resulted in a decrease of the number of IM of refugees in a small single case series (n=4) [26].

Developing an evidence-based single-session BI-VT adapted to the perinatal context

A single-session BI-VT was recently adapted to the perinatal context in a pre-post pilot study (n=18) [27]. During the intervention, participants briefly narrated their childbirth on the maternity ward where they had given birth, as evidence suggest that being in a similar physical context as the one in which the traumatic event occurred improves the chances of successful memory reactivation [28]. Narration of their childbirth, ensuring a reliable source of the original memory [29], lasted 10 minutes for a sufficient exposure to the reminder cues supposed to engage memory reconsolidation of strong and old memories [30], such as trauma-related memories. Participants then played Tetris during 20 minutes in a neutral room. This single-session BI-VT led to a median reduction of 82% of the number of childbirth-related IM (CB-IM) [27]. Participants also reported an average reduction of 57% of CB-PTSS severity at one-

For peer review only - http://bmjopen.bmj.com/site/about/guidelines.xhtml

month post-intervention and rated the intervention as highly acceptable. Characteristics (distress, nowness, and sensorial modalities) of CB-IM were also described in this study, but a larger sample size is needed to better understand the nature of CB-IM and their responsiveness to such intervention.

These preliminary results suggest that a single-session BI-VT is suitable for the perinatal context. Building on this pilot study, a RCT will then allow to control for other potential confounding factors, such as the time between the trauma and the intervention [31]. Since, to our knowledge, no equivalent single-session intervention aimed at reducing CB-IM exists, a comparative treatment control group was not applicable. Therefore, to test the efficacy of a BI-VT to reduce CB-PTSS and to allow all help-seeking participants to benefit from the likely positive effects of this BI-VT, this RCT will include a waitlist control group as a comparator. As waitlists can create a nocebo condition, which might bias participants' self-reported symptoms [32] and artificially increase the estimated effects of the experimental intervention [33], participants will be blind to group allocation in an effort to limit these biases. Even though this RCT including a waitlist control group will not allow to study the specific effect of the mechanisms of this BI-VT on CB-PTSS in comparison to other interventions, it will bring more evidence to define this BI-VT as potentially *efficacious* to reduce CB-PTSS [31]. Given the lack of evidence supporting current interventions to treat established CB-PTSS [34], developing a single-session, low cost, easily accessible, and evidence-based intervention would be of great clinical value and might reduce long-term difficulties of mothers after traumatic childbirth.

One challenge for the translational research on memory reconsolidation is to identify if each supposed memory process is taking place and at which point [35]. No markers of these memory processes exist so far, but emotional arousal may be used to infer if participants have been sufficiently engaged in the narration of their traumatic memory to trigger memory reactivation. Previous findings revealed that a decrease in emotional arousal after playing Tetris is associated to an increased responsiveness to a similar BI-VT [25]. Therefore, we will include emotional and physiological measures of arousal (e.g., subjective distress and heart rate variability (HRV) [36,37]) to explore conditions enabling a successful implementation of a BI-VT in the perinatal context. In the future, such an assessment of physiological/emotional arousal could guide a personalised adaptation of this type of intervention [38].

Aims of the present study

This study aims to test the efficacy of a single-session BI-VT in reducing the number of CB-IM and CB-PTSS, in comparison to a waitlist. This intervention is assumed to reactivate the childbirth memory and interfere with its reconsolidation, respectively through a brief childbirth narration and Tetris gameplay, leading to a decrease in CB-IM and CB-PTSS. The primary outcome will be the difference in the number of CB-IM between baseline (two weeks pre-immediate intervention) and the two weeks post-immediate intervention in the Immediate Intervention Group (IIG) compared to the Waitlist Control Group (WCG)

For peer review only

(see Figure 1 for the study flowchart). Secondary outcomes will include group differences in CB-PTSS between baseline and at two weeks post-immediate intervention, as well as the acceptability of the intervention assessed in both groups. The following secondary outcomes will concern the IIG: the change in CB-PTSS between baseline and follow-up (at six weeks post-immediate intervention), and the change in the number of CB-IM between baseline and follow-up (weeks five and six post-immediate intervention). In addition, this study will explore, in both groups, the course of the nature (distress, nowness, and sensorial modality) and content of CB-IM before and after the intervention, as well as associations between participants' HRV and subjective distress course during the intervention, and the intervention efficacy. In the IIG, we will additionally explore the course of postnatal depression and anxiety symptoms at six weeks post-immediate intervention. Finally, this study will explore the change in the number CB-IM and CB-PTSS between the two weeks pre- and post-delayed intervention in the WCG.

METHODS AND ANALYSIS

Study design

The present study will be a single-blind, waitlist-controlled RCT. The trial will include two arms: the IIG receiving the immediate intervention on day 15, and the WCG receiving the same intervention on day 30 (delayed intervention).

Population, recruitment, group allocation, and blinding

Mothers with a minimum age of 18 years, who gave birth at least six weeks prior to the screening in one of the two study centres (one university hospital, being the main study centre, and one regional hospital from Switzerland), and suffer from CB-IM will be eligible to participate. To avoid a floor effect regarding CB-IM reduction, we will only include mothers reporting at least four CB-IM over the two weeks prior to the screening. Exclusion criteria will be: insufficient French-speaking level to complete the assessments, established intellectual disability or psychotic illness, an ongoing psychological treatment in relation to childbirth, propranolol medication (due to suspected effects on memory reconsolidation [39]), alcohol and/or illicit drug abuse, being pregnant, perinatal loss of a child born during the index traumatic childbirth (i.e. the childbirth triggering the CB-IM targeted during the intervention), or a life-threatening illness of the mother or infant(s). Moreover, mothers not able to distinguish potential IM related to another traumatic event from CB-IM linked to the index traumatic childbirth will be excluded. Importantly, data related to any participants that reported less than two CB-IM at baseline will be excluded from relevant analyses to avoid a floor effect, or if they stated having a concurrent psychological treatment in relation to childbirth during study participation.

For peer review only

Adverts will be displayed in nurseries and practices of midwives, gynaecologists, and paediatricians. Study information will also be shared on social media. In addition, study centres will send a text message to invite potentially eligible mothers who gave birth in their hospitals to participate in the study.

During a phone call, the study coordinator (DF) will screen interested mothers. The presence and number of CB-IM over the past two weeks will be assessed with the item B1 of the Clinician-administered PTSD scale for Diagnostic and Statistical Manual of Mental Disorders, Fifth Edition, (DSM-5) [40], and the T-ACE will be used to assess alcohol consumption [41]. Eligible mothers who signed the consent form will be randomly allocated to the IIG or WCG with a 1:1 ratio. To ensure concealment, a scientific collaborator not involved in data collection created a block randomisation list stratified by centre, which will be automatically generated by the Research Electronic Data Capture (REDCap) software. To limit waitlist condition-related bias on participants' self-reports [32,33], participants will be blind to group allocation. To that matter, participants will only be informed that both groups will complete the same assessments and receive the intervention, but in a different order. For an overview of study procedures for both groups, see Figure 1. To improve completion of study assessments, the study coordinator will follow participants' progression during the whole study and participants will receive reminders to complete questionnaires.

Study procedures

T1-Baseline

All participants will receive questionnaires regarding sociodemographic and obstetric information, postnatal depression and anxiety symptoms on the first day of the baseline. They will report their CB-IM in diaries during the two weeks pre-immediate intervention. They will also report their CB-PTSS in a self-report questionnaire during week 2.

T2-Immediate intervention

The IIG will receive the intervention on day 15. For a description of intervention procedures, see the intervention section.

T3-Post-immediate intervention

Both groups will report their CB-IM during the two weeks post-immediate intervention. To ensure blinding of WCG, this group will complete a diary covering T1 and T3. Both groups will report their CB-PTSS in a self-report questionnaire during week 2 post-immediate intervention.

T4-Delayed intervention

For peer review only

The WCG will receive the delayed intervention on day 30. The delayed intervention follows the exact same procedures as the immediate intervention.

T5-Post-delayed intervention

The WCG will report their CB-IM in a diary during the two weeks post-delayed intervention and their CB-PTSS in a self-report questionnaire during week 2 post-delayed intervention.

T6-Follow-up

The IIG will report their CB-IM in a diary during weeks five and six post-immediate intervention. They will also complete self-report questionnaire assessing postnatal depression and anxiety symptoms, as well as CB-PTSS during week 6 post-immediate intervention. The WCG will not complete follow-up assessments to ensure a fair distribution of burden between groups

T7-Feedback phone call

Both groups will complete a feedback questionnaire by phone.

Intervention

The study coordinator, a trained psychologist, will meet the participant in a neutral room, inside a non-medical administrative building, explain intervention procedures, and attach an electrocardiogram (ECG) device to their chest. To assess HRV baseline, the participant will count aloud during two minutes (Count aloud task A). The study coordinator and the participant will then go into a gynaecological examination room of the maternity ward to realise the first part of the intervention:

1. Childbirth narrative task (assumed memory reactivation phase): The participant will be asked to briefly narrate their childbirth orally during 10 minutes. The study coordinator will first ask participants to narrate the overall course of their childbirth during five to seven minutes (Narrative task A). Then, the participant will be asked to narrate the moment associated to the most frequent or distressing CB-IM reported in the diary, for another three to five minutes (Narrative task B). If the participant perceived her distress to be overwhelming, she will have the option to go into another room not located in the maternity or to write about her childbirth instead of narrating it orally.

The participant and the study coordinator will then return to the neutral room to start the second part of the intervention, at least ten minutes later, in order to allow the assumed memory labilisation [42] and to avoid further memory reactivation during Tetris gameplay:

For peer review only

2. Tetris gameplay (assumed memory reconsolidation disruption phase): The study coordinator will ask the participant to play Tetris on a handheld gaming device (Nintendo 3DS), in "Marathon" mode, with both sound and 3D switched off. The participant will be instructed to focus both on the block currently falling and on the upcoming blocks (shown in a preview box). To promote mental rotation, participants will be encouraged to use their imagination to visualise how to place each block to complete as many horizontal lines as possible [24]. The participant will have a three-minute practice trial before playing Tetris for 20 minutes uninterrupted. The study coordinator will stay in the room to ensure that the participant will stay focused on Tetris during the 20 minutes-long gameplay (e.g., instead of looking at her phone).

After Tetris gameplay, the participant will count aloud for two minutes to measure HRV recovery (Count aloud task B). Finally, the participant will be asked not to play Tetris or to research the therapeutic use of Tetris until she terminated the study.

An overview of the intervention procedures is shown in Figure 2.

Primary outcome

The primary outcome will be the difference in the number of CB-IM between baseline and the two weeks post-immediate intervention across groups.

Secondary outcomes

Secondary outcomes will be:

- The change in CB-PTSS between: a) baseline and at two weeks post-immediate intervention across groups, b) baseline and follow-up in the IIG.
- The change in the number of CB-IM between baseline and follow-up in the IIG.
- The acceptability of the intervention in both groups

Exploratory outcomes

This study will investigate the following exploratory outcomes in both groups:

- The course of the distress, nowness, sensorial modalities, and content related to CB-IM before and after the intervention.
- The association of HRV and subjective distress assessed during the different phases of the intervention, with the intervention efficacy (number of CB-IM and CB-PTSS post-intervention).

In addition, exploratory outcomes will include in the IIG:

For peer review only

- The change in postnatal depression, and anxiety symptoms between baseline and follow-up.
- The change in the number of CB-IM between the two weeks post-immediate intervention and follow-up.
- The change in CB-PTSS between two weeks post-immediate intervention and follow-up.

We will also explore, in the WCG, the change in the number of CB-IM and CB-PTSS between the two weeks pre- and post-delayed intervention.

Data collection

Table 1 provides an overview of study outcomes and related timepoints.

For peer review only

Table 1. Overview of study outcomes and timepoints

Variables	Instruments	Study period						
		**T1** Baseline	**T2** Immediate intervention	**T3** Post-immediate intervention	**T4** Delayed intervention	**T5** Post-delayed intervention	**T6** Follow-up	**T7** Feedback phone call
CB-IM	Diaries	IIG + WCG		IIG + WCG		WCG	IIG	
Content of CB-IM		IIG + WCG		IIG + WCG		WCG	IIG	
Distress of CB-IM		IIG + WCG		IIG + WCG		WCG	IIG	
Nowness of CB-IM		IIG + WCG		IIG + WCG		WCG	IIG	
Sensorial modalities of CB-IM		IIG + WCG		IIG + WCG		WCG	IIG	
CB-PTSS	City Birth Trauma Scale [43]	IIG + WCG		IIG + WCG		WCG	IIG	
Anxiety	Hospital Anxiety and Depression scale – Anxiety subscale [44]	IIG + WCG					IIG	
Postnatal depression	Edinburgh Postnatal Depression Scale [45]	IIG + WCG					IIG	
Heart rate variability	Firstbeat Bodyguard 2 ECG device		IIG		WCG			
Subjective distress	Visual Analogue Scales		IIG		WCG			
Intervention acceptability	Feedback questionnaire III							IIG + WCG
Sociodemographic variables	Sociodemographic questionnaire	IIG + WCG						
Obstetric and medical data	Medical records & self-report questionnaire	IIG + WCG						

Note: IIG, immediate intervention group; WCG, waitlist control group; CB-IM, childbirth-related intrusive memories; CB-PTSS, Childbirth-related posttraumatic stress symptoms; ECG, Electrocardiogram; T1; Weeks 1 & 2 pre-immediate intervention; T2, Day 15; T3, Weeks 1 & 2 post-immediate intervention; T4, Day 30; T5, Weeks 1 & 2 post-delayed intervention; T6, Weeks 5 & 6 post-immediate intervention.

Measures

1. Childbirth-related posttraumatic stress symptoms

1.1. Diaries

For each CB-IM, participants will be asked to write in a diary the time it occurred, its content, and to report, from 0=*not at all* to 10=*extremely*, 1) how distressing it was, 2) to what extend they had the impression that the memory was happening here and now (*nowness* [46]), and 3) its sensorial modalities. CB-IM will be defined to participants as "*involuntary memories of the labour or childbirth that come to mind without warning. They can be vivid and emotional, and are often like a mental image, such as a photograph or film. They can involve all the senses (…). They can be very short, elusive and fragmented.*" [27]. Participants will use electronic diaries hosted in REDCap, or a paper version (e.g., if not able to use the electronic ones), adapted from previous work [27].

1.2. City Birth Trauma Scale (CBTS)

This questionnaire assesses CB-PTSS during the past week, and diagnostic criteria of PTSD according to the DSM-5 [43]. Twenty-two items, using a 4-point Likert scale, assess the four CB-PTSS clusters (*re-experiencing*, *avoidance*, *negative cognitions and mood*, *hyperarousal*). Original instructions of the CBTS will be adapted for participants who had multiple childbirth(s) to complete the questionnaire regarding the index traumatic childbirth. Good psychometric properties were found for the French version of the CBTS [47].

2. Anxiety and depression symptoms

2.1. Anxiety subscale of the Hospital Anxiety and Depression Scale (HADS-A)

This subscale assess anxiety symptoms and consists of seven items using 4-point Likert scales [44]. Good psychometric properties are reported for the French version of the HADS [48].

2.2. Edinburgh Postnatal Depression Scale (EPDS)

This 10-item questionnaire measures postnatal depression symptoms during the past week with 4-point Likert scales [45]. The French version of the EPDS has good psychometric properties [49].

3. Intervention-related measures

3.1. Intervention acceptability

For peer review only

Participants will answer the Feedback Questionnaire III assessing intervention acceptability, usefulness, and expectations regarding its effects. These questions, based on previous work [27], have been designed to best match the intervention characteristics.

3.2. Heart rate variability

Participants' HRV will be recorded using a Firstbeat Bodyguard 2 (Firstbeat Technologies Ltd., Jyväskylä, Finland) ECG device during the whole intervention procedures. Count aloud tasks A & B will measure HRV baseline and recovery, in order to compare participants HRV during Narrative tasks A & B, and Tetris gameplay.

3.3. Subjective distress

Visual analogue scales will be presented to participants at 11 timepoints during the intervention to assess their subjective distress level from 0=*not stressed and/or anxious at all* to 10=*extremely stressed and/or anxious*.

4. Sociodemographic and obstetric characteristics

*4.1. Sociodemographic variable*s

A sociodemographic questionnaire will assess participants' age, nationality, marital status, education level, and trauma history.

4.2. Obstetric and medical data

Obstetric and medical data will be extracted from participants' medical record, such as childbirth mode, gestational age, and Apgar scores. Birth-related information will also be collected at baseline, through a self-report questionnaire (e.g., presence of a relative, perceived threat for self or infant).

5. Other variables

This study includes other outcomes, aiming to specify the effects of the intervention, presented in Table 2.

For peer review only

Table 2. Other outcomes			
**Variables**	**Instruments**	**Timepoints**	**Description**
Cognitive flexibility	Cognitive flexibility scale [50]	T1	Cognitive flexibility defines, inter alia, the capacity to adapt to the situation and being flexible [50].
Cue specificity	Feedback questionnaires I & III	T2/T4, T7	These questionnaires will assess the similarity between childbirth narration and the original childbirth memory.
Context specificity	Feedback questionnaires II & III	T3/T5, T7	Questions will assess the similarity between the intervention context and the original traumatic context.
Diary compliance	Diaries	T1, T3, T5, T6	Diary compliance will check the extent to which participants were able/remembered to report their CB-IM at the end of each diary.
HRV control variables	Questionnaire on HRV control variables	T1, T2/T4	This questionnaire will contain control variables regarding the analyses of the HRV (e.g., weight, height, medicine use, cigarettes and alcohol consumption).
Video games use	Feedback questionnaires I & III	T2/T4, T7	Questions will assess participants' video games use (e.g., engagement in the game during the intervention, Tetris perceived difficulty, video games habits, and Tetris use post-intervention).
Sleep	Sleep questionnaire	T3/T5	This questionnaire will evaluate the number of hours of sleep during the 24 hours following the intervention.
Concurrent psychological treatment	Feedback questionnaire III	T7	A question will check the absence of a concurrent psychological treatment in relation to the index childbirth during study participation.

Note: CB-IM, childbirth-related intrusive memories; HRV, heart rate variability. T1; Weeks 1 & 2 pre-immediate intervention; T2, Day 15; T3, Weeks 1 & 2 post-immediate intervention; T4, Day 30; T5, Weeks 1 & 2 post-delayed intervention; T6, Weeks 5 & 6 post-immediate intervention; T7, Feedback phone call.

Sample size calculation

A sample size calculation was conducted for our primary and secondary outcomes based on the results obtained from the pilot study [27], assessing the efficacy of the same intervention using a single-group pre-post design, and from laboratory studies using RCT designs to assess the efficacy of a similar BI-VT [23,24]. A sample size of 90 participants (45 allocated in each group) is estimated to be necessary to detect the effect of the intervention on our primary and secondary outcomes with an 80% power (α = 0.05). Based on our experience with this study population [11,27], a conservative estimate of a 30% drop-out and exclusion was assumed. We therefore plan to recruit 120 participants in total.

Data management and statistical analysis

Participants' entries in electronic diaries and questionnaires will be directly stored in the REDCap software. The research staff will enter manually the rest of the data collected during the screening, the

For peer review only

intervention, and paper diaries in REDCap. All data will be hosted on a high security server of the Lausanne University Hospital performing automatic continuous backups. All primary outcome data and a random 5% of the rest of the data will be double-checked. Independent and blind coders will inspect diary entries to detect any non-compliance with instructions and count the number of CB-IM. A blind statistician will perform the analyses. To ensure blinding of the coders and the statisticians, they will receive a dataset not indicating which allocation group corresponds to the IIG or WCG. Only the study team will have access to the full coded trial dataset.

For primary outcomes, group differences regarding change of the number of CB-IM between baseline and the two weeks post-immediate intervention will be analysed using repeated-measures analyses (intention-to-treat analyses). For the secondary outcomes, repeated-measures analyses will be performed to analyse group differences concerning CB-PTSS between baseline and at two weeks post-immediate intervention. Logistic regression analyses will also compare the proportion of participants meeting the diagnostic criteria for CB-PTSD across time and groups. Furthermore, repeated measures analyses will serve to analyse change in the number of CB-IM and CB-PTSS between baseline and follow-up in the IIG. Group differences regarding the proportion of participants meeting the diagnostic criteria for CB-PTSD will be analysed with logistic regression analyses. The mean score of the intervention acceptability will be calculated across both groups. Additional post-hoc analyses conducted will be described as such in publications. Missing data will be dealt with using multiple imputation procedures, if appropriate.

Safety

Any serious adverse event occurring during data collection will be documented and reported to the competent ethics committee.

Patient and public involvement

Participants will evaluate the intervention acceptability and will be able to give feedback on the intervention. Each participant will receive a personalised debriefing session. Study results will be sent to participants and shared with the public via social media and public events.

ETHICS AND DISSEMINATION

Ethical approval of the current version of this protocol (dated 1st of December 2022), was granted by the Human Research Ethics Committee of the Canton de Vaud (study number 2022-00652). The study sponsor is the Bureau du Promoteur de Recherche of the Lausanne University Hospital. Participants will provide an informed consent prior to study participation (see Supplemental Material). The study will be monitored by an independent and certified data monitoring body. An initiation, intermediate, and a closing visit will be realised on site by the independent study monitor. A scientific steering committee

For peer review only

is consulted regularly for scientific decisions regarding the conduct of the study. This protocol was registered at ClinicalTrials.gov (NCT05381155) prior to data collection, in May 2022. Results of this study will be submitted for publication to peer-reviewed journals and presented in relevant conferences to ensure dissemination in the scientific community. Furthermore, data will be shared in an anonymised form, at the time of the publication of the related outcomes, in an open access register.

*** ***

CONTRIBUTORS

This protocol was designed by DF, CD and AH. DF, CD and AH drafted the current manuscript. Each author (DF, CD, NMB, TM, DB, JL, UR and AH) contributed to the revision and implementation of study procedures. All authors critically revised the manuscript and approved the final version of the manuscript. Authorship eligibility was based on the Recommendations for the Conduct, Reporting, Editing, and Publication of Scholarly Work in Medical Journals.

FUNDING

This study is supported by a project grant from the Swiss National Science Foundation (SNF IZCOZ0_205434/1).

COMPETING INTERESTS

None declared.

REFERENCES

1. Heyne CS, Kazmierczak M, Souday R, et al. Prevalence and risk factors of birth-related posttraumatic stress among parents: A comparative systematic review and meta-analysis. *Clin Psychol Rev* 2022;94:102157. doi: 10.1016/j.cpr.2022.102157 [published Online First: 2022/05/19]
2. Morland L, Goebert D, Onoye J, et al. Posttraumatic stress disorder and pregnancy health: preliminary update and implications. *Psychosomatics* 2007;48(4):304-8. doi: 10.1176/appi.psy.48.4.304 [published Online First: 2007/06/30]
3. American Psychiatric Association. Diagnostic and statistical manual of mental disorders (5th ed.). Washington, DC: American Psychiatric Publishing 2013.
4. Ehlers A, Clark DM. A cognitive model of posttraumatic stress disorder. *Behav Res Ther* 2000;38(4):319-45. doi: 10.1016/s0005-7967(99)00123-0
5. Ehlers A, Hackmann A, Michael T. Intrusive re-experiencing in post-traumatic stress disorder: phenomenology, theory, and therapy. *Memory* 2004;12(4):403-15. doi: 10.1080/09658210444000025 [published Online First: 2004/10/19]
6. Cook N, Ayers S, Horsch A. Maternal posttraumatic stress disorder during the perinatal period and child outcomes: A systematic review. *J Affect Disord* 2018;225:18-31. doi: 10.1016/j.jad.2017.07.045 [published Online First: 2017/08/05]

For peer review only

7. Furuta M, Horsch A, Ng ESW, et al. Effectiveness of Trauma-Focused Psychological Therapies for Treating Post-traumatic Stress Disorder Symptoms in Women Following Childbirth: A Systematic Review and Meta-Analysis. *Front Psychiatry* 2018;9:591-91. doi: 10.3389/fpsyt.2018.00591
8. Loerinc AG, Meuret AE, Twohig MP, et al. Response rates for CBT for anxiety disorders: Need for standardized criteria. *Clin Psychol Rev* 2015;42:72-82. doi: 10.1016/j.cpr.2015.08.004 [published Online First: 2015/09/01]
9. Iyadurai L, Visser RM, Lau-Zhu A, et al. Intrusive memories of trauma: A target for research bridging cognitive science and its clinical application. *Clin Psychol Rev* 2019;69:67-82. doi: 10.1016/j.cpr.2018.08.005 [published Online First: 2018/10/09]
10. Holmes EA, Ghaderi A, Eriksson E, et al. 'I Can't Concentrate': A Feasibility Study with Young Refugees in Sweden on Developing Science-Driven Interventions for Intrusive Memories Related to Trauma. *Behav Cogn Psychother* 2017;45(2):97-109. doi: 10.1017/s135246581600062x [published Online First: 2017/02/24]
11. Horsch A, Vial Y, Favrod C, et al. Reducing intrusive traumatic memories after emergency caesarean section: A proof-of-principle randomized controlled study. *Behav Res Ther* 2017;94:36-47. doi: 10.1016/j.brat.2017.03.018 [published Online First: 2017/04/30]
12. Nadel L, Hupbach A, Gomez R, et al. Memory formation, consolidation and transformation. *Neurosci Biobehav Rev* 2012;36(7):1640-5. doi: 10.1016/j.neubiorev.2012.03.001 [published Online First: 20120324]
13. McGaugh JL. Memory--a century of consolidation. *Science* 2000;287(5451):248-51. doi: 10.1126/science.287.5451.248 [published Online First: 2000/01/15]
14. McGaugh JL. The amygdala modulates the consolidation of memories of emotionally arousing experiences. *Annu Rev Neurosci* 2004;27:1-28. doi: 10.1146/annurev.neuro.27.070203.144157
15. Schultebraucks K, Rombold-Bruehl F, Wingenfeld K, et al. Heightened biological stress response during exposure to a trauma film predicts an increase in intrusive memories. *J Abnorm Psychol* 2019;128(7):645-57. doi: 10.1037/abn0000440 [published Online First: 2019/07/10]
16. Astill Wright L, Horstmann L, Holmes EA, et al. Consolidation/reconsolidation therapies for the prevention and treatment of PTSD and re-experiencing: a systematic review and meta-analysis. *Transl Psychiatry* 2021;11(1):453. doi: 10.1038/s41398-021-01570-w [published Online First: 2021/09/05]
17. Phelps EA, Hofmann SG. Memory editing from science fiction to clinical practice. *Nature* 2019;572(7767):43-50. doi: 10.1038/s41586-019-1433-7 [published Online First: 2019/08/02]
18. Agren T. Human reconsolidation: a reactivation and update. *Brain Res Bull* 2014;105:70-82. doi: 10.1016/j.brainresbull.2013.12.010 [published Online First: 2014/01/09]
19. Misanin JR, Miller RR, Lewis DJ. Retrograde amnesia produced by electroconvulsive shock after reactivation of a consolidated memory trace. *Science* 1968;160(3827):554-5. doi: 10.1126/science.160.3827.554
20. Lee JLC, Nader K, Schiller D. An Update on Memory Reconsolidation Updating. *Trends Cogn Sci* 2017;21(7):531-45. doi: 10.1016/j.tics.2017.04.006 [published Online First: 20170508]
21. Visser RM, Lau-Zhu A, Henson RN, et al. Multiple memory systems, multiple time points: how science can inform treatment to control the expression of unwanted emotional memories. *Philos Trans R Soc Lond B Biol Sci* 2018;373(1742) doi: 10.1098/rstb.2017.0209
22. Holmes EA, James EL, Coode-Bate T, et al. Can playing the computer game "Tetris" reduce the build-up of flashbacks for trauma? A proposal from cognitive science. *PLoS One* 2009;4(1):e4153. doi: 10.1371/journal.pone.0004153 [published Online First: 2009/01/08]
23. Kessler H, Schmidt AC, James EL, et al. Visuospatial computer game play after memory reminder delivered three days after a traumatic film reduces the number of intrusive memories of the experimental trauma. *J Behav Ther Exp Psychiatry* 2020;67:101454. doi: 10.1016/j.jbtep.2019.01.006 [published Online First: 2019/05/01]

For peer review only

24. James EL, Bonsall MB, Hoppitt L, et al. Computer Game Play Reduces Intrusive Memories of Experimental Trauma via Reconsolidation-Update Mechanisms. *Psychol Sci* 2015;26(8):1201-15. doi: 10.1177/0956797615583071 [published Online First: 2015/07/03]
25. Kessler H, Holmes EA, Blackwell SE, et al. Reducing intrusive memories of trauma using a visuospatial interference intervention with inpatients with posttraumatic stress disorder (PTSD). *J Consult Clin Psychol* 2018;86(12):1076-90. doi: 10.1037/ccp0000340 [published Online First: 2018/12/07]
26. Kanstrup M, Kontio E, Geranmayeh A, et al. A single case series using visuospatial task interference to reduce the number of visual intrusive memories of trauma with refugees. *Clin Psychol Psychother* 2021;28(1):109-23. doi: 10.1002/cpp.2489 [published Online First: 2020/06/12]
27. Deforges C, Fort D, Stuijfzand S, et al. Reducing childbirth-related intrusive memories and PTSD symptoms via a single-session behavioural intervention including a visuospatial task: A proof-of-principle study. *J Affect Disord* 2022;303:64-73. doi: 10.1016/j.jad.2022.01.108 [published Online First: 2022/02/03]
28. Hupbach A, Hardt O, Gomez R, et al. The dynamics of memory: context-dependent updating. *Learn Mem* 2008;15(8):574-9. doi: 10.1101/lm.1022308 [published Online First: 2008/08/08]
29. Debiec J, Diaz-Mataix L, Bush DE, et al. The selectivity of aversive memory reconsolidation and extinction processes depends on the initial encoding of the Pavlovian association. *Learn Mem* 2013;20(12):695-9. doi: 10.1101/lm.031609.113 [published Online First: 2013/11/21]
30. Suzuki A, Josselyn SA, Frankland PW, et al. Memory reconsolidation and extinction have distinct temporal and biochemical signatures. *J Neurosci* 2004;24(20):4787-95. doi: 10.1523/JNEUROSCI.5491-03.2004 [published Online First: 2004/05/21]
31. Chambless DL, Hollon SD. Defining empirically supported therapies. *J Consult Clin Psychol* 1998;66(1):7-18. doi: 10.1037//0022-006x.66.1.7 [published Online First: 1998/03/07]
32. Furukawa TA, Noma H, Caldwell DM, et al. Waiting list may be a nocebo condition in psychotherapy trials: a contribution from network meta-analysis. *Acta Psychiatr Scand* 2014;130(3):181-92. doi: 10.1111/acps.12275 [published Online First: 20140404]
33. Faltinsen E, Todorovac A, Staxen Bruun L, et al. Control interventions in randomised trials among people with mental health disorders. *Cochrane Database Syst Rev* 2022;4(4):MR000050. doi: 10.1002/14651858.MR000050.pub2 [published Online First: 2022/04/05]
34. de Bruijn L, Stramrood CA, Lambregtse-van den Berg MP, et al. Treatment of posttraumatic stress disorder following childbirth. *J Psychosom Obstet Gynaecol* 2020;41(1):5-14. doi: 10.1080/0167482X.2019.1593961 [published Online First: 2019/06/06]
35. Vaverkova Z, Milton AL, Merlo E. Retrieval-Dependent Mechanisms Affecting Emotional Memory Persistence: Reconsolidation, Extinction, and the Space in Between. *Front Behav Neurosci* 2020;14:574358. doi: 10.3389/fnbeh.2020.574358 [published Online First: 2020/11/03]
36. Halligan SL, Michael T, Wilhelm FH, et al. Reduced heart rate responding to trauma reliving in trauma survivors with PTSD: correlates and consequences. *J Trauma Stress* 2006;19(5):721-34. doi: 10.1002/jts.20167 [published Online First: 2006/11/01]
37. Haag K, Hiller R, Peyk P, et al. A Longitudinal Examination of Heart-Rate and Heart Rate Variability as Risk Markers for Child Posttraumatic Stress Symptoms in an Acute Injury Sample. *J Abnorm Child Psychol* 2019;47(11):1811-20. doi: 10.1007/s10802-019-00553-2 [published Online First: 2019/05/11]
38. Kindt M, Elsey JWB. A paradigm shift in the treatment of emotional memory disorders: Lessons from basic science. *Brain Res Bull* 2022;192:168-74. doi: 10.1016/j.brainresbull.2022.11.019 [published Online First: 2022/11/29]
39. Giustino TF, Fitzgerald PJ, Maren S. Revisiting propranolol and PTSD: Memory erasure or extinction enhancement? *Neurobiol Learn Mem* 2016;130:26-33. doi: 10.1016/j.nlm.2016.01.009 [published Online First: 2016/01/26]

For peer review only

40. Blake DD, Weathers FW, Nagy LM, et al. The development of a Clinician-Administered PTSD Scale. *J Trauma Stress* 1995;8(1):75-90. doi: 10.1007/BF02105408 [published Online First: 1995/01/01]
41. Sokol RJ, Martier SS, Ager JW. The T-ACE questions: practical prenatal detection of risk-drinking. *Am J Obstet Gynecol* 1989;160(4):863-8; discussion 68-70. doi: 10.1016/0002-9378(89)90302-5 [published Online First: 1989/04/01]
42. Agren T, Engman J, Frick A, et al. Disruption of reconsolidation erases a fear memory trace in the human amygdala. *Science* 2012;337(6101):1550-2. doi: 10.1126/science.1223006 [published Online First: 2012/09/22]
43. Ayers S, Wright DB, Thornton A. Development of a Measure of Postpartum PTSD: The City Birth Trauma Scale. *Front Psychiatry* 2018;9:409. doi: 10.3389/fpsyt.2018.00409 [published Online First: 20180918]
44. Zigmond AS, Snaith RP. The hospital anxiety and depression scale. *Acta Psychiatr Scand* 1983;67(6):361-70. doi: 10.1111/j.1600-0447.1983.tb09716.x [published Online First: 1983/06/01]
45. Cox JL, Holden JM, Sagovsky R. Detection of postnatal depression. Development of the 10-item Edinburgh Postnatal Depression Scale. *Br J Psychiatry* 1987;150:782-6. doi: 10.1192/bjp.150.6.782 [published Online First: 1987/06/01]
46. Michael T, Ehlers A, Halligan SL, et al. Unwanted memories of assault: what intrusion characteristics are associated with PTSD? *Behav Res Ther* 2005;43(5):613-28. doi: 10.1016/j.brat.2004.04.006 [published Online First: 2005/05/04]
47. Sandoz V, Hingray C, Stuijfzand S, et al. Measurement and conceptualization of maternal PTSD following childbirth: Psychometric properties of the City Birth Trauma Scale-French Version (City BiTS-F). *Psychol Trauma* 2022;14(4):696-704. doi: 10.1037/tra0001068 [published Online First: 2021/07/23]
48. Untas A, Aguirrezabal M, Chauveau P, et al. [Anxiety and depression in hemodialysis: validation of the Hospital Anxiety and Depression Scale (HADS)]. *Nephrol Ther* 2009;5(3):193-200. doi: 10.1016/j.nephro.2009.01.007 [published Online First: 2009/04/07]
49. Guedeney N, Fermanian J. Validation study of the French version of the Edinburgh Postnatal Depression Scale (EPDS): new results about use and psychometric properties. *European Psychiatry* 2020;13(2):83-89. doi: 10.1016/s0924-9338(98)80023-0
50. Martin MM, Rubin RB. A New Measure of Cognitive Flexibility. *Psychological Reports* 2016;76(2):623-26. doi: 10.2466/pr0.1995.76.2.623

Figure titles/legends

Figure 1. Study flowchart

CB-PTSS, childbirth-related posttraumatic stress symptoms: CB-IM, childbirth-related intrusive memories.

Figure 2. Intervention procedures

Adapted from Deforges et al. [27], with permission.

For peer review only

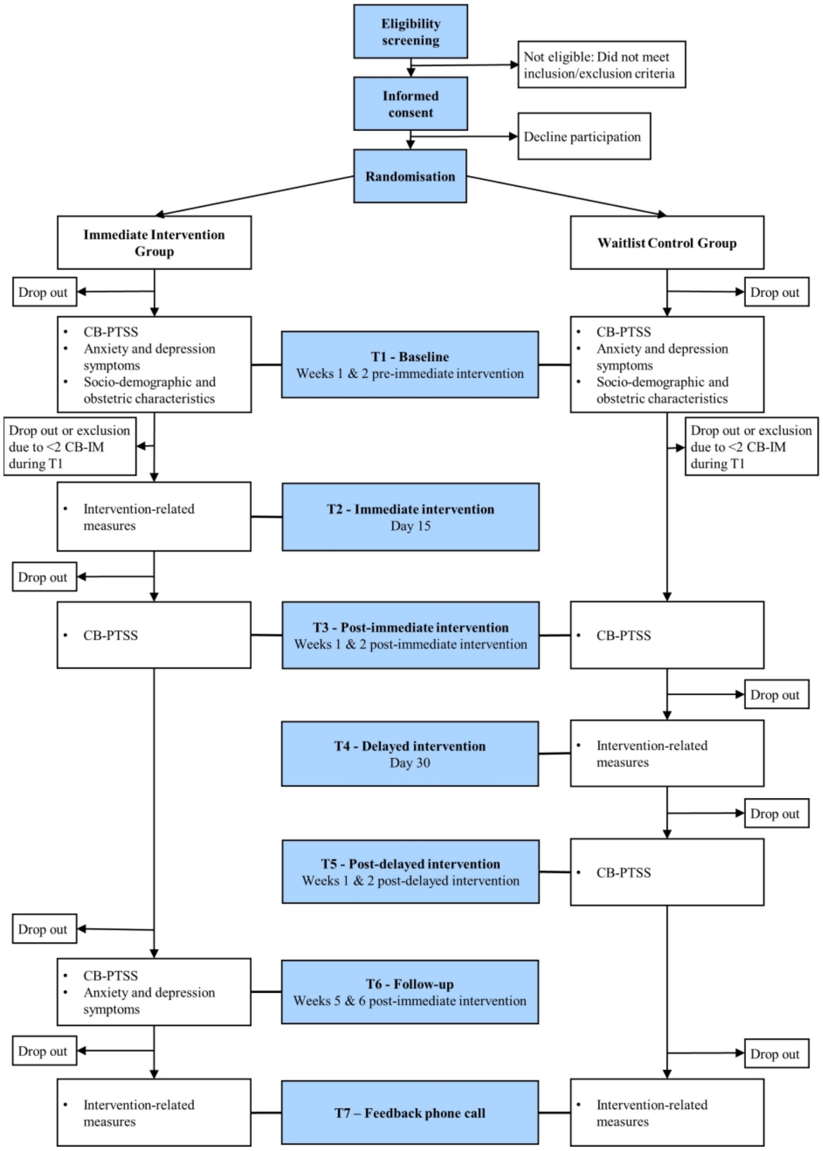

Figure 1. Flowchart of study procedures. Note: CB-PTSS, childbirth-related posttraumatic stress symptoms: CB-IM, childbirth-related intrusive memories.

190x275mm (300 x 300 DPI)

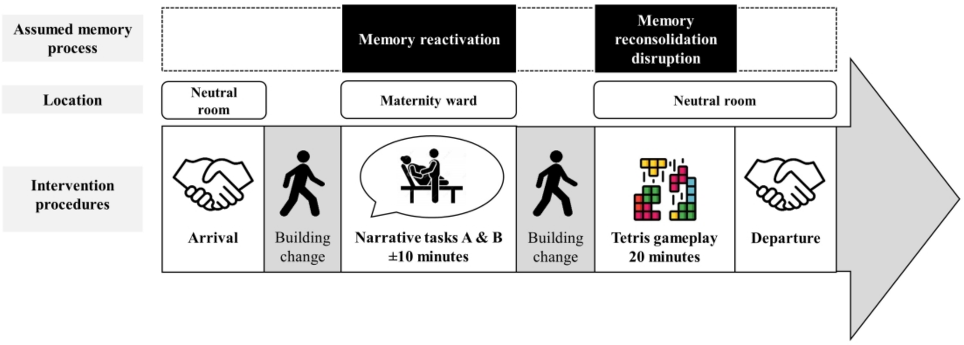

Figure 2. Intervention procedures. Adapted from Deforges et al. [27] with permission.

284x100mm (300 x 300 DPI)

Unil UNIL | Université de Lausanne
Institut universitaire de formation et de recherche en soins FBM
CHUV
FNSNF
FONDS NATIONAL SUISSE
SCHWEIZERISCHER NATIONALFONDS
FONDO NAZIONALE SVIZZERO
SWISS NATIONAL SCIENCE FOUNDATION

Étude ASTRAL : Lutter contre les souvenirs intrusifs après un accouchement difficile

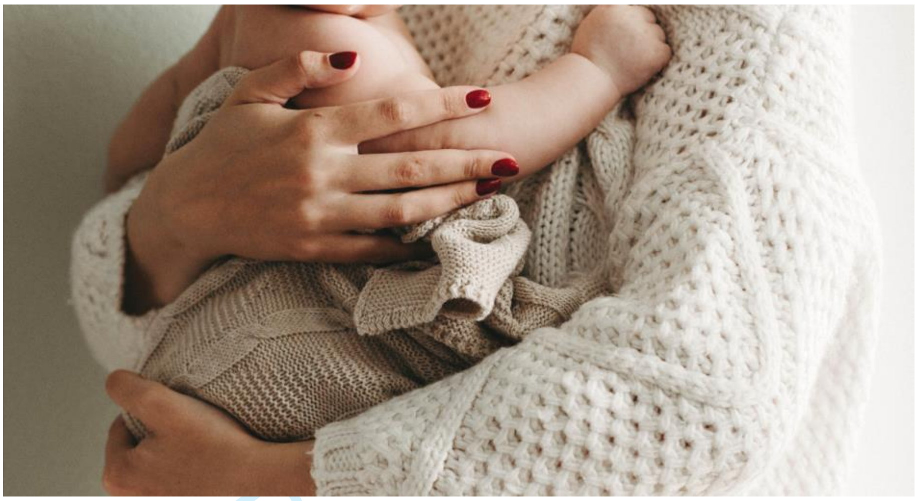

Chère Madame,

Merci beaucoup pour votre intérêt pour notre projet de recherche ! Comme expliqué au téléphone, nous aimerions vous proposer de participer à notre projet de recherche. Ce projet est développé au sein du groupe de recherche périnatale de Lausanne, qui est rattaché au CHUV et à l'Université de Lausanne. Ce feuillet d'information décrit le projet de recherche de façon détaillée. Avant de décider d'y participer, il est important que vous compreniez le but de cette recherche, ainsi que ses enjeux et ce que cela implique pour vous. C'est pourquoi nous vous prions de parcourir attentivement ce document. Votre participation est entièrement libre et toutes les données collectées dans le cadre de ce projet sont soumises à des règles strictes en matière de protection des données. Une fois l'étude terminée, nous vous en communiquerons les résultats si vous le souhaitez.

Pour vous proposer d'ores et déjà un aperçu du projet, voici les points clés à retenir. Vous trouverez à la suite des informations complémentaires plus détaillées.

Pourquoi menons-nous ce projet de recherche ?

Il peut arriver qu'un accouchement soit vécu difficilement, et entraîne des « flashbacks » ou « souvenirs intrusifs ». Il s'agit de souvenirs récurrents et involontaires de la naissance, qui sont parfois source de détresse et peuvent diminuer le bien-être maternel. Avec cette étude, nous souhaiterions savoir si une activité simple sur console, précédée par une brève évocation du vécu de l'accouchement, réduit efficacement le nombre de souvenirs intrusifs liées à cet évènement. Notez que l'évocation de l'accouchement dure environ 10 minutes et qu'elle est superficielle : il ne s'agit en aucun cas de revivre la naissance ou d'entrer dans les détails, et vous êtes libre de ce que vous souhaitez partager.

Que dois-je faire si j'accepte de participer ?

- Si vous acceptez de participer à notre projet, vous devrez signer ce formulaire, venir réaliser l'activité au CHUV et compléter divers questionnaires aux périodes suivantes :
    1. **Avant le rendez-vous**, vous remplirez un journal de bord dans lequel vous reporterez quotidiennement vos souvenirs intrusifs. Le journal de bord est à compléter sur votre smartphone grâce à l'application sécurisée MyCap. Une version papier du journal de bord est également disponible, mais nous vous encourageons à utiliser la version électronique

For peer review only - http://bmjopen.bmj.com/site/about/guidelines.xhtml

UNIL | Université de Lausanne
Institut universitaire de formation et de recherche en soins FBM
FNSNF
FONDS NATIONAL SUISSE
SCHWEIZERISCHER NATIONALFONDS
FONDO NAZIONALE SVIZZERO
SWISS NATIONAL SCIENCE FOUNDATION

en raison de sa practicité. Vous recevrez également par mail une série de questionnaires, qui prennent environ 15 min à remplir.

2. **Lors d'un rendez-vous** d'environ 1h30 au CHUV, vous réaliserez l'activité et répondrez à de brefs questionnaires. Votre fréquence cardiaque sera également relevée à l'aide d'un simple capteur.
3. **Après le rendez-vous**, vous reporterez à nouveau quotidiennement vos souvenirs intrusifs dans un journal de bord, et vous répondrez à quelques questionnaires envoyés par mail, prenant environ 10 min.
4. L'étude se clôture par un bilan, au téléphone, avec un bref questionnaire et un débriefing sur votre participation. Nous proposerons également à certaines participantes de réaliser, au même moment, un entretien au sujet du vécu de leur participation à l'étude.

- Les participantes sont réparties au hasard dans deux groupes qui réaliseront tous les deux l'activité à visée thérapeutique et complèront les journaux de bord sur une durée de 6 semaines. Les deux groupes se différencient concernant l'ordre dans lequel ils remplissent leurs journaux de bord et réalisent l'activité.
- Les informations récoltées dans les journaux de bord permettront de préparer l'activité à visée thérapeutique, de façon à l'adapter au mieux à votre situation et à vos besoins. Elles permettront également de mesurer les bénéfices de l'activité, en les comparant aux souvenirs intrusifs reportés après l'activité.
- La participation à l'étude dure environ 2 mois. Cette durée comprend la complétion des journaux et des questionnaires, le rendez-vous au CHUV et l'entretien téléphonique de fin d'étude.

Quels sont les bénéfices et les risques liés à la participation au projet ?

Bénéfices pour les participants

- La participation à ce projet pourrait potentiellement vous apporter un bénéfice direct, en diminuant le nombre de vos souvenirs intrusifs liés à votre accouchemenent.
- En faisant avancer la recherche scientifique, vous contribuez également au développement d'une technique thérapeutique qui pourrait aider les autres femmes ayant des souvenirs intrusifs à la suite de leur accouchement.

Risques et contraintes

- À notre connaissance, cette étude ne comporte aucun risque particulier pour les participantes. Le rendez-vous au CHUV peut entraîner un inconfort passager du fait de la brève évocation du vécu de l'accouchement.
- Lors de l'appel téléphonique de fin d'étude, si l'activité n'a pas eu d'effet positif pour vous ou que votre vécu de l'accouchement vous fait toujours souffrir, la coordinatrice de l'étude pourra vous indiquer à quel·le·s professionnel·le·s de la périnatalité vous pouvez vous adresser et vous apporter davantage d'information sur les effets psychiques que peuvent entraîner une naissance traumatique.

En apposant votre signature à la fin du document, vous certifiez en avoir compris tout le contenu et consentir librement à prendre part au projet.

For peer review only - http://bmjopen.bmj.com/site/about/guidelines.xhtml

Unil
UNIL | Université de Lausanne
Institut universitaire de formation et de recherche en soins FBM

CHUV

FNSNF
FONDS NATIONAL SUISSE
SCHWEIZERISCHER NATIONALFONDS
FONDO NAZIONALE SVIZZERO
SWISS NATIONAL SCIENCE FOUNDATION

Information détaillée

1. Objectif du projet et sélection des participantes

Dans cette feuille d'information, notre projet de recherche est aussi simplement désigné par le terme *étude*. Si vous acceptez d'y prendre part, vous êtes *une participante à l'étude*.

Il peut arriver qu'un accouchement soit vécu difficilement, et entraîne des « flashbacks » ou « souvenirs intrusifs ». Il s'agit de souvenirs récurrents et involontaires de la naissance, qui sont parfois source de détresse et peuvent diminuer le bien-être maternel. Avec cette étude, nous souhaiterions savoir si une activité simple sur console, précédée par une brève évocation du vécu de l'accouchement, réduit efficacement le nombre de souvenirs intrusifs liées à cet évènement. Notez que l'évocation de l'accouchement dure environ 10 minutes et qu'elle est superficielle : il ne s'agit en aucun cas de revivre la naissance ou de rentrer dans les détails, et vous êtes libre de ce que vous souhaitez partager.

La participation est ouverte aux femmes ayant accouché au CHUV dès la 6ème semaine postpartum et qui nous ont donné leur consentement écrit. Afin de pouvoir observer une potentielle amélioration à la suite de la réalisation de l'activité, nous incluons dans l'étude les femmes rapportant des souvenirs intrusifs réguliers liés à leur accouchement. Si vous n'êtes pas sûre du nombre de vos souvenirs intrusifs, nous vous proposerons de remplir un premier journal de bord afin d'évaluer leur nombre. Si vous n'avez pas rapportez de souvenirs intrusifs réguliers à la fin de celui-ci, nous vous proposerons tout de même de réaliser l'activité bien que vos données ne pourront pas être utilisées dans le cadre de notre étude.

La participation à l'étude concerne les femmes ayant un niveau de français suffisant pour compléter les différentes étapes de l'étude, qui ne prennent pas de médicament contenant du propranolol et qui ne souffrent pas, elles et leur(s) enfant(s), d'une maladie sévère. Les femmes étant dans l'incapacité de discerner leurs souvenirs intrusifs liés à leur accouchement de souvenirs intrusifs liés à d'autres événements, ou ayant des antécédents d'abus d'alcool ou de drogues ne peuvent pas prendre part à cette étude. En effet, nous pensons que ces expériences doivent être étudiées de façon séparée. Pour les mêmes raisons, les femmes suivant un traitement psychologique en lien avec leur accouchement au moment du recrutement ne sont pas invitées à participer à l'étude.

2. Informations générales sur le projet

Cette étude est monocentrique car elle est conduite dans un seul centre de recherche, à Lausanne. Cette étude mobilisera environ 90 femmes au total. Votre participation à l'étude sera d'environ 2 mois. L'activité, qui dure environ 1h30, se déroulera au CHUV avec une psychologue. Si vous participez à l'étude, vous serez affectée au hasard dans un des deux groupes qui réaliseront tout deux l'activité à visée thérapeutique. Ces groupes se différencient uniquement concernant l'ordre de réalisation des journaux de bord et de l'activité (le déroulé des étapes est décrit en détail au point 3.). Afin de préparer le rendez-vous au CHUV, il sera demandé aux participantes de remplir un journal de bord dans lequel elles rapporteront leurs souvenirs intrusifs liés à leur accouchement, avant puis après l'activité.

Cette étude est réalisée dans le respect des prescriptions de la législation suisse. Nous suivons en outre l'ensemble des directives reconnues au niveau international. La commission d'éthique compétente a examiné et autorisé l'étude. Vous trouverez également un descriptif de l'étude sur le site Internet de l'Office fédéral de la santé publique (www.kofam.ch) (SNCTP000004935) et sur ClinicalTrials.gov.

3. Déroulement pour les participantes

- Si vous acceptez de participer, vous signerez le formulaire de consentement à la fin de ce document. Vous devrez ensuite réaliser les étapes rapportées ci-dessous :

For peer review only - http://bmjopen.bmj.com/site/about/guidelines.xhtml

UNIL | Université de Lausanne
Institut universitaire de formation et de recherche en soins FBM
CHUV
FNS NF
Fonds national suisse
Schweizerischer Nationalfonds
Fondo nazionale svizzero
Swiss National Science Foundation

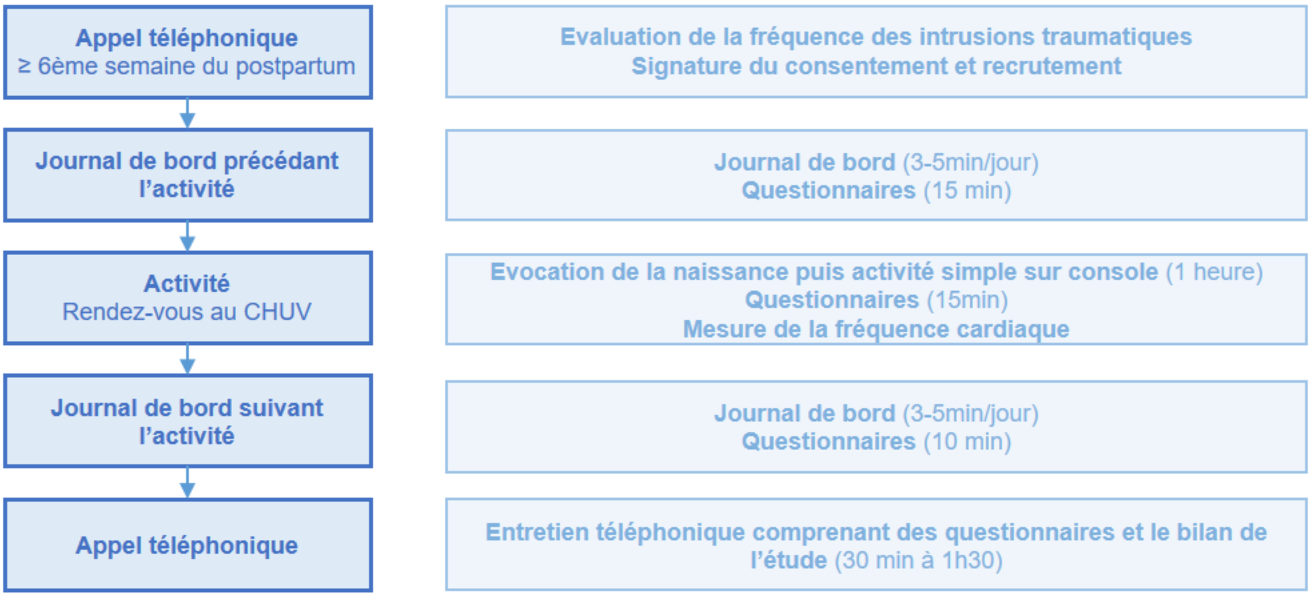

- Au minimum 6 semaines après votre accouchement, vous recevrez un lien vers des questionnaires en ligne (10 minutes). Nous vous demanderons ensuite de reporter dans un journal de bord vos souvenirs intrusifs (3 à 5 minutes/jour). Nous vous enverrons des questionnaires en ligne à différents moments du journal (5 minutes).
- À la fin du premier journal, vous aurez rendez-vous au CHUV. Il commencera par une brève évocation de votre accouchement, pendant 10 minutes. Ensuite, vous ferez une activité simple sur console, durant 20 minutes. Celle-ci n'a pas de rapport avec votre accouchement, elle ne nécessite aucune compétence particulière. Vous devrez également répondre à de brefs questionnaires (5 minutes), et votre fréquence cardiaque sera mesurée lors du rendez-vous grâce à un dispositif non-invasif.
- À la suite du rendez-vous, vous remplirez de nouveau un journal de bord et répondrez à un bref questionnaire (5 minutes).
- Un mois après le rendez-vous, vous recevrez un questionnaire en ligne (5 minutes).
- À la fin du dernier journal, suite à un bref questionnaire, nous ferons le bilan de l'étude et de l'évolution de vos souvenirs intrusifs, lors d'un dernier appel téléphonique durant environ 30 minutes. Nous proposerons également à certaines participantes de réaliser un entretien, lors de cet appel, au sujet du vécu de leur participation à l'étude et des potentiels bénéfices qu'elles ont observés à la suite du rendez-vous. Cet entretien prend environ 1 heure et a pour objectif de permettre à l'équipe de recherche de mieux comprendre votre ressenti de l'étude. Son contenu audio sera enregistré sur un serveur sécurisé puis supprimé une fois que les données seront retranscrites et codées. Si la participation à cet entretien vous intéresse, vous pouvez nous le signaler en cochant la case correspondante à la fin de ce feuillet. Si vous ne souhaitez pas y prendre part, cela n'affectera pas le reste de votre participation à l'étude.

4. Bénéfices pour les participantes

Si vous participez à l'étude, cela pourrait potentiellement diminuer le nombre de vos souvenirs intrusifs en lien avec votre accouchement. En effet, plusieurs études précédentes ont suggéré que cette activité que nous testons pourrait les réduire, immédiatement après un événement difficile ou même plusieurs mois voire années après celui-ci. Si l'activité est sans effet ou que votre vécu de l'accouchement vous fait encore souffrir, la coordinatrice de l'étude pourra vous indiquer à quel·le·s professionnel·le·s de la périnatalité vous pouvez vous adresser, et vous apporter davantage d'information sur les conséquences psychiques qu'une naissance traumatique peut avoir. Par ailleurs, les résultats de l'étude pourraient se révéler importants par la suite, pour d'autres femmes ayant également des souvenirs intrusifs suite à leur accouchement.

For peer review only - http://bmjopen.bmj.com/site/about/guidelines.xhtml

UNIL | Université de Lausanne
Institut universitaire de formation et de recherche en soins FBM
CHUV
FNSNF
Fonds national suisse
Schweizerischer Nationalfonds
Fondo nazionale svizzero
Swiss National Science Foundation

5. Caractère facultatif de la participation et obligations

Votre participation est entièrement libre. Si vous choisissez de ne pas participer ou si vous choisissez de participer et revenez sur votre décision pendant le déroulement de l'étude, vous n'aurez pas à vous justifier. Cela ne changera rien à votre prise en charge médicale habituelle. Vous pouvez à tout moment poser des questions sur l'étude à la Prof. Antje Horsch ou à Mme Déborah Fort (cf. coordonnées à la fin du document).

En tant que participante à l'étude, vous serez tenue de suivre les instructions liées à la procédure de l'étude et de remplir les journaux de bord et les questionnaires, ainsi que de vous présenter lors du rendez-vous. En cas d'augmentation de la fréquence de vos souvenirs intrusifs ou si vous débutez un traitement ou une thérapie en lien avec votre vécu de l'accouchement, nous vous prierons d'en informer la Prof. Antje Horsch ou Mme Déborah Fort.

6. Risques et contraintes pour les participantes

À notre connaissance, cette étude ne comporte aucun risque particulier pour les participantes. Le rendez-vous au CHUV peut entraîner un inconfort passager du fait de la brève évocation de l'accouchement.

7. Alternatives

Si vous décidez de ne pas prendre part à l'étude, il sera possible de vous adresser à un·e psychothérapeute connaissant les troubles psychiques liés aux expériences difficiles. Si vous refusez de participer à cette étude, ou décidez d'interrompre votre participation, cela n'aura aucune conséquence sur votre suivi médical.

8. Résultats

L'étude permet d'obtenir différents résultats :

1. Des résultats individuels qui vous concernent directement (p. ex. comment vos souvenirs intrusifs ont évolué suite à l'activité),
2. Les résultats globaux de l'étude (p. ex. comment les souvenirs intrusifs de l'ensemble des participantes ont évolué suite à l'activité).

L'investigatrice vous avisera, pendant l'étude, de toute nouvelle découverte importante vous concernant. Vous serez informée oralement et par écrit ; vous pourrez par la suite à nouveau décider si vous souhaitez poursuivre votre participation à l'étude. Si vous ne souhaitez pas être mise au courant de ces éventualités, merci de l'indiquer aux personnes responsables de l'étude.

L'investigatrice peut vous faire parvenir, à l'issue de l'étude, une synthèse des résultats globaux si vous le souhaitez.

9. Confidentialité des données

9.1. Traitement et codage des données

Dans le cadre de cette étude, des données relatives à votre personne et à votre santé sont codées au moment du relevé. Le codage signifie que toutes les données permettant de vous identifier (nom, date de naissance, etc.) sont remplacées par un code. Il n'est pas possible de relier les données à votre personne sans le code, qui restent en permanence au sein des dossiers de l'équipe de recherche, stockées sur les serveurs sécurisés du CHUV.

For peer review only - http://bmjopen.bmj.com/site/about/guidelines.xhtml

UNIL | Université de Lausanne
Institut universitaire de formation et de recherche en soins FBM

FONDS NATIONAL SUISSE
SCHWEIZERISCHER NATIONALFONDS
FONDO NAZIONALE SVIZZERO
SWISS NATIONAL SCIENCE FOUNDATION

Seul un nombre limité de personnes peut consulter vos données sous une forme non codée, et ce, exclusivement afin de pouvoir accomplir des tâches nécessaires au déroulement de l'étude. Ces personnes sont tenues au secret professionnel. En tant que participante, vous avez le droit de consulter vos données.

9.2. Protection des données

Toutes les directives relatives à la protection des données sont rigoureusement respectées.

Les données à l'origine des publications qui découleront de cette étude vont être publiées sur un site dédié ('Open Data') sous une forme codée (https://zenodo.org/). Dans le cadre d'un tel partage, vos données seront toujours codées de façon qu'il ne soit pas possible de remonter à votre identité. Cette démarche de partage des données permet la validation des résultats publiés (reproductibilité), de cumuler les données de différentes recherches et plus généralement l'utilisation des données par d'autres chercheurs. En cas de retrait de votre consentement à participer à ce projet, les données déjà partagées ne pourront pas être retirées. Vous devez être d'accord avec cela si vous participez à l'étude.

9.3. Droit de consultation dans le cadre d'inspections

L'étude peut faire l'objet d'inspections. Celles-ci peuvent être effectuées par la commission d'éthique compétente ou par le promoteur qui a initié l'étude. L'investigatrice doit alors communiquer vos données pour les besoins de ces inspections. Toutes les personnes impliquées sont tenues au plus strict secret professionnel.

10. Retrait du projet

Vous pouvez à tout moment vous retirer de l'étude si vous le souhaitez. Cependant, les données jusque-là pourront encore être analysés sous forme codée. En cas de retrait, vos données continuent de figurer sous forme codée dans les documents de l'étude. Vous devez donc être d'accord avec cela avant de donner votre consentement.

11. Dédommagement

Les dépenses liées aux frais de transport (transports publics ou tickets de parking) qui découlent directement de la participation à l'étude vous seront remboursées sous présentation des documents originaux. Votre participation n'aura aucune conséquence financière pour vous ou votre assurance maladie.

12. Responsabilité

Le CHUV qui a initié le projet de recherche et est chargé de sa réalisation, est responsable des dommages que vous pourriez subir en relation avec les activités de recherche. Les conditions et la procédure sont fixées par la loi. Bien que ces recherches ne présentent pas de risque prévisible, l'institution (le promoteur) est responsable, en vertu des dispositions légales, de tout dommage pouvant survenir dans le cadre de l'étude. Si vous subissiez un dommage du fait de votre participation à l'étude, il vous faudrait vous adresser à l'investigatrice mentionnée ci-avant.

13. Financement

L'étude est financée par le Fond National pour la recherche Suisse (SNF IZCOZ0_205434/1).

14. Interlocutrices

Vous pouvez à tout moment poser des questions au sujet de l'étude. En cas de doutes, de craintes ou d'urgences pendant ou après l'étude, vous pouvez vous adresser à l'une des interlocutrices suivantes :

For peer review only - http://bmjopen.bmj.com/site/about/guidelines.xhtml

Prof. Antje Horsch : Directrice de l'étude

Institut Universitaire de Formation et Recherche en Soins, Université de Lausanne et Département Femme-Mère-Enfant, CHUV.
Route de la Corniche 10
Biopôle 2
1010 Lausanne
antje.horsch@chuv.ch
079/556.07.50

Mme Déborah Fort : Doctorante et coordinatrice de l'étude

Institut Universitaire de Formation et Recherche en Soins, Université de Lausanne
Route de la Corniche 10
Biopôle 2
1010 Lausanne
deborah.fort@chuv.ch
079/556.73.37

For peer review only

For peer review only - http://bmjopen.bmj.com/site/about/guidelines.xhtml

UNIL | Université de Lausanne
Institut universitaire de formation et de recherche en soins FBM
CHUV
FNSNF
Fonds national suisse
Schweizerischer Nationalfonds
Fondo nazionale svizzero
Swiss National Science Foundation

Déclaration de consentement

Déclaration de consentement écrite pour la participation à une étude clinique
Veuillez lire attentivement ce formulaire. N'hésitez pas à poser des questions lorsque vous ne comprenez pas quelque chose ou que vous souhaitez avoir des précisions. Votre consentement écrit est nécessaire pour participer au projet.

**Numéro BASEC du projet de recherche :**	2022-00652
**Titre (scientifique et usuel) :**	*tAckling intruSive Traumatic memoRies After chiLdbirth (ASTRAL): A single-blinded waitlist randomized controlled trial*<br>Etude ASTRAL : Lutter contre les souvenirs intrusifs après un accouchement difficile
**Institution responsable (promoteur et adresse complète) :**	Prof. Antje Horsch<br>Institut Universitaire de Formation et Recherche en Soins, Université de Lausanne et CHUV, Biopôle 2. Route de la Corniche 10 - 1010 Lausanne
**Lieu de réalisation :**	CHUV
**Responsables sur le site :**<br>Nom et prénom en caractères d'imprimerie :	Prof. Antje Horsch
**Participant / Participante :**<br>Nom et prénom en caractères d'imprimerie :<br>Date de naissance :	____________________<br>____________________

- Je déclare avoir été informée, par la responsable soussignée, oralement et par écrit, des objectifs et du déroulement de l'étude ainsi que des avantages et des inconvénients possibles et des risques éventuels.
- Je prends part à cette étude de façon volontaire et j'accepte le contenu de la feuille d'information qui m'a été remise. J'ai eu suffisamment de temps pour prendre ma décision.
- J'ai reçu les réponses aux questions que j'ai posées en relation avec ma participation à cette étude. Je conserve la feuille d'information et reçois une copie de ma déclaration de consentement écrite.
- J'ai été informée de l'existence possible d'autres traitements et thérapies.
- J'accepte que les spécialistes compétents du promoteur de ce projet, de la commission d'éthique compétente puissent consulter mes données non codées afin de procéder à des contrôles et des inspections, à condition toutefois que la confidentialité de ces données soit strictement assurée.
- Je serai informée des résultats ayant une incidence directe sur ma santé. Si je ne souhaite pas obtenir ces informations, je prends contact avec la directrice de l'étude.
- Je sais que j'ai à tout moment le droit de consulter mes propres données.
- Je sais que mes données personnelles peuvent être transmises à des fins de recherche dans le cadre de ce projet uniquement et sous une forme codée.
- Je suis d'accord pour que mes données dûment codées soient partagées à des fins de recherche en accès libre sur des bases de données biomédicales.
- J'ai compris que mes données codées seront partagées en open data et qu'en cas de retrait de mon consentement, celles-ci ne pourront pas être retirées.
- Je peux, à tout moment et sans avoir à me justifier, révoquer mon consentement à participer à l'étude, sans que cette décision n'ait de répercussions défavorables sur la suite de ma prise en charge. Les données qui ont été recueillies jusque-là seront cependant analysées dans le cadre de l'étude.
- Je suis informée que l'assurance-responsabilité civile du CHUV couvre les dommages éventuels imputables au projet.
- Je suis consciente que les obligations mentionnées dans la feuille d'information destinée aux participantes doivent être respectées pendant toute la durée de l'étude. La directrice de l'étude peut m'exclure à tout moment de l'étude dans l'intérêt de ma santé.
- Je suis d'accord pour être recontactée par les membres de l'équipe de recherche pour de futures études. Il reste entendu que je peux révoquer en tout temps cette autorisation. Si je ne suis pas d'accord, je peux toujours participer à l'étude.

For peer review only - http://bmjopen.bmj.com/site/about/guidelines.xhtml

Unil UNIL | Université de Lausanne
Institut universitaire de formation et de recherche en soins FBM

CHUV

FNSNF
FONDS NATIONAL SUISSE
SCHWEIZERISCHER NATIONALFONDS
FONDO NAZIONALE SVIZZERO
SWISS NATIONAL SCIENCE FOUNDATION

Merci de cocher la case suivante si vous êtes intéressée à participer à l'entretien de fin d'étude :

☐ Je suis d'accord pour que l'appel audio effectué lors de l'entretien de fin d'étude soit enregistré afin de permettre à l'équipe de recherche de mieux comprendre mon ressenti sur l'étude.

Lieu, date	Signature du participant / de la participante
____________________	____

Attestation de la personne investigatrice : Par la présente, j'atteste avoir expliqué au participant/à la participante la nature, l'importance et la portée de l'étude. Je déclare satisfaire à toutes les obligations en relation avec cette étude conformément au droit suisse en vigueur. Si je devais prendre connaissance, à quelque moment que ce soit durant la réalisation de l'étude, d'éléments susceptibles d'influer sur le consentement du participant/de la participante à prendre part au projet, je m'engage à l'en informer immédiatement.

Lieu, date	Nom et prénom de la personne investigatrice en caractères d'imprimerie.
____________________	____
	Signature de la personne investigatrice
	____

For peer review only

For peer review only - http://bmjopen.bmj.com/site/about/guidelines.xhtml

SPIRIT

STANDARD PROTOCOL ITEMS: RECOMMENDATIONS FOR INTERVENTIONAL TRIALS

SPIRIT 2013 Checklist: Recommended items to address in a clinical trial protocol and related documents*

Section/item	Item No	Description	Addressed on page number
**Administrative information**			
Title	1	Descriptive title identifying the study design, population, interventions, and, if applicable, trial acronym	1
Trial registration	2a	Trial identifier and registry name. If not yet registered, name of intended registry	17
	2b	All items from the World Health Organization Trial Registration Data Set	1-21
Protocol version	3	Date and version identifier	17
Funding	4	Sources and types of financial, material, and other support	18
Roles and responsibilities	5a	Names, affiliations, and roles of protocol contributors	1-2 & 18
	5b	Name and contact information for the trial sponsor	17
	5c	Role of study sponsor and funders, if any, in study design; collection, management, analysis, and interpretation of data; writing of the report; and the decision to submit the report for publication, including whether they will have ultimate authority over any of these activities	Not applicable
	5d	Composition, roles, and responsibilities of the coordinating centre, steering committee, endpoint adjudication committee, data management team, and other individuals or groups overseeing the trial, if applicable (see Item 21a for data monitoring committee)	17-18

For peer review only

Introduction

Background and rationale	6a	Description of research question and justification for undertaking the trial, including summary of relevant studies (published and unpublished) examining benefits and harms for each intervention	5-8
	6b	Explanation for choice of comparators	7
Objectives	7	Specific objectives or hypotheses	11-12
Trial design	8	Description of trial design including type of trial (eg, parallel group, crossover, factorial, single group), allocation ratio, and framework (eg, superiority, equivalence, noninferiority, exploratory)	8 & 9

Methods: Participants, interventions, and outcomes

Study setting	9	Description of study settings (eg, community clinic, academic hospital) and list of countries where data will be collected. Reference to where list of study sites can be obtained	8
Eligibility criteria	10	Inclusion and exclusion criteria for participants. If applicable, eligibility criteria for study centres and individuals who will perform the interventions (eg, surgeons, psychotherapists)	8-9 & 10
Interventions	11a	Interventions for each group with sufficient detail to allow replication, including how and when they will be administered	9-11
	11b	Criteria for discontinuing or modifying allocated interventions for a given trial participant (eg, drug dose change in response to harms, participant request, or improving/worsening disease)	Not applicable
	11c	Strategies to improve adherence to intervention protocols, and any procedures for monitoring adherence (eg, drug tablet return, laboratory tests)	9-11 & 16
	11d	Relevant concomitant care and interventions that are permitted or prohibited during the trial	8

For peer review only - http://bmjopen.bmj.com/site/about/guidelines.xhtml

Outcomes	12	Primary, secondary, and other outcomes, including the specific measurement variable (eg, systolic blood pressure), analysis metric (eg, change from baseline, final value, time to event), method of aggregation (eg, median, proportion), and time point for each outcome. Explanation of the clinical relevance of chosen efficacy and harm outcomes is strongly recommended	5, 11-12, & 14-15
Participant timeline	13	Time schedule of enrolment, interventions (including any run-ins and washouts), assessments, and visits for participants. A schematic diagram is highly recommended (see Figure)	9-10, 16 and Figure 1
Sample size	14	Estimated number of participants needed to achieve study objectives and how it was determined, including clinical and statistical assumptions supporting any sample size calculations	16
Recruitment	15	Strategies for achieving adequate participant enrolment to reach target sample size	9
**Methods: Assignment of interventions (for controlled trials)**			
Allocation:			
Sequence generation	16a	Method of generating the allocation sequence (eg, computer-generated random numbers), and list of any factors for stratification. To reduce predictability of a random sequence, details of any planned restriction (eg, blocking) should be provided in a separate document that is unavailable to those who enrol participants or assign interventions	9
Allocation concealment mechanism	16b	Mechanism of implementing the allocation sequence (eg, central telephone; sequentially numbered, opaque, sealed envelopes), describing any steps to conceal the sequence until interventions are assigned	9
Implementation	16c	Who will generate the allocation sequence, who will enrol participants, and who will assign participants to interventions	9
Blinding (masking)	17a	Who will be blinded after assignment to interventions (eg, trial participants, care providers, outcome assessors, data analysts), and how	9 & 17

	17b	If blinded, circumstances under which unblinding is permissible, and procedure for revealing a participant's allocated intervention during the trial	Not applicable
**Methods: Data collection, management, and analysis**			
Data collection methods	18a	Plans for assessment and collection of outcome, baseline, and other trial data, including any related processes to promote data quality (eg, duplicate measurements, training of assessors) and a description of study instruments (eg, questionnaires, laboratory tests) along with their reliability and validity, if known. Reference to where data collection forms can be found, if not in the protocol	10, 14-15 & 16-17
	18b	Plans to promote participant retention and complete follow-up, including list of any outcome data to be collected for participants who discontinue or deviate from intervention protocols	9 & Not applicable
Data management	19	Plans for data entry, coding, security, and storage, including any related processes to promote data quality (eg, double data entry; range checks for data values). Reference to where details of data management procedures can be found, if not in the protocol	16-17
Statistical methods	20a	Statistical methods for analysing primary and secondary outcomes. Reference to where other details of the statistical analysis plan can be found, if not in the protocol	16-17
	20b	Methods for any additional analyses (eg, subgroup and adjusted analyses)	16-17
	20c	Definition of analysis population relating to protocol non-adherence (eg, as randomised analysis), and any statistical methods to handle missing data (eg, multiple imputation)	16-17
**Methods: Monitoring**			
Data monitoring	21a	Composition of data monitoring committee (DMC); summary of its role and reporting structure; statement of whether it is independent from the sponsor and competing interests; and reference to where further details about its charter can be found, if not in the protocol. Alternatively, an explanation of why a DMC is not needed	17

	21b	Description of any interim analyses and stopping guidelines, including who will have access to these interim results and make the final decision to terminate the trial	Not applicable
Harms	22	Plans for collecting, assessing, reporting, and managing solicited and spontaneously reported adverse events and other unintended effects of trial interventions or trial conduct	17
Auditing	23	Frequency and procedures for auditing trial conduct, if any, and whether the process will be independent from investigators and the sponsor	17
**Ethics and dissemination**			
Research ethics approval	24	Plans for seeking research ethics committee/institutional review board (REC/IRB) approval	17
Protocol amendments	25	Plans for communicating important protocol modifications (eg, changes to eligibility criteria, outcomes, analyses) to relevant parties (eg, investigators, REC/IRBs, trial participants, trial registries, journals, regulators)	17
Consent or assent	26a	Who will obtain informed consent or assent from potential trial participants or authorised surrogates, and how (see Item 32)	9
	26b	Additional consent provisions for collection and use of participant data and biological specimens in ancillary studies, if applicable	Supplemental Material p. 6
Confidentiality	27	How personal information about potential and enrolled participants will be collected, shared, and maintained in order to protect confidentiality before, during, and after the trial	17
Declaration of interests	28	Financial and other competing interests for principal investigators for the overall trial and each study site	18
Access to data	29	Statement of who will have access to the final trial dataset, and disclosure of contractual agreements that limit such access for investigators	16-17
Ancillary and post-trial care	30	Provisions, if any, for ancillary and post-trial care, and for compensation to those who suffer harm from trial participation	Not applicable

For peer review only

Dissemination policy	31a	Plans for investigators and sponsor to communicate trial results to participants, healthcare professionals, the public, and other relevant groups (eg, via publication, reporting in results databases, or other data sharing arrangements), including any publication restrictions	17
	31b	Authorship eligibility guidelines and any intended use of professional writers	18
	31c	Plans, if any, for granting public access to the full protocol, participant-level dataset, and statistical code	17
**Appendices**			
Informed consent materials	32	Model consent form and other related documentation given to participants and authorised surrogates	Supplemental material
Biological specimens	33	Plans for collection, laboratory evaluation, and storage of biological specimens for genetic or molecular analysis in the current trial and for future use in ancillary studies, if applicable	Not applicable

*It is strongly recommended that this checklist be read in conjunction with the SPIRIT 2013 Explanation & Elaboration for important clarification on the items. Amendments to the protocol should be tracked and dated. The SPIRIT checklist is copyrighted by the SPIRIT Group under the Creative Commons "Attribution-NonCommercial-NoDerivs 3.0 Unported" license.

For peer review only
