# Supplementary material for: Tackling childbirth-related intrusive memories with a single-session behavioural intervention involving a visuospatial task: protocol for a single-blind, waitlist-controlled randomised trial
Source: BMJ Open. 2023 May 29;13(5):e073874. doi: 10.1136/bmjopen-2023-073874 (PMC10230990; doi:10.1136/bmjopen-2023-073874)
Supplement: Reviewer comments [file bmjopen-2023-073874.reviewer_comments.pdf]

PEER REVIEW HISTORY

BMJ Open publishes all reviews undertaken for accepted manuscripts. Reviewers are asked to complete a checklist review form (**http://bmjopen.bmj.com/site/about/resources/checklist.pdf**) and are provided with free text boxes to elaborate on their assessment. These free text comments are reproduced below.

ARTICLE DETAILS

**TITLE (PROVISIONAL)**	Tackling childbirth-related intrusive memories with a single-session behavioural intervention including a visuospatial task: protocol for a single-blind, waitlist-controlled randomised trial
**AUTHORS**	Fort, Déborah; Deforges, Camille; Messerli-Bürgy, Nadine; Michael, Tanja; Baud, David; Lalor, Joan; Rimmele, Ulrike; Horsch, Antje

This article was not externally reviewed at BMJ Open. Protocols that have been independently assessed prior to submission to BMJ Open are usually fast-tracked to publication on the grounds that further substantial changes will not be possible. This independent assessment will usually be external, independent review for both a substantial grant award from a non-commercial or government funder and ethics approval.
